# New Series of Thiazole Derivatives: Synthesis, Structural Elucidation, Antimicrobial Activity, Molecular Modeling and MOE Docking

**DOI:** 10.3390/molecules24091741

**Published:** 2019-05-04

**Authors:** Ismail Althagafi, Nashwa El-Metwaly, Thoraya A. Farghaly

**Affiliations:** 1 Chemistry Department, Faculty of Applied Science, Umm Al-Qura University, Makkah 21514, Saudi Arabia; n_elmetwaly00@yahoo.com; 2 Chemistry Department, Faculty of Science, Mansoura University, Mansoura 002050, Egypt; 3 Chemistry Department, Faculty of Science, Cairo University, Giza 12613, Egypt

**Keywords:** dithiazoles, trithiazoles, tetrathiazoles, antimicrobial activity, modeling, docking study

## Abstract

Based on the extensive biological activities of thiazole derivatives against different types of diseases, we are interested in the effective part of many natural compounds, so we synthesized a new series of compounds containing di-, tri- and tetrathiazole moieties. The formation of such derivatives proceeded via reaction of 2-bromo-1-(4-methyl-2-(methylamino)thiazol-5-yl)ethan-1-one with heterocyclic amines, *o*-aminothiophenol and thiosemicarbazone derivatives. The structure and mechanistic pathways for all products were discussed and proved based on spectral results, in addition to conformational studies. Our aim after the synthesis is to investigate their antimicrobial activity against various types of bacteria and fungi species. Preceeding such an investigation, a molecular docking study was carried out with selected conformers, as representative examples, against three pathogen-proteins. This preliminary stage could support the biological application. The potency of these compounds as antimicrobial agents has been evaluated. The results showed that derivatives which have di- and trithiazole rings displayed high activity that exceeds the used standard antibiotic.

## 1. Introduction

**Figure 1:**
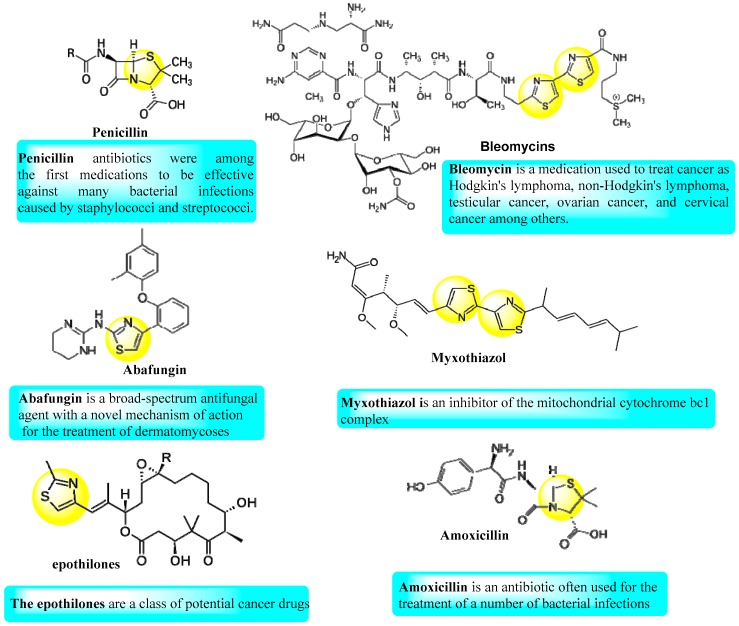
Natural and available marketing drugs containing thiazole rings.

It is not coincidental that the first effective antibiotics used to treat microbes (the penicillins, Figure 1) contains a thiazole ring. Also, several commercial drugs for various types of diseases that contain thiazole moieties, were synthesized, as illustrated in Figure 1. Thiazole derivatives are actually a considerable group of heterocyclic compound that have therapeutic effects against several diseases [1,2]. The most pertinent and modern studies have manifested that these molecules display antifungal [3], antibacterial [4], anti-inflammatory [5,6], analgesic [7], and anti-cancer [8,9] activities. Many patents have been registered for thiazole compounds with antimicrobial activity [10,11]. It has been demonstrated that the design and synthesis of organic compounds containing more than one thiazole ring unit enhanced their therapeutic activities [12,13,14,15]. Thus, many researchers have become interested in the synthesis of molecules containing more than one thiazole moiety [16,17]. Also, as a result of persistent microbial resistance to antibiotics, chemical and pharmaceutical researchers are constantly seeking to discover and synthesize alternatives to known antibiotics. Thus, from viewpoint of the promising antimicrobial activity of thiazoles and in continuation to our efforts concerning the synthesis of bioactive heterocyclic compounds [18,19,20,21,22,23,24], herein, we aimed to synthesize a new series of compounds having more than one thiazole ring. This was executed via the reaction of 2-bromo-1-(4-methyl-2-(methylamino)thiazol-5-yl)ethan-1-one with different heterocyclic amines and thiosemicarbazones. Due to the possibility of formation of more than one isomer from the reaction of heterocyclic amines with the phenacyl bromide derivative, we aimed to elucidate the actual structural forms through various techniques. Moreover, the antimicrobial activity of the products was screened against different types of bacteria and fungi and simulated by docking processes for some chosen conformers.

## 2. Results and Discussion

### 2.1. Chemistry

**Scheme 1:**
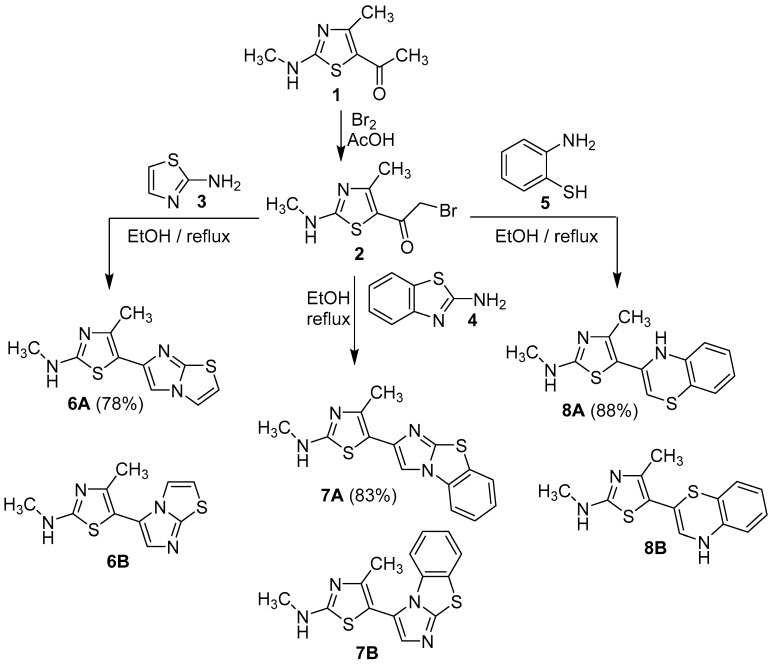
Synthesis of thiazole derivatives **6**–**8**.

**Scheme 2:**
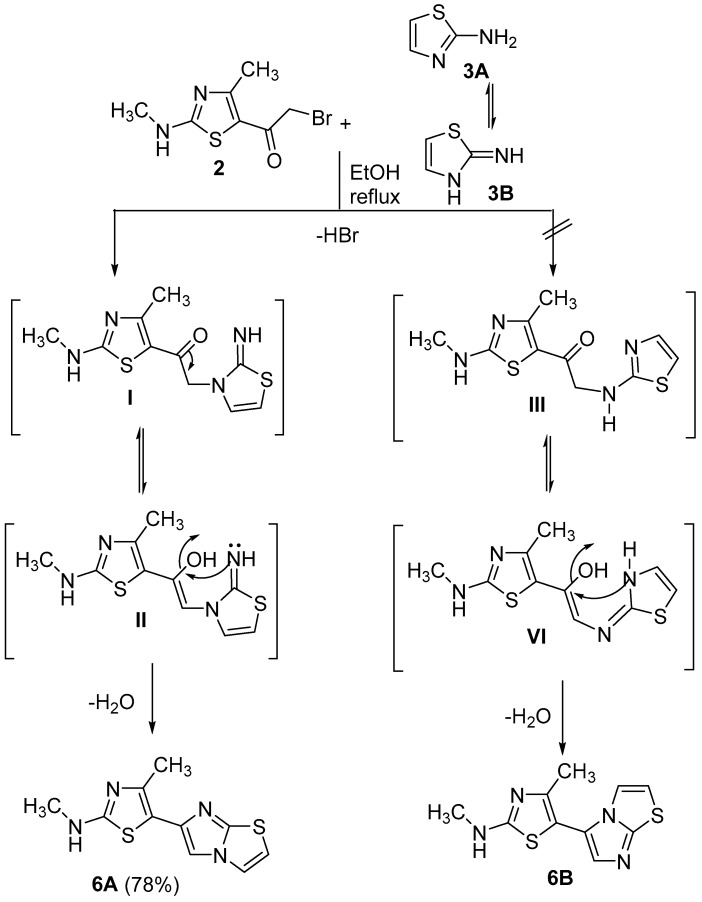
The mechanism of the formation of isomer **6A**.

The starting material required in this study was 2-bromo-1-(4-methyl-2-(methylamino)thiazol-5-yl)ethan-1-one (**2**), which was prepared through bromination of 5-acetyl-4-methyl-2-(methylamino)thiazole (**1**) in acidic medium, as previously reported [25] (Scheme 1). The phenacyl bromide derivative **2** was reacted with a mole equivalent of 2-aminothiazole (**3**) in ethanol under reflux to give one of two possible isomers **6A** or **6B** as indicated by the presence of only one spot in TLC. The spectral data (IR, MS) and elemental analysis cannot distinguish the actual product among the suggested possibilities. ^1^H-NMR of the isolated product showed a remarkable singlet at *δ* = 8.20 ppm for the imidazole proton. The high value of such a chemical shift indicated that this =CH is adjacent to a sp^3^ hybridized nitrogen atom [26,27], which is possible in isomer **6A** rather than isomer **6B**. As illustrated in Scheme 2, the reaction proceeds via the formation of an iminothiadiazole intermediate **I**, followed by in-situ dehydrative cyclization to form the desired fused heterocycle **6A** [19]. Dalimba et al., also proved this route using X-ray crystallographic analysis, that indicated the elimination of HBr from the endo-NH group, while, the exocyclic-NH group condensed with a carbonyl group to lose H_2_O [27]. 2-Aminobenzothiazole (**4**) reacted in a similar way with compound **2** to afford 5-(benzo[*d*]imidazo [2,1-*b*]thiazol-2-yl)-*N*,4-dimethylthiazol-2-amine (**7A**). The other possible isomer **7B** (Scheme 1), was discarded based on ^1^H-NMR data and a conformational study, that will discussed later. As for instance, the ^1^H-NMR spectrum of the isolated product showed a characteristic singlet signal at *δ* = 8.76 ppm for the imidazole-H proton of isomer **7A**. 5-(4*H*-Benzo[*b*][1,4]thiazin-3-yl)-*N*,4-dimethylthiazol-2-amine (**8A**) was formed via reaction of 2-aminothiophenol with 2-bromo-1-(4-methyl-2-(methylamino)thiazol-5-yl)ethan-1-one (**2**) in ethanol under reflux (Scheme 1). The formation of isomer **8A** instead of **8B** agrees with the fact that the nucleophilicity of the sulfur atom is more than that of the NH_2_ group thus, the reaction started with S-alkylation with the elimination of HBr, followed by concurrent removal of a water molecule from NH_2_ and the carbonyl group. Moreover, the mass spectra of new derivatives **6**, **7** and **8** displayed the correct molecular ions as suggested for their molecular formulae (see Experimental part). Furthermore, the IR spectra for all derivatives **6**–**8** were free from any absorption bands characteristic for C=O, but, revealed the presence of two characteristic bands for NH and C=N at 3417-3238 and 1634-1612 cm^−1^, respectively. 

**Scheme 3:**
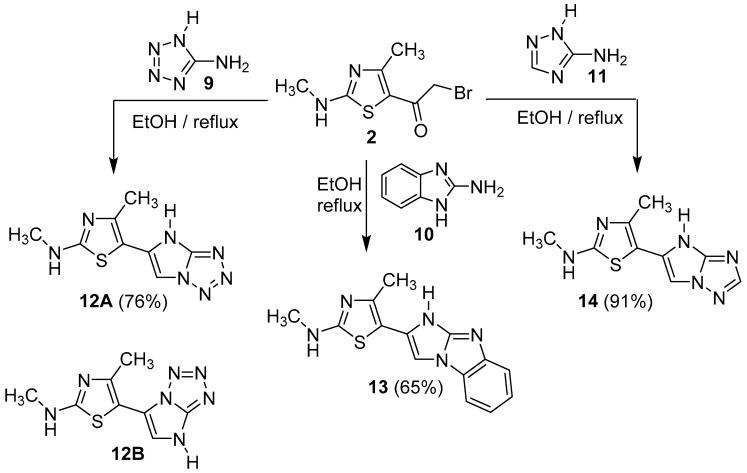
Synthesis of compounds **12**–**14**.

Other heterocyclic amines namely, 5-aminotetrazole **9**, 2-aminobenzimidazole **10**, and 3-amino-1,2,4-triazole **11** reacted with compound **2** to give new series of thiazole rings carrying different azoles at position 5, indicated by numbers **12**–**14**, respectively (Scheme 3). In this case, none of the spectral data (MS, IR and NMR) can discriminate between the two possible isomers **12A** and **12B**, while the structural modeling study can do that. For instance, its IR-data are in agreement with the two suggested structures **12A** and **12B**, which are free from any C=O absorption bands and have notable bands for NH and C=N groups. 

**Scheme 4:**
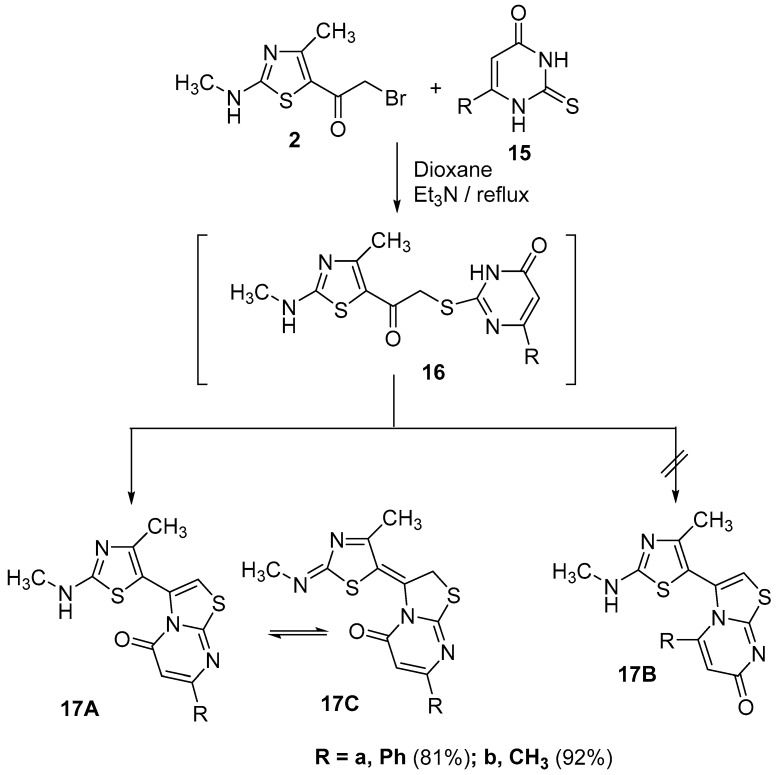
Reaction of α-bromoketone derivative **2** with pyrimidine thione **15a**,**b**.

**Figure 2:**
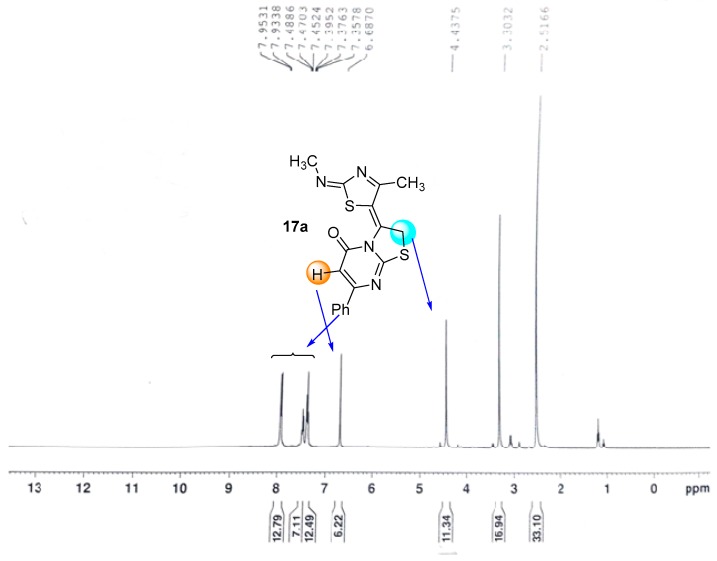
^1^H-NMR spectrum of compound **17a** which exists in the tautomeric form C.

Based on the high reactivity of α-bromoketone derivative **2**, it was further reacted with pyrimidine thiones **15a**,**b** in dioxane in the presence of a catalytic amount of Et_3_N to afford one of three isomers **17A**, **17B** or **17C** (Scheme 4). The preliminary choice for the actual product of reaction, was based on the ^13^C-NMR spectrum, that revealed the presence of a remarkable signal for the C=O group carbon at *δ* 166.7 ppm, a value that indicated that the carbonyl group was adjacent to a sp^3^ nitrogen atom [28] as in the two isomers **17A** and **17C**. The IR of the product **17a** (R = Ph)**,** displayed significant bands at, ύ = 3430 (NH) and 1653 (C=O), which suggested the presence of **17A** tautomer in the solid state, while, the ^1^H-NMR spectrum suggested the existence of **17C** tautomer, through the appearance of a singlet signal for the CH_2_ group at *δ* = 4.44 ppm and lack of from any signals for a NH group (Figure 2), while the other derivative **17b** (R = CH_3_) exists in only the tautomeric form **17A** as proved from its spectral data (see Experimental part). The formation of products **17a**,**b** started with substitution reactions through the formation of intermediate **16**, which was followed by concurrent elimination of a water molecule (Scheme 4).

**Scheme 5:**
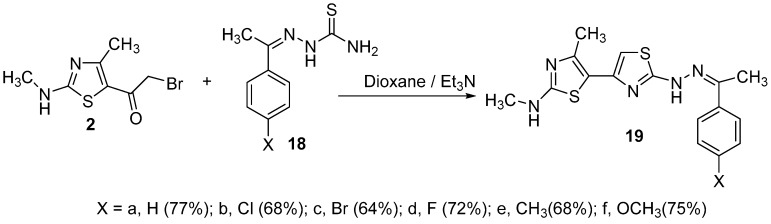
Synthesis of compounds **19a**–**f**.

**Scheme 6:**
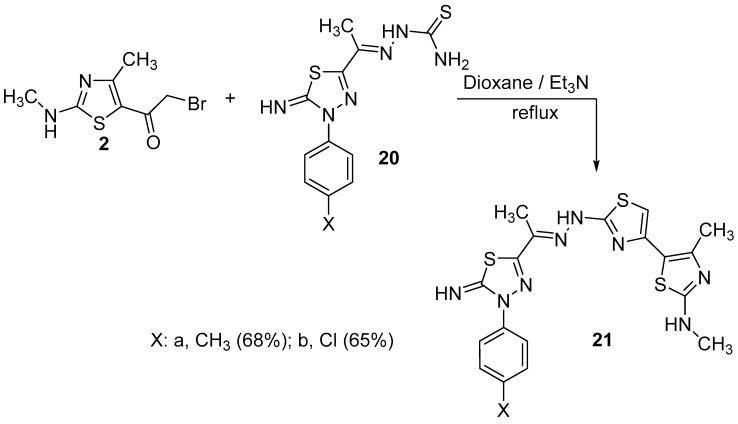
Synthesis of compounds **21a**,**b**.

**Scheme 7:**
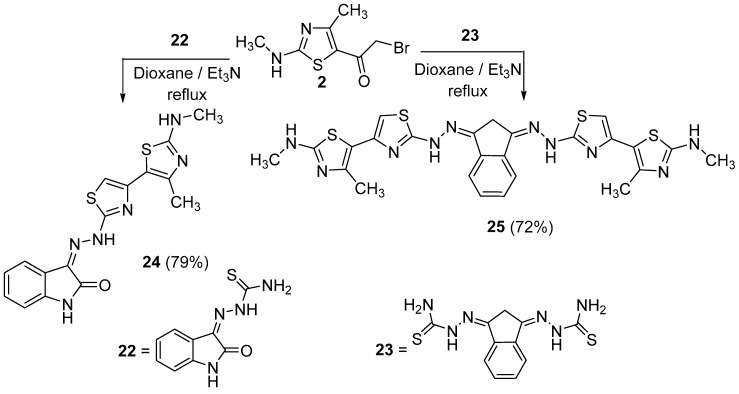
Synthesis of compounds **24** and **25**.

**Figure 3:**
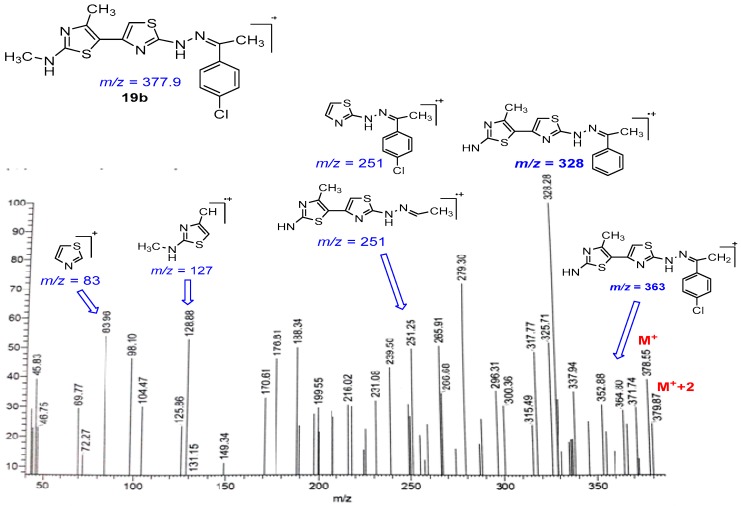
Mass fragmentations for compound **19b**.

The simple and easy synthesis of more than one thiazole ring in molecules was achieved by the reaction of thiasemicarbazone derivatives **18a**–**f**, **20a**,**b**, **22** and **23** with α-bromoketone derivative **2** (Scheme 5, Scheme 6 and Scheme 7). Such reactions were carried out in dioxane in the presence of triethylamine to give thiazole derivatives **19a**–**f**, **21a**,**b**, **24** and **25**. The structure of all these compounds has been verified through their spectral data, elemental analysis and conformational analysis. Figure 3 shows the fragmentation pattern for product **19b**. ^1^H NMR of compound **24** revealed all remarkable signals for aliphatic, aromatic and three NH protons as shown in the Experimental section. Moreover, the data from their IR spectra, which revealed the disappearance of C=O absorption bands and the existence of NH and C=N groups, proved the formation of these compounds **19a**–**f**, **21a**,**b**, **24** and **25**. 

### 2.2. Conformational Study

**Figure 4:**
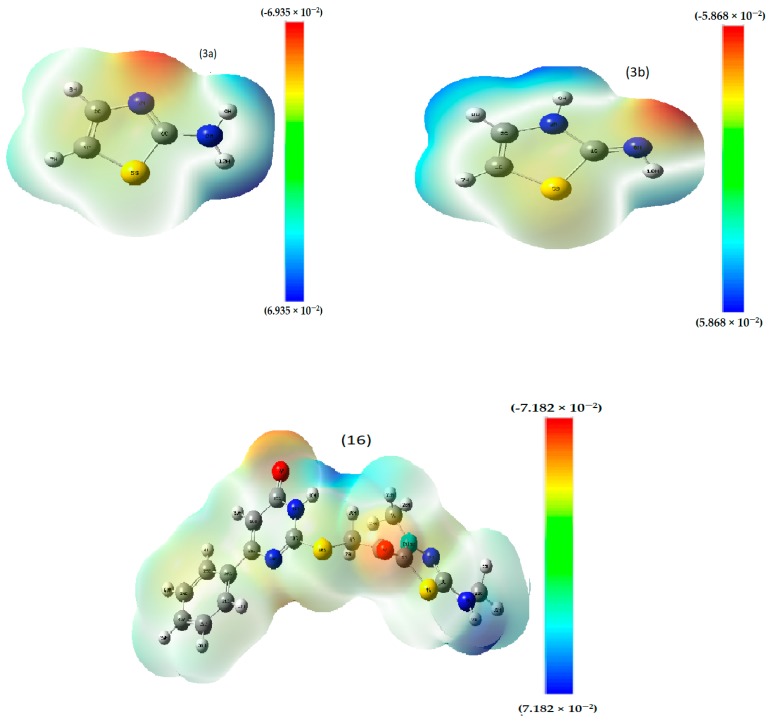
Electrostatic potential of selected reaction determining compounds.

All synthesized conformers as well as tautomers, were structurally investigated (Figure S1), to confirm the proposed reaction mechanism. The reaction mechanism was already established mainly based on spectroscopic tools. The comparative view for conformer couples such as **6A** and **6B**; **7A** and **7B**; **8A** and **8B**; **12A** and **12B**; **17A** and **17B**, may confirm all reactions-pathways. The best discrimination among each couple confirms the mechanisms proposed in all the synthesis schemes. Firstly, their formation energies (Table 1) point to their relative stability, which controls the reaction trends. As we know, the less the energy of formation (E, a. u.), the more stable the conformer. This appeared excellently for tautomers **A** not **B**. Secondly, the recorded energy gaps (∆E = E_LUMO_ − E_HOMO_), reflect the comparative stability that is consistent with the suggested conformers (**A** compounds). With respect to tautomers **3A** and **3B**, we considered the reaction-determining step of Scheme 2, and a remarkable stability was recorded for the **3A** tautomer. Also, regarding the two tautomers, the charge on the N(6) atom was −0.51307 or −0.338355, respectively. The two values indicate the highly nucleophilic nature of the nitrogen atom in compound **3A**. This agrees excellently with the previously suggested reaction pathway. Another additional proof was obtained from their electrostatic maps (Figure 4), which exhibited a broad electron cloud over **3A** (−6.935 × 10^−2^) but not the **3B** tautomer (−5.868 × 10^−2^). Such an observation suggests the superiority of **3A** tautomer over the **3B** one. Regarding intermediate **16**, the one in the reaction pathway in Scheme 4, its electrostatic map displays a broad electron cloud (−7.182 × 10^−2^) over the whole molecule. Also, the charge on N(18) (which is adjacent to the carbonyl group) was −0.782807, while the charge on N (14) was −0.580283, which points to the high nucleophilicity of the N(18) atom, which was already proposed in the reaction mechanism. 

HOMO and LUMO frontier images were extracted and displayed (Figures S2 and S3). The HOMO mainly appeared distributed over the whole molecule in a good way, especially for the **A** conformers, while, the LUMO mainly appeared condensed in a defined part in the compound. This is somewhat logical, that the two orbitals consider the counterparts in the compounds. Essential physical parameters were calculated for all optimized conformers, to gain some insight into their general characteristics. Electronegativity (*χ*), chemical potential (*μ*), global hardness (*η*), global softness (*S*), global electrophilicity index (*ω*) and absolute softness (σ), were the calculated parameters (Table 1). The differentiation between all tested compounds allows us to make the following remarks; (i) the values of electrophilicity index (*ω*), reflect significant toxicity for most of the tested compounds, especially of **19**, **21**, **24** and **25**. (ii) The global hardness (*η*) and absolute softness (σ) values, clarify the high degree of softness for compounds **13**, **19**, **21** and **24** [29,30,31,32], which should led to different biological activity.

### 2.3. Molecular Docking Study

**Figure 5:**
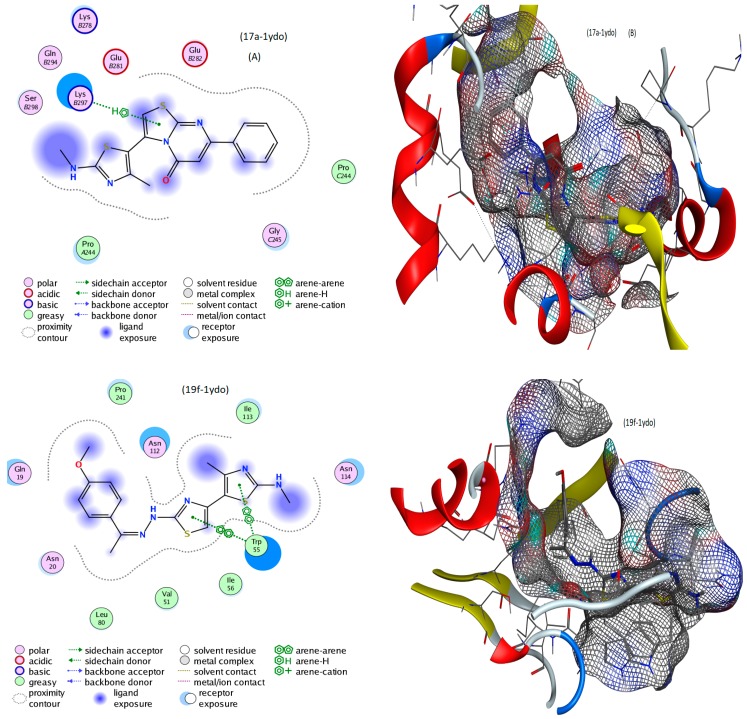

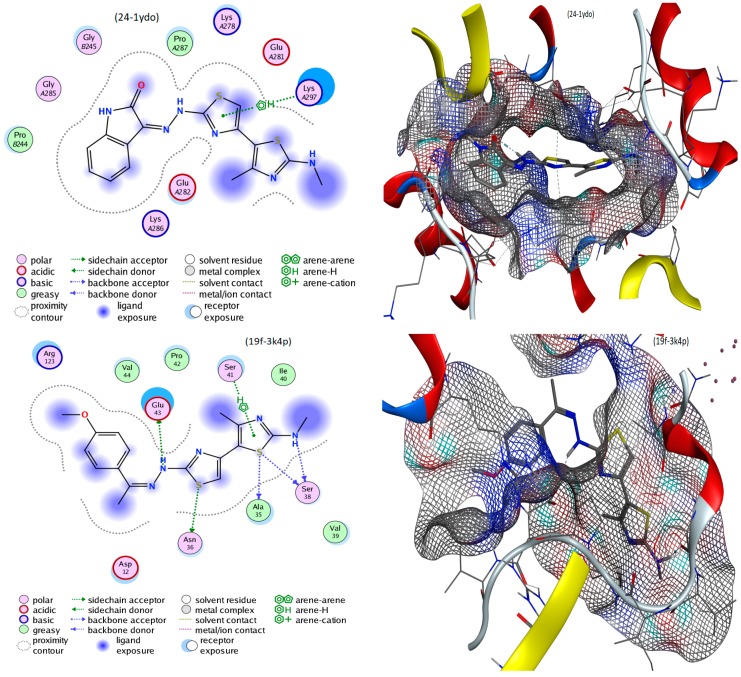
Best docking interaction (**A**) and surfaces maps (**B**) of selected compounds.

This study aimed to provide a good simulation of what happens inside infected cells after treatment with the suggested inhibitors. Implementing the MOE module, the docking process was executed between **14**, **17a**, **19f** and **24** conformers and 3k4p, 1ydo and 1ecl PDB co-crystals of *Aspergillus niger*, *Bacillis subtilis* and *Escherichia coli*, respectively [33]. Fundamental data was exported (Table 2) and docking complexes images (interaction and surface maps), are presented as well in Figure 5 and Figure S4. Table 2 includes ligand types (centers of H- interaction), and protein receptors which interact with the ligands. Types of binding inside complexes, the length of H- bonding as well as energies calculated for docking complexes are also included.

The underlying mechanism of action was suggested by all docking processes, which varied from the best interaction to others with moderate binding, based on the following remarks;. (i) The energy value scores (S) appear in the −5.8156 to −7.0064 and −4.3294 to −5.3916 ranges, which represent the best to moderate interactions, respectively. (ii) The ligation mode was mainly focused on, S, N, 5-ring and 6-ring sites. (iii) The main binding receptor-backbones were lysine, asparagine, glutamate, alanine, serine and tryptophan. (iv) The interactions appeared to covering all possible types, such as π-H, π-π, H-donor and H-acceptor. (v) From all the previous remarks the best inhibition may be expected for **17a**, **24** and **19f** against the 1ydo protein of *Bacillis subtilis* bacteria [34,35]. The displayed images of docking complexes (Figure 5 and Figure S3), especially with the interacting image (A), clarify the formerly concluded data. Regarding the surface maps (B images), which were built over line dummies without isolating the receptor atoms, they lead to a good view of the electrostatic interactions inside the docking complexes. Good inhibition is concluded with some conformers, which exhibit high occupancy of the inside surface grooves that point to a best blocking of amino acid active sites.

### 2.4. Biological Activity

#### Antimicrobial Activity

The antimicrobial activity was assayed for the starting α-bromoketone derivative **2** and the seventeen synthetic derivatives **6**–**8**, **12**–**14**, **17a**,**b**, **19a**, **19c**–**f**, **21a**,**b**, **24**, and **25** against two fungal species, and four Gram-positive as well as four Gram-negative bacteria, as illustrated in Table 3. The activity was reported by measuring the diameter of the inhibition zone (IZD) in mm ± standard deviation. While determining the antimicrobial activity, amphotericin B, ampicillin, and gentamicin were used as reference compounds for fungi, Gram-positive bacteria, and Gram-negative bacteria, respectively.

It is easy to note that the starting α-bromoketone derivative **2** showed less activity than the the used antifungal and antibacterial standards against most of the tested fungi and bacteria. The activity of the thiazole ring was affected by the conversion of the α-bromoketone group to heterocyclic rings at position 5. The presence of an imidazotetrazole ring at position-5 of the thiazole ring, enhanced the activity of compound **12** (IZ = 23.3 µg/mL) to a level similar to that of amphotercin B against *Aspergillus niger*, whereas, upon insertion of imidazotriazole, thiazolopyrimidine, thiazole rings in the same position-5, a favorable activity was recorded for compounds **14**, **17a**, **19a**, **19c**–**f**, **21a** and **24** that exhibited higher activity than the standard drug toward *Aspergillus niger* (Table 4, Table 5 and Table 6). Investigating the product activity results recorded against Gram positive bacteria (*Staphylococcus aureus)*, three derivatives **14**, **19a** and **21a** are more potent than the reference drug (ampicillin), but only derivative **14** exceed the activity of the standard against *Staphylococcus epidermidis.* Observing Gram negative bacteria, six derivatives **17a**,**b**, **19a**,**e**,**f**, **24** are more potent than the reference drug, as depicted in Table 4, Table 5 and Table 6. In contrast to these results, we found that none of the investigated derivatives showed aany activity against Gram-positive *Streptococcus pyogenes* and Gram-negative *Pseudomonas aeruginosa*. 

Table 7 presents the minimum inhibition concentration (MIC) values for some selected examples **7**, **13**, **17a**,**b**, **19c**, and **21b** that showed significant inhibition zones. The results revealed that most of these compounds displayed promising activity against *Aspergillus niger,* whereas, only compound **17a** showed MIC similar to Amphotericin B against *Geotricum candidum*. Focusing on the results of G (^+^) bacteria and G (^−^) bacteria*,* the most active derivative is **17a**. Moreover, compounds **7** and **13** showed high reactivity toward the bacterium *Salmonella typhimurium* (MIC = 0.49 μg/mL). Finally, from the inhibition zone and MIC investigations of the tested compounds, we found that the insertion of a heterocyclic system at position-5 of the thiazole ring, improved the antimicrobial activity. Also, with the presence of more than one thiazole ring in the system, promising activity concluded to be more than that of the reference antibiotics used was observed. 

## 3. Experimental Section

### 3.1. General Experimental Procedures

All melting points were measured on a Electrothermal IA 9000 series digital melting point apparatus (Bibby Scientific Limited. Beacon Road, Stone, Staffordshire, UK). IR spectral analyses were recorded on a FT-IR-4100 spectrophotometer (400–4000 cm^−1^, JASCO, Easton, MD 21601, United States). ^1^H and ^13^C-NMR spectral analysis were obtained on a Bruker 400 MHz instrument (JEOL, Tokyo, Japan). Mass spectral analysis was obtained at 70 eV on Shimadzu GCMS-QP 1000 EX mass spectrometer (Tokyo, Japan) at a heating rate 40 °C/min. The elemental analysis was carried out using a 2400 CHN Elemental Analyzer (Perkin-Elmer, Inc. 940 Winter Street Waltham, MA, USA) instrument. The biological evaluation of the products was carried out at the Regional Center of Mycology and Biotechnology, Al-Azhar University, Cairo, Egypt. Theoretical calculations were performed using Gaussian 09 and AutoDock Tools 4.2. 2-Bromo-1-(4-methyl-2-(methylamino)thiazol-5-yl)ethan-1-one (**2**) was synthesized as previously reported [25].

### 3.2. Reaction of Heterocyclic Amines ***3***, ***4***, ***9***–***11*** and 2-Aminothioplenol (***5***) with 2-Bromo-1-(4-methyl-2-(methylamino)thiazol-5-yl)ethan-1-one (***2***)

A mixture of equimolar quantities of *2-bromo-1-(4-methyl-2-(methylamino)thiazol-5-yl)ethan-1-one* (**2**, 1.25 gm, 5 mmol) and heterocyclic amines **3**, **4**, **9**–**11** or 2-aminothioplenol **5** (5 mmol) in ethanol (30 mL) was refluxed for 10 h (the progress of the reactions was monitored by TLC). After the reaction was complete, the reaction mixture was filtered, the filtrate washed with methanol, and crystallized from a suitable solvent to give derivatives **6**, **7**, **12**–**14** and **8**, respectively. 

#### 3.2.1. 5-(Imidazo[2,1-*b*]thiazol-6-yl)-*N*,4-dimethylthiazol-2-amine (**6**)

Yellow solid, (78% yield), mp 243–244 °C; (EtOH); IR (KBr) ν_max_ 3417 (NH), 3038 (sp^2^ C-H), 2997 (sp^3^ C-H), 1634 (C=N) cm^−1^; ^1^H-NMR (DMSO-*d*_6_) *δ* 2.45 (s, 3H, CH_3_), 3.56 (s, 3H, CH_3_), 7.37 (d, *J* = 3.6 Hz, 1H, thiazole-H), 7.94 (d, *J* = 3.6 Hz, 1H, thiazole-H), 8.20 (s, 1H, imidazole-H), 9.60 (s, 1H, NH); MS *m*/*z* (%) 250 (M^+^, 26), 239 (53), 234 (47), 224 (89), 220 (16), 206 (62), 191 (45), 188 (57), 183 (100), 150 (30), 119 (34), 91 (33), 65 (42), 48 (54). Anal. Calcd. for C_10_H_10_N_4_S_2_ (250.34): C, 47.98; H, 4.03; N, 22.38. Found: C, 48.05; H, 3.89; N, 22.24%.

#### 3.2.2. 5-(Benzo[*d*]imidazo[2,1-*b*]thiazol-2-yl)-*N*,4-dimethylthiazol-2-amine (**7**)

Yellow solid, (83% yield), mp 265–266 °C; (EtOH); IR (KBr) ν_max_ 3238 (NH), 3047 (sp^2^ C-H), 2987 (sp^3^ C-H), 1623 (C=N) cm^−1^; ^1^H-NMR (DMSO-*d*_6_) *δ* 2.59 (s, 3H, CH_3_), 3.56 (s, 3H, CH_3_), 7.40 (t, *J* = 7 Hz, 1H, Ar-H), 7.54 (t, *J* = 7 Hz, 1H, Ar-H), 8.01 (d, *J* = 7.7 Hz, 1H, Ar-H), 8.09 (d, *J* = 7.7 Hz, 1H, Ar-H), 8.76 (s, 1H, imidazole-H), 9.62 (s, 1H, NH); MS *m*/*z* (%) 300 (M^+^, 34), 285 (22), 270 (29), 254 (40), 175 (26), 153 (27), 147 (23), 150 (28), 138 (71), 129 (29), 77 (17). Anal. Calcd. for C_14_H_12_N_4_S_2_ (300.40): C, 55.98; H, 4.03; N, 18.65. Found: C, 55.87; H, 3.87; N, 18.58%.

#### 3.2.3. 5-(4*H*-Benzo[*b*][1,4]thiazin-3-yl)-*N*,4-dimethylthiazol-2-amine (**8**)

Yellow solid, (88% yield), mp 110–112 °C; (EtOH/Dioxane); IR (KBr) ν_max_ 3374, 3293 (2NH), 3059 (sp^2^ C-H), 2926 (sp^3^ C-H), 1612 (C=N) cm^−1^; ^1^H-NMR (DMSO-*d*_6_) *δ* 2.51 (s, 3H, CH_3_), 2.89 (s, 3H, CH_3_), 5.45 (s, 1H, benzothiazine-H), 6.42 (t, *J* = 7 Hz, 1H, Ar-H), 6.73 (d, *J* = 7 Hz, 1H, Ar-H), 7.01 (d, *J* = 7 Hz, 1H, Ar-H), 7.07 (t, *J* = 7 Hz, 1H, Ar-H), 7.4 (s, 1H, NH), 9.07 (s, 1H, NH); MS *m*/*z* (%) 275 (M^+^, 13), 272 (100), 256 (7), 166 (10), 154 (3), 152 (17), 147 (4), 139 (86), 127 (7), 113 (37), 102 (23), 91 (31), 83 (72), 76 (17). Anal. Calcd. for C_13_H_13_N_3_S_2_ (275.39): C, 56.70; H, 4.76; N, 15.26. Found: C, 56.85; H, 4.58; N, 15.17%.

#### 3.2.4. 5-(4*H*-Imidazo[1,2-*d*]tetrazol-5-yl)-*N*,4-dimethylthiazol-2-amine (**12**)

Yellow solid, (76% yield), mp 155–157 °C; (EtOH); IR (KBr) ν_max_ 3428, 3203 (2NH), 3106, 2924 (sp^3^ C-H), 1646, 1599 (C=N) cm^−1^; ^1^H-NMR (DMSO-*d*_6_) *δ* 2.51 (s, 3H, CH_3_), 2.89 (s, 3H, CH_3_), 6.92 (s, 1H, NH), 7.03 (s, 1H, NH), 8.85 (s, 1H, imidazole-H); MS *m*/*z* (%) 235 (M^+^, 25), 220 (16), 210 (55), 192 (24), 191 (33), 189 (38), 170 (23), 123 (32), 106 (24), 103 (26), 96 (34), 82 (20), 64 (100). Anal. Calcd. for C_8_H_9_N_7_S (235.27): C, 40.84; H, 3.86; N, 41.68. Found: C, 41.10; H, 3.65; N, 41.59%.

#### 3.2.5. 5-(1*H*-Benzo[*d*]imidazo[1,2-*a*]imidazol-2-yl)-*N*,4-dimethylthiazol-2-amine (**13**)

Yellow solid, (65% yield), mp 140–142 °C; (EtOH/Dioxane); IR (KBr) ν_max_ 3438, 3165 (2NH), 3094 (sp^2^ C-H), 2991 (sp^3^ C-H), 1649 (C=N) cm^−1^; ^1^H-NMR (DMSO-*d*_6_) *δ* 2.25 (s, 3H, CH_3_), 2.85 (s, 3H, CH_3_), 7.12 (d, *J* = 7 Hz, 1H, Ar-H), 7.24 (d, *J* = 7 Hz, 1H, Ar-H), 8.0–8.21 (m, 2H, Ar-H), 8.29 (s, 1H, imidazole-H), 10.44 (s, 1H, NH), 10.58 (s, 1H, NH); MS *m*/*z* (%) 283 (M^+^, 22), 264 (41), 245 (56), 238 (17), 230 (49), 207 (100), 181 (66), 167 (41), 137 (83), 124 (22), 115 (42), 98 (62), 67 (45). Anal. Calcd. for C_14_H_13_N_5_S (283.35): C, 59.34; H, 4.62; N, 24.72. Found: C, 59.17; H, 4.55; N, 24.60%.

#### 3.2.6. 5-(4*H*-Imidazo[1,2-*b*][1,2,4]triazol-5-yl)-*N*,4-dimethylthiazol-2-amine (**14**)

Yellow crystals, (91% yield), mp 245–146 °C; (EtOH); IR (KBr) ν_max_ 3436, 3124(2NH), 2978 (sp^3^ C-H), 1629 (C=N) cm^−1^; ^1^H-NMR (DMSO-*d*_6_) *δ* 2.41 (s, 3H, CH_3_), 3.03 (s, 3H, CH_3_), 6.75 (s, 1H, NH), 6.85 (s, 1H, NH), 9.55 (s, 2H, 2C-H); MS *m*/*z* (%) 234 (M^+^, 21), 224 (7), 221 (15), 187 (5), 166 (12), 153 (5), 145 (25), 127 (14), 118 (28), 107 (4), 84 (11), 73 (28), 67 (6), 65 (54), 63 (100). Anal. Calcd. for C_9_H_10_N_6_S (234.28): C, 46.14; H, 4.30; N, 35.87. Found: C, 46.25; H, 4.29; N, 35.75%.

### 3.3. Reaction of 2-Bromo-1-(4-methyl-2-(methylamino)thiazol-5-yl)ethan-1-one (***2***) with Pyrimidinethione Derivatives ***15a**,**b** and thiosemicarbazones **18a**–**f**, **20a**,**b 22**, or **23***

To a stirred hot solution of pyrimidinethione derivatives **15a** or **15b** or thiosemicarbazone derivatives **18a**–**f**, **20a**,**b 22**, or **23** (5 mmole) in dioxane (30 mL) was added 2-bromo-1-(4-methyl-2-(methylamino)thiazol-5-yl)ethan-1-one (**2**, 1.25 g, 5 mmol) and triethylamine (0.7 mL) then the reaction mixture was refluxed for 6 h. After cooling, the formed solid product was collected by filtration and washed with aqueous ethanol to dissolve any triethylamine hydrochloride crystals, dried and crystallized from ethanol/dioxane to give thiazolopyrimidines **17a**,**b** or hydrazine-thiazole derivatives **19a**–**f**, **21a**,**b**, **24** and **25**.

#### 3.3.1. 3-(4-Methyl-2-(methylamino)thiazol-5-yl)-7-phenyl-5*H*-thiazolo[3,2-*a*]pyrimidin-5-one (**17a**)

Yellow solid, (81% yield), mp 200–201 °C; IR (KBr) ν_max_ 3430 (NH), 2926 (sp^3^ C-H), 1653 (C=O), 1564 (C=N) cm^−1^; ^1^H-NMR (DMSO-*d*_6_) *δ* 2.51 (s, 3H, CH_3_), 3.30 (s, 3H, CH_3_), 4.44 (s, 2H, CH_2_), 6.69 (s, 1H, pyrimidine-H), 7.36–7.95 (m, 5H, Ar-H); ^13^C-NMR (DMSO-*d*_6_) *δ* 18.9 (CH_3_), 30.1 (CH_2_), 34.0 (N-CH_3_), 110.9, 112.0, 114.3, 119.8, 125.5, 127.32, 129.6, 131.9, 132.6, 145.7, 147.6, 166.7 (C=O) ppm; MS *m*/*z* (%) 354 (M^+^, 40), 338 (28), 331 (40), 327 (30), 311 (41), 269 (44), 258 (69), 201 (30), 173 (43), 155 (56), 111 (83), 86 (47), 60 (100). Anal. Calcd. for C_17_H_14_N_4_OS_2_ (354.45): C, 57.61; H, 3.98; N, 15.81. Found: C, 57.73; H, 3.76; N, 15.72%.

#### 3.3.2. 7-Methyl-3-(4-methyl-2-(methylamino)thiazol-5-yl)-5*H*-thiazolo[3,2-*a*]pyrimidin-5-one (**17b**)

Dark orange solid, (92% yield), mp 259–260 °C; IR (KBr) ν_max_ 3430 (NH), 2928 (sp^3^ C-H), 1636 (C=O), 1560 (C=N) cm^−1^; ^1^H-NMR (DMSO-*d*_6_) *δ* 2.07 (s, 3H, CH_3_), 2.51 (s, 3H, CH_3_), 3.37 (s, 3H, CH_3_), 5.68 (s, 2H, pyrimidine-H and thiazole-H), 12.24 (s, 1H, NH). MS *m*/*z* (%) 292 (M^+^, 100), 284 (59), 277 (23), 265 (21), 256 (63), 246 (32), 231 (25), 202 (44), 163 (25), 139 (16), 127 (40), 108 (53), 87 (35), 82 (25). Anal. Calcd. for C_12_H_12_N_4_OS_2_ (292.38): C, 49.30; H, 4.14; N, 19.16. Found: C, 49.45; H, 4.05; N, 19.02%.

#### 3.3.3. N,4′-dimethyl-2-(2-(1-phenylethylidene)hydrazineyl)-[4,5′-bithiazol]-2′-amine (**19a**)

Green solid, (77% yield), mp 235–237 °C; IR (KBr) ν_max_ br. 3419 (2NH), 2929 (sp^3^ C-H), 1554 (C=N) cm^−1^; ^1^H-NMR (DMSO-*d*_6_) *δ* 2.31 (s, 3H, CH_3_), 2.51 (s, 3H, CH_3_), 2.89 (s, 3H, CH_3_), 7.39–8.27 (m, 7H, Ar-H, thiazole-H, NH), 10.22 (s, 1H, NH); MS *m*/*z* (%) 343 (M^+^, 14), 334 (14), 303 (17), 282 (30), 218 (20), 163 (10), 127 (100), 93 (61), 91 (49), 85 (12). Anal. Calcd. for C_16_H_17_N_5_S_2_ (343.47): C, 55.95; H, 4.99; N, 20.39. Found: C, 56.14; H, 4.80; N, 20.27%.

#### 3.3.4. 2-(2-(1-(4-Chlorophenyl)ethylidene)hydrazineyl)-*N*,4′-dimethyl-[4,5′-bithiazol]-2′-amine (**19b**)

Green solid, (68% yield), mp 226–228 °C; IR (KBr) ν_max_ br. 3423 (2NH), 2925 (sp^3^ C-H),1552 (C=N) cm^−1^; ^1^H-NMR (DMSO-*d*_6_) *δ* 2.29 (s, 3H, CH_3_), 2.33 (s, 3H, CH_3_), 3.37 (s, 3H, CH_3_), 7.42–7.98 (m, 6H, Ar-H, thiazole-H, NH), 10.26 (s, 1H, NH); MS *m*/*z* (%) 379 (M^+^+2, 12), 378 (M^+^+1, 4), 377 (M^+^, 38), 364 (28), 346 (25), 337 (34), 328 (100), 265 (50), 251 (49), 239 (43), 225 (22), 216 (30), 188 (49), 128 (52), 83 (53). Anal. Calcd. for C_16_H_16_ClN_5_S_2_ (377.91): C, 50.85; H, 4.27; N, 18.53. Found: C, 51.02; H, 4.13; N, 18.44%.

#### 3.3.5. 2-(2-(1-(4-Bromophenyl)ethylidene)hydrazineyl)-*N*,4′-dimethyl-[4,5′-bithiazol]-2′-amine (**19c**)

Green solid, (64% yield), mp 220–222 °C; IR (KBr) ν_max_ br. 3416 (2NH), 2919 (sp^3^ C-H), 1552 cm-1; ^1^H-NMR (DMSO-*d*_6_) *δ* 2.28 (s, 3H, CH_3_), 2.33 (s, 3H, CH_3_), 3.36 (s, 3H, CH_3_), 7.56–7.92 (m, 5H, Ar-H, thiazole-H), 8.31 (s, 1H, NH), 10.26 (s, 1H, NH); MS *m*/*z* (%) 422 (M^+^, 25), 406 (52), 390 (100), 371 (56), 347 (32), 325 (37), 302 (38), 270 (67), 238 (36), 111 (24), 72 (20). Anal. Calcd. for C_16_H_16_BrN_5_S_2_ (422.36): C, 45.50; H, 3.82; N, 16.58. Found: C, 45.75; H, 3.68; N, 16.46%.

#### 3.3.6. 2-(2-(1-(4-Fluorophenyl)ethylidene)hydrazineyl)-*N*,4′-dimethyl-[4,5′-bithiazol]-2′-amine (**19d**)

Green solid, (72% yield), mp 249–250 °C; IR (KBr) ν_max_ 3423, 3249 (2NH), 3161, 2936 (sp^3^ C-H), 1602, 1560 (C=N) cm^−1^; ^1^H-NMR (DMSO-*d*_6_) *δ* 2.28 (s, 3H, CH_3_), 2.51 (s, 3H, CH_3_), 3.51 (s, 3H, CH_3_), 7.17–8.00 (m, 5H, Ar-H, thiazole-H), 8.24 (s, 1H, NH), 10.19 (s, 1H, NH); MS *m*/*z* (%) 361 (M^+^, 12), 338 (22), 299 (66), 273 (45), 271 (100), 252 (16), 202 (16), 189 (15), 136 (39). Anal. Calcd. for C_16_H_16_FN_5_S_2_ (361.46): C, 53.17; H, 4.46; N, 19.38. Found: C, 53.24; H, 4.31; N, 19.29%.

#### 3.3.7. *N*,4′-Dimethyl-2-(2-(1-(*p*-tolyl)ethylidene)hydrazineyl)-[4,5′-bithiazol]-2′-amine (**19e**)

Green solid, (68% yield), mp 229–230 °C; IR (KBr) ν_max_ br. 3411 (2NH), 2919 (sp^3^ C-H), 1552 cm^−1^; ^1^H-NMR (DMSO-*d*_6_) *δ* 2.25 (s, 3H, CH_3_), 2.35 (s, 3H, CH_3_), 2.50 (s, 3H, CH_3_), 3.26 (s, 3H, CH_3_), 6.94 (d, *J* = 7 Hz, 2H, Ar-H), 7.07 (s, 1H, thiazole-H), 7.76 (d, *J* = 7 Hz, 2H, Ar-H), 8.50 (s, 1H, NH), 10.21 (s, 1H, NH); MS *m*/*z* (%) 357 (M^+^, 35), 346 (53), 324 (100), 298 (60), 270 (45), 229 (34), 174 (68), 145 (44), 135 (81), 115 (50), 65 (56). Anal. Calcd. for C_17_H_19_N_5_S_2_ (357.49): C, 57.12; H, 5.36; N, 19.59. Found: C, 57.30; H, 5.33; N, 19.46%.

#### 3.3.8. 2-(2-(1-(4-Methoxyphenyl)ethylidene)hydrazineyl)-*N*,4′-dimethyl-[4,5′-bithiazol]-2′-amine (**19f**)

Green solid, (75% yield), mp 195–197 °C; IR (KBr) ν_max_ 3386, 3265 (2NH), 2926 (sp^3^ C-H), 1597 (C=N) cm^−1^; ^1^H-NMR (DMSO-*d*_6_) *δ* 2.00 (s, 3H, CH_3_), 2.51 (s, 3H, CH_3_), 2.80 (s, 3H, CH_3_), 3.86 (s, 3H, OCH_3_), 7.30 (s, 1H, thiazole-H), 7.44 (d, *J* = 7 Hz, 2H, Ar-H), 7.57 (d, *J* = 7 Hz, 2H, Ar-H), 8.29 (s, 1H, NH), 10.39 (s, 1H, NH); MS *m*/*z* (%) 373 (M^+^, 24), 369 (52), 346 (100), 332 (55), 284 (24), 270 (63), 250 (34), 177 (36), 123 (37), 111 (63), 76 (38). Anal. Calcd. for C_17_H_19_N_5_OS_2_ (373.49): C, 54.67; H, 5.13; N, 18.75. Found: C, 54.76; H, 5.24; N, 18.65%.

#### 3.3.9. 2-(2-(1-(5-Imino-4-(*p*-tolyl)-4,5-dihydro-1,3,4-thiadiazol-2-yl)ethylidene)hydrazineyl)-N,4′-dimethyl-[4,5′-bithiazol]-2′-amine (**21a**)

Green solid, (68% yield), mp 270–271 °C; IR (KBr) ν_max_ br. 3423 (3NH), 2927 (sp^3^ C-H), 1590 (C=N) cm^−1^; ^1^H-NMR (DMSO-*d*_6_) *δ* 2.34 (s, 3H, CH_3_), 2.39 (s, 3H, CH_3_), 2.51 (s, 3H, CH_3_), 3.35 (s, 3H, CH_3_), 6.69–8.08 (m, 6H, Ar-H, thiazole-H, NH), 9.39 (s, 1H, NH), 10.91 (s, 1H, NH); MS *m*/*z* (%) 456 (M^+^, 34), 420 (24), 367 (24), 267 (26), 249 (52), 200 (44), 139 (33), 104 (21), 85 (60), 76 (29). Anal. Calcd. for C_19_H_20_N_8_S_3_ (456.61): C, 49.98; H, 4.42; N, 24.54. Found: C, 50.11; H, 4.27; N, 24.35%.

#### 3.3.10. 2-(2-(1-(4-(4-Chlorophenyl)-5-imino-4,5-dihydro-1,3,4-thiadiazol-2-yl)ethylidene)-hydrazineyl)-*N*,4′-dimethyl-[4,5′-bithiazol]-2′-amine (**21b**)

Green solid, (65% yield), mp 278–280 °C; IR (KBr) ν_max_ br. 3425 (3NH), 2991 (sp^3^ C-H), 1621 (C=N) cm^−1^; ^1^H-NMR (DMSO-*d*_6_) *δ* 1.92 (s, 3H, CH_3_), 2.41 (s, 3H, CH_3_), 2.68 (s, 3H, CH_3_), 7.50–8.11 (m, 6H, Ar-H, thiazole-H, NH), 8.66 (s, 1H, NH), 10.92 (s, 1H, NH); MS *m*/*z* (%) 479 (M^+^+2, 34), 477 (M^+^, 73), 461 (41), 382 (33), 313 (15), 222 (26), 202 (36), 111 (15), 108 (32), 96 (100). Anal. Calcd. for C_18_H_17_ClN_8_S_3_ (477.02): C, 45.32; H, 3.59; N, 23.49. Found: C, 45.43; H, 3.41; N, 23.28%.

#### 3.3.11. 3-(2-(4′-Methyl-2′-(methylamino)-[4,5′-bithiazol]-2-yl)hydrazineylidene)indolin-2-one (**24**)

Brown solid, (79% yield), mp 210–212 °C; IR (KBr) ν_max_ 3419, 3262 (3NH), 1650 (C=O), 1600 (C=N) cm^−1^; ^1^H-NMR (DMSO-*d*_6_) *δ* 2.51 (s, 3H, CH_3_), 3.39 (s, 3H, CH_3_), 6.92 (d, *J* = 7.8 Hz, 1H, Ar-H), 7.07 (t, *J* = 7.45 Hz, 1H, Ar-H), 7.33 (t, *J* = 7.5 Hz, 1H, Ar-H), 7.65 (d, *J* = 7.44 Hz, 1H, Ar-H), 8.68 (s, 1H, thiazole-H), 9.03 (s, 1H, NH), 11.21 (s, 1H, NH), 12.48 (s, 1H. NH); MS *m*/*z* (%) 370 (M^+^, 29), 356 (50), 340 (32), 244 (45), 337 (50), 305 (40), 297 (63), 282 (25), 264 (58), 224 (32), 211 (44), 190 (100), 87 (44), 82 (19), 77 (32). Anal. Calcd. for C_16_H_14_N_6_OS_2_ (370.45): C, 51.88; H, 3.81; N, 22.69. Found: C, 52.02; H, 3.65; N, 22.53%.

#### 3.3.12. 2,2′′-((1*H*-Indene-1,3(2*H*)-diylidene)bis(hydrazin-1-yl-2-ylidene))bis(*N*,4′-dimethyl-[4,5′-bithiazol]-2′-amine) (**25**)

Brown solid, (72% yield), mp 216–217 °C; IR (KBr) ν_max_ 3415, 3240 (2NH), 2937 (sp^3^ C-H), 1575 (C=N) cm^−1^; ^1^H-NMR (DMSO-*d*_6_) *δ* 2.51 (s, 6H, 2CH_3_), 3.38 (s, 6H, 2CH_3_), 3.83 (s, 2H, CH_2_), 7.51–8.03 (m, 4H, Ar-H), 8.13(s, 2H, thiazole-H), 8.40 (s, 2H, 2NH), 10.02 (s, 2H, 2NH); MS *m*/*z* (%) 593 (M^+^+1, 11), 567 (14), 548 (23), 531 (20), 510 (14), 439 (20), 404 (20), 360 (100), 352 (15), 276 (31), 226 (11), 211 (13), 200 (58). Anal. Calcd. for C_25_H_24_N_10_S_4_ (592.78): C, 50.66; H, 4.08; N, 23.63. Found: C, 50.76; H, 4.14; N, 23.58%.

### 3.4. Antimicrobial Screening

The starting α-bromoketone derivative **2** and the new thiazole compounds **6**–**8**, **12**–**14**, **17a**,**b**, **19a**, **19c**–**f**, **21a**,**b**, **24** and **25** were assayed in vitro for their antimicrobial activity against two fungal species, namely *Aspergillus niger, Geotricum candidum*, four Gram-positive bacteria*: Staphylococcus aureus, Staphylococcus epidermidis, Bacillis subtilis, Streptococcus pyogenes,* and four Gram-negative bacteria: *Pseudomonas aeruginosa, Escherichia coli, Klebsiella pneumoniae, Salmonella typhimurium* using the known the agar diffusion procedure [36]. Firstly, the microorganisms were screened separately versus the activity of the solutions of each compound (30 μg/mL) and using the IZD diameter of the mm/mg sample as a measure of antimicrobial activity. The microorganisms were spread uniformly using a sterile cotton swab on a fresh Petri dish filled with agar and nutritient agar. One hundred μL of each sample was added to each well (10 mm diameter holes cut into the agar gel, 20 mm apart from each other). Petri dishes were incubated at 36 °C for bacteria and at 28 °C for fungus and inhibition zones were measured after 24 h of incubation. Inhibition of growth of bacteria and fungi was measured as IZD in mm. Each test was repeated three times and the average value is recorded. The fungicide amphotericin and gentamicin and ampicillin were used as a reference to evaluate the efficacy of the tested compounds under the same conditions.

### 3.5. Conformational Study

The synthesis pathways were also confirmed based on structural configuration studies which were performed using the Gaussian 09 software [37]. To determine the most stable conformers (in gas phase), the DFT method was applied as B3LYP-FC, by using the 3-21G or 6-31G basis sets. Two significant files were extracted (log and chk), which feed the study of the essential parameters, for confirmation. All optimized conformers were visualized using the Gauss-View program screen [38], to obtain frontier energy gaps and electrostatic potential maps. Other physical parameters were computed by using standard equations [39,40].

### 3.6. Molecular Docking Study

Through the MOE module (v. 2015), the molecular docking process was implemented forselected compounds against three microorganisms. Regarding the tested inhibitors, the selection aimd to cover all the various synthesis pathways, to give a broad view about their interactions with pathogen proteins. Regarding the selected pathogens, *Aspergillus niger*, *Bacillis subtilis* and *Escherichia coli*, which cover fungi and bacteria, were selected, which will simulate the intended screening in vitro study. 3k4p, 1ydo and 1ecl, were the co-crystalline structures as PDB files, which are attributed to the reported microorganisms, respectively. This introductory study may strengthen the experimental screening, which will be displayed later. Firstly the docked synthesis must be configured by the addition of hydrogen atoms, after removing water molecules around the skeletons. Then the potential energy must be fixed after displaying the atomic charges and other important parameters adapted by the MMFF94x force field. After that, the oriented compounds must be saved as MDB format, to be ready for the docking process. The PDB files of co-crystallized proteins are configured by addition of hydrogen, choosing the receptor and generating the α-site spheres in the site finder [41]. The docking inhibitors will attack the protein-surfaces in its interior-grooves, after 30 trials, till the most stable docking complexes are reached. The scoring energies, which were the mean values of trials using the London dG scoring function, were upgraded by two unrelated-refinements by the triangular Matcher methods. The interacting complexes as well as electrostatic maps for the interacting surfaces were extracted, in addition to essential interaction parameters. The grade of inhibition was judged based on the extracted parameters such as ligand, receptor backbones (amino acids), interaction type, bond lengths, internal and scoring energies. The bond lengths must be verified, are were limited to ≤ 3.5 Å

## 4. Conclusions

A main and growing health problem around the world is microbial resistance to existing treatments. Several studies have been conducted aiming to synthesize thiazole derivatives with excellent antimicrobial activity. In this context we focused on the synthesis for a new series of thiazole derivatives with varying substituents at position-5. The atomic assembly and the mechanistic pathway for all products have been investigated and proved based on our spectral and conformational studies. Antimicrobial results for the starting material (α-bromoketone) and the products indicated that the antimicrobial activity of some new synthetic products is more efficient than that of the starting material itself. This activity of the derivatives was somewhat improved to exceed the activity of standard drugs.

## Figures and Tables

**Table 1:** Calculated physical parameters for all suggested conformers.

Conformers	E_H_ (eV)	E_L_ (eV)	E_H_ − E_L_	E_L_ − E_H_	*x*(eV)	µ(eV)	η(eV)	S(eV-1)	ω(eV)	σ(eV)	D_(Debye)_	E _(a. u.)_
**3A**	−0.20464	−0.00446	−0.2002	0.20018	0.10455	−0.10455	0.10009	0.050045	0.054604	9.991008093	2.4276	−621.17858
**3B**	−0.20794	−0.01183	−0.1961	0.19611	------	------	------	------	------	------	2.7935	−621.14667
**6A**	−0.2024	−0.07871	−0.1237	0.12369	0.140555	−0.14056	0.061845	0.030923	0.15972	16.1694559	6.0085	−1394.9439
**6B**	−0.17951	−0.07617	−0.1033	0.10334	------	------	------	------	------	------	10.9130	−1394.4678
**7A**	−0.19955	−0.0716	−0.128	0.12795	0.135575	−0.13558	0.063975	0.031988	0.143654	15.6311059	6.0625	−1547.7641
**7B**	−0.14724	−0.09765	−0.0496	0.04959	------	------	------	------	------	------	13.5781	−1547.1272
**8A**	−0.1861	−0.06861	−0.1175	0.11749	0.127355	−0.12736	0.058745	0.029373	0.138048	17.02272534	7.8499	−1456.1575
**8B**	−0.18661	−0.0561	−0.1305	0.13051	------	------	------	------	------	------	6.2621	−1456.0837
**12A**	−0.20807	−0.08663	−0.1214	0.12144	0.14735	−0.14735	0.06072	0.03036	0.178788	16.46903821	12.6129	−1085.6105
**12B**	−0.21277	−0.10635	−0.1064	0.10642	------	------	------	------	------	------	10.4034	−1085.4218
**13**	−0.19029	−0.09108	−0.0992	0.09921	0.140685	−0.14069	0.049605	0.024803	0.199499	20.15925814	10.1242	−1206.5564
**14**	−0.2007	−0.06744	−0.1333	0.13326	0.13407	−0.13407	0.06663	0.033315	0.134885	15.00825454	8.2866	−1069.7172
**16**	−0.21735	−0.06425	−0.1531	0.1531	0.1408	−0.1408	0.07655	0.038275	0.129488	13.06335728	5.5798	−1813.6492
**17A (R = Ph)**	−0.19633	−0.06873	−0.1276	0.1276	0.13253	−0.13253	0.0638	0.0319	0.13765	15.67398119	3.9127	−1737.5001
**17B (R = Ph)**	−0.16956	−0.10827	−0.0613	0.06129	------	------	------	------	------	------	15.6710	−1728.4984
**19a**	−0.22389	−0.12297	−0.1009	0.10092	0.17343	−0.17343	0.05046	0.02523	0.298038	19.81767737	2.9358	−1679.9270
**21 (X = H)**	−0.21284	−0.12465	−0.0882	0.08819	0.168745	−0.16875	0.044095	0.022048	0.322881	22.6783082	6.1375	−2315.6659
**24**	−0.17775	−0.09774	−0.08	0.08001	0.137745	−0.13775	0.040005	0.020003	0.237141	24.99687539	2.8473	−1817.4536
**25**	−0.25151	−0.01744	−0.2341	0.23407	0.134475	−0.13448	0.117035	0.058518	0.077257	8.544452514	6.9055	−3093.4323

**Table 2:** The energy score and binding data for all compounds against selected proteins.

Compound	Protein	Ligand	Receptor		Interaction	Distance	E (Kcal/mol)	S (Energy Score)
**14**	3k4p	S 4	OG	SER	H-donor	3.75	−0.5	−5.1153
			99	(A)				
		N 13	OG	SER	H-donor	3.09	−1.6	
			99	(A)				
		5-ring	CB	THR	pi-H	4.41	−0.5	
			248	(A)				
	1ydo	-----	-----		-----	-----	-----	−4.5481
	1ecl	N 13	O	HOH	H-donor	3.33	−1.7	−4.3294
			744	(A)				
**17a**	3k4p	S 4	OE1	GLN	H-donor	3.18	−0.7	−5.2078
			13	(B)				
		6-ring	CB	GLN	pi-H	4.00	−0.5	
			16	(B)				
		6-ring	CA	CYS	pi-H	4.35	−0.8	
			17	(B)				
	1ydo	5-ring	CB	LYS	pi-H	4.00	−1.1	−7.0064
			297	(B)				
	1ecl	-----	-----		-----	-----	-----	−4.9748
**19f**	3k4p	S 1	OD1	ASN	H-donor	3.88	−1.4	−5.8156
			36	(A)				
		N 6	OE2	GLU	H-donor	2.99	−4.6	
			43	(A)				
		S 20	O	ALA	H-donor	3.43	−1.3	
			35	(A)				
		N 22	O	SER	H-donor	3.01	−1.7	
			38	(A)				
		5-ring	CA	SER	pi-H	4.10	−0.5	
			41	(A)				
	1ydo	5-ring	5-ring	TRP	pi-pi	3.88	−0.0	−6.2008
			55	(A)				
		5-ring	6-ring	TRP	pi-pi	4.00	−0.0	
			55	(A)				
		5-ring	6-ring	TRP	pi-pi	3.8	−0.0	
			55	(A)				
	1ecl	S 20	OD2	ASP	H-donor	3.52	−2.9	−5.0501
			62	(A)				
		N 7	O	HOH	H-acceptor	3.03	−0.9	
			1030	(A)				
		N 18	O	HOH	H-acceptor	3.60	−1.2	
			1039	(A)				
**24**	3k4p	N 7	OE1	GLU	H-donor	3.2	−0.7	−5.3916
			37	(A)				
		N 11	O	GLU	H-donor	3.49	−0.7	
			37	(A)				
		S 17	O	GLU	H-donor	4.21	−0.9	
			37	(A)				
		5-ring	CG	PRO	pi-H	3.64	−0.8	
			409	(A)				
	1ydo	5-ring	CB	LYS	pi-H	4.27	−0.5	−6.8467
			297	(B)				
	1ecl	S 22	OD2	ASP	H-donor	3.80	−3.3	−4.9736
			506	(A)				
		N 23	OD2	ASP	H-donor	3.05	−4.0	
			506	(A)				
		O 13	NZ	LYS	H-acceptor	3.22	−5.1	
			302	(A)				
		5-ring	O	HOH	pi-H	3.63	−1.2	
			661	(A)				

**Table 3:** Preliminary anti-microbial activity for compounds **2**, **6**–**8**, **12**.

Microorganism	2	6	7	8	12	ST. (30 µg/mL)
***Fungi***						**Amphotericin B**
***Aspergillus niger***	18.8 ± 0.63	19.3 ± 0.63	18.4 ± 1.20	19.2 ± 0.72	23.3 ± 0.72	23.3 ± 0.58
***Geotricum candidum***	19.3 ± 0.58	18.1 ± 0.58	16.3 ± 0.63	17.3 ± 0.72	22.3 ± 1.20	25.2 ± 0.72
***G(^+^) Bacteria***						**Ampicillin**
***Staphylococcus aureus***	17.6 ± 0.85	19.3 ± 0.58	21.1 ± 0.63	13.5 ± 1.20	20.1 ± 0.58	23.7 ± 0.63
***Staphylococcus epidermidis***	18.1 ± 1.20	17.4 ± 0.58	20.7 ± 0.72	15.3 ± 0.44	17.6 ± 0.72	22.4 ± 0.3
***Bacillis subtilis***	18.3 ± 0.63	22.7 ± 1.20	20.1 ± 0.53	18.2 ± 0.58	24.3 ± 0.58	32.4 ± 1.20
***Streptococcus pyogenes***	NA	NA	NA	NA	NA	24.5 ± 0.63
***G(^−^) Bacteria***						**Gentamicin**
***Pseudomonas aeruginosa***	NA	NA	NA	NA	NA	22.3 ± 0.58
***Escherichia coli***	20.2 ± 0.58	20.3 ± 0.63	19.3 ± 1.20	19.3 ± 0.63	23.7 ± 0.72	25.4 ± 1.20
***Klebsiella pneumoniae***	18.2 ± 1.20	18.2 ± 0.58	19.2 ± 1.20	16.3 ± 0.63	19.3 ± 0.63	22.6 ± 0.63
***Salmonella typhimurium***	17.5 ± 0.63	20.4 ± 0.72	18.4 ± 0.58	19.3 ± 0.58	21.6 ± 0.63	23.3 ± 0.58

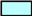
 The tested compound has activity similar to reference drug.

**Table 4:** Preliminary anti-microbial activity for compounds **13**, **14**, **17a**,**b** and **19a**.

Microorganism	13	14	17a	17b	19a	ST. (30 µg/mL)
***Fungi***						**Amphotericin B**
***Aspergillus niger***	21.2 ± 1.20	25.4 ± 1.20	24.3 ± 1.20	23.1 ± 1.20	25.0 ± 0.72	23.3 ± 0.58
***Geotricum candidum***	19.3 ± 0.85	20.9 ± 0.72	24.2 ± 1.20	23.6 ± 1.20	23.6 ± 0.63	25.2 ± 0.72
***G (^+^)Bacteria***						**Ampicillin**
***Staphylococcus aureus***	16.8 ± 0.63	24.3 ± 0.63	23.6 ± 0.63	22.4 ± 0.63	23.8 ± 0.72	23.7 ± 0.63
***Staphylococcus epidermidis***	15.2 ± 0.58	22.8 ± 0.58	22.4 ± 0.72	20.9 ± 0.72	21.9 ± 0.72	22.4 ± 0.3
***Bacillis subtilis***	20.6 ± 0.58	21.6 ± 0.53	27.2 ± 0.63	29.8 ± 0.63	25.2 ± 0.63	32.4 ± 1.20
***Streptococcus pyogenes***	NA	NA	NA	NA	NA	24.5 ± 0.63
***G (^−^)Bacteria***						**Gentamicin**
***Pseudomonas aeruginosa***	NA	NA	NA	NA	NA	22.3 ± 0.58
***Escherichia coli***	19.8 ± 1.20	24.3 ± 1.20	26.7 ± 1.20	24.3 ± 1.20	26.9 ± 1.20	25.4 ± 1.20
***Klebsiella pneumoniae***	18.3 ± 0.53	22.2 ± 0.44	25.3 ± 1.20	22.2 ± 1.20	26.3 ± 1.20	22.6 ± 0.63
***Salmonella typhimurium***	20.1 ± 0.63	21.1 ± 0.72	26.3 ± 0.58	24.8 ± 0.58	22.2 ± 0.58	23.3 ± 0.58

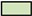
 The tested compound has activity exceed the reference drug.

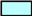
 The tested compound has activity similar to reference drug.

**Table 5:** Preliminary anti-microbial activity for compounds **19c**–**f** and **21a**.

Microorganism	19c	19d	19e	19f	21a	ST. (30 µg/mL)
***Fungi***						**Amphotericin B**
***Aspergillus niger***	21.8 ± 0.43	25.2 ± 2.10	24.1 ± 0.58	26.3 ± 0.63	25.2 ± 2.10	23.3 ± 0.58
***Geotricum candidum***	21.4 ± 0.72	23.2 ± 2.10	20.5 ± 0.58	24.2 ± 0.63	23.2 ± 2.10	25.2 ± 0.72
***G (^+^) Bacteria***						**Ampicillin**
***Staphylococcus aureus***	20.3 ± 0.43	22.1 ± 1.20	22.4 ± 0.63	22.7 ± 1.20	24.1 ± 1.20	23.7 ± 0.63
***Staphylococcus epidermidis***	18.6 ± 0.58	19.8 ± 0.67	20.9 ± 0.72	21.3 ± 0.58	19.8 ± 0.67	22.4 ± 0.3
***Bacillis subtilis***	22.4 ± 1.53	24.3 ± 0.58	23.8 ± 0.63	23.9 ± 0.72	26.3 ± 0.58	32.4 ± 1.20
***Streptococcus pyogenes***	NA	NA	NA	NA	NA	24.5 ± 0.63
***G (^−^) Bacteria***						**Gentamicin**
***Pseudomonas aeruginosa***	NA	NA	NA	NA	NA	22.3 ± 0.58
***Escherichia coli***	23.3 ± 0.58	24.2 ± 0.58	24.3 ± 1.20	25.4 ± 1.20	24.2 ± 0.58	25.4 ± 1.20
***Klebsiella pneumoniae***	20.4 ± 0.53	21.3 ± 0.58	22.5 ± 1.20	23.1 ± 1.20	21.3 ± 0.58	22.6 ± 0.63
***Salmonella typhimurium***	22.6 ± 0.63	23.3 ± 1.20	26.3 ± 0.58	26.7 ± 0.72	23.3 ± 1.20	23.3 ± 0.58

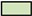
 The tested compound has activity exceed the reference drug.

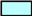
 The tested compound has activity similar to reference drug.

**Table 6:** Preliminary anti-microbial activity for compounds **21b**, **24** and **25**.

Microorganism	21b	24	25	ST. (30 µg/mL)
***Fungi***				**Amphotericin B**
***Aspergillus niger***	19.2 ± 0.72	26.3 ± 0.63	23.3 ± 0.72	23.3 ± 0.58
***Geotricum candidum***	16.3 ± 0.72	24.2 ± 0.63	21.4 ± 0.72	25.2 ± 0.72
***G(^+^) Bacteria***				**Ampicillin**
***Staphylococcus aureus***	17.5 ± 1.20	22.7 ± 1.20	20.3 ± 0.43	23.7 ± 0.63
***Staphylococcus epidermidis***	21.3 ± 0.44	21.3 ± 0.58	19.8 ± 0.58	22.4 ± 0.3
***Bacillis subtilis***	18.2 ± 0.58	28.9 ± 0.72	25.4 ± 0.53	32.4 ± 1.20
***Streptococcus pyogenes***	NA	NA	NA	24.5 ± 0.63
***G (^−^) Bacteria***				**Gentamicin**
***Pseudomonas aeruginosa***	NA	NA	NA	22.3 ± 0.58
***Escherichia coli***	20.3 ± 0.63	25.6 ± 1.20	24.3 ± 0.53	25.4 ± 1.20
***Klebsiella pneumoniae***	21.3 ± 0.63	23.8 ± 1.20	21.5 ± 0.53	22.6 ± 0.63
***Salmonella typhimurium***	19.9 ± 0.58	25.4 ± 0.72	22.6 ± 0.63	23.3 ± 0.58

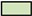
 The tested compound has activity exceed the reference drug.

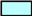
 The tested compound has activity similar to reference drug.

**Table 7:** Minimum inhibitory concentration of compounds **7**, **13**, **17a**,**b**, **19c** and **21b** (µg/mL).

Microorganism	7	13	17a	17b	19c	21b	ST. (30 µg/mL)
***Fungi***							**Amphotericin B**
***Aspergillus niger***	0.98	3.9	0.98	0.98	0.98	31.25	0.98
***Geotricum candidum***	0.98	1.95	0.49	0.98	3.9	62.5	0.49
***G (^+^) Bacteria***							**Ampicillin**
***Staphylococcus aureus***	1.95	31.25	0.98	0.98	3.9	62.5	0.98
***Staphylococcus epidermidis***	3.9	31.25	1.95	7.81	7.81	1.95	1.95
***Bacillis subtilis***	0.98	62.5	3.9	0.49	31.25	62.5	0.49
***Streptococcus pyogenes***	NA	NA	NA	NA	NA	NA	0.49
***G (^−^) Bacteria***							**Gentamicin**
***Pseudomonas aeruginosa***	NA	NA	NA	NA	NA	NA	1.95
***Escherichia coli***	0.98	7.81	0.49	0.49	1.95	7.81	0.49
***Klebsiella pneumoniae***	1.95	3.9	0.98	1.95	0.98	3.9	0.98
***Salmonella typhimurium***	0.49	0.49	1.95	0.98	1.95	3.9	0.98

## Supplementary Materials

The following are available online.

## References

[1] Shirai A., Fumoto Y., Shouno T., Maseda H., Omasa T. (2013). Synthesis and biological activity of thiazolyl-acetic acid derivatives as possible antimicrobial agents. Biocontrol Sci..

[2] Prajapati N.P., Patel K.D., Vekariya R.H., Patel H.D., Rajani D.P. (2019). Thiazole fused thiosemicarbazones: Microwave-assisted synthesis, biological evaluation and molecular docking study. J. Mol. Str..

[3] Lino C.I., de Souza I.G., Borelli B.M., Matos T.T.S., Teixeira I.N.S., Ramos J.P., de Souza Fagundes E.M., Fernandes P.O., Maltarollo V.G., Johann S. (2018). Synthesis, molecular modeling studies and evaluation of antifungal activity of a novel series of thiazole derivatives. Eur. J. Med. Chem..

[4] Reddy G.M., Garcia J.R., Reddy V.H., de Andrade A.M., Camilo A., Ribeiro R.A.P., de Lazaro S.R. (2016). Synthesis, antimicrobial activity and advances in structure-activity relationships (SARs) of novel tri-substituted thiazole derivatives. Eur. J. Med. Chem..

[5] Sinha S., Doble M., Manju S.L. (2018). Design, synthesis and identification of novel substituted 2-amino thiazole analogues as potential anti-inflammatory agents targeting 5-lipoxygenase. Eur. J. Med. Chem..

[6] Kamble R.D., Meshram R.J., Hese S.V., More R.A., Kamble S.S., Gacche R.N., Dawane B.S. (2016). Synthesis and in silico investigation of thiazoles bearing pyrazoles derivatives as anti-inflammatory agents. Comput. Biol. Chem..

[7] Pember S.O., Mejia G.L., Price T.J., Pasteris R.J. (2016). Piperidinyl thiazole isoxazolines: A new series of highly potent, slowly reversible FAAH inhibitors with analgesic properties. Bioorg. Med. Chem. Lett..

[8] Wang Y., Wu C., Zhang Q., Shan Y., Gu W., Wang S. (2019). Design, synthesis and biological evaluation of novel *β*-pinene-based thiazole derivatives as potential anticancer agents via mitochondrial-mediated apoptosis pathway. Bioorg. Chem..

[9] Santana T.I., Barbosa M.O., Gomes P.A.T.M., Cruz A.C.N., Silva T.G., LimaLeite A.C. (2018). Synthesis, anticancer activity and mechanism of action of new thiazole derivatives. Eur. J. Med. Chem..

[10] Cushman M.S., Seleem M., Mayhoub A.S. (2017). Antimicrobial Substituted Thiazoles and Methods of Use. United States Patent.

[11] Leoni A., Locatelli A., Morigi R., Rambaldi M. (2014). Novel thiazole derivatives: A patent review (2008–2012; Part 1). Expert Opin. Ther. Patents.

[12] Muhammad Z.A., Masaret G.S., Amin M.M., Abdallah M.A., Farghaly T.A. (2017). Anti-inflammatory, analgesic and anti-ulcerogenic activities of novel bis-thiadiazoles, bis-thiazoles and bis-formazanes. Med. Chem..

[13] Gong C., Gao A.-H., Zhang Y.-M., Su M.-B., Chen F., Sheng L., Zhou Y.-B., Li J.-Y., Li J., Nan F.-J. (2016). Design, synthesis and biological evaluation of bisthiazole-based trifluoromethyl ketone derivatives as potent HDAC inhibitors with improved cellular efficacy. Eur. J. Med. Chem..

[14] Fairhurst R.A., Imbach-Weese P., Gerspacher M., Caravatti G., Furet P., Zoller T., Christine F., Haasen D., Trappe J., Guthy D.A. (2015). Identification and optimisation of a 4′,5-bisthiazole series of selective phosphatidylinositol-3 kinase alpha inhibitors. Bioorg. Med. Chem. Lett..

[15] Althagafi I.I., Abouzied A.S., Farghaly T.A., Al-Qurashi N.T., Alfaifi M.Y., Shaaban M.R., Abdel Azizd M.R. (2019). Novel Nano-sized bis-indoline Derivatives as Antitumor Agents. J. Heterocyclic Chem..

[16] Turan-Zitouni G., Altıntop M.D., Özdemir A., Kaplancıklı Z.A., Çiftçi G.A., Temel H.E. (2016). Synthesis and evaluation of bis-thiazole derivatives as new anticancer agents. Eur. J. Med. Chem..

[17] Álvarez G., Martínez J., Varela J., Birriel E., Cruces E., Gabay M., Leal S.M., Escobar P., Aguirre-López B., Cabrera N. (2015). Development of bis-thiazoles as inhibitors of triosephosphate isomerase from *Trypanosoma cruzi*. Identification of new non-mutagenic agents that are active in vivo Panel. Eur. J. Med. Chem..

[18] Alsharekh M.M., Althagafi I.I., Shaaban M.R., Farghaly T.A. (2019). Microwave assisted and thermal synthesis of nanosized thiazolyl-phenothiazine derivatives and their biological activities. Res. Chem. Intermed..

[19] Althagafi I., El-Metwally N.M., Elghalban M., Farghaly T.A., Khedr A.M. (2018). Synthesis of Pyrazolone Derivatives and their Nanometer Ag(I)complexes, Physicochemical, DNA binding, Anti-Tumor and Theoretical Implementations. Bioinorg. Chem. Appl..

[20] Dawood D.H., Abbas E.M.H., Farghaly T.A., Ali M.M., Ibrahim M.F. (2019). ZnO Nanoparticles catalyst in Synthesis of Bioactive Fused Pyrimidines as Anti-breast Cancer Agents Targeting VEGFR-2. Med. Chem..

[21] Farghaly T.A., Abdel Hafez N.A., Ragab E.A., Awad H.M., Abdalla M.M. (2010). Synthesis, anti-HCV, antioxidant, and peroxynitrite inhibitory activity of fused benzosuberone derivatives. Eur. J. Med. Chem..

[22] Farghaly T.A., Abdallah M.A., Masaret G.S., Muhammad Z.A. (2015). New and efficient approach for synthesis of novel bioactive [1,3,4]thiadiazoles incorporated with 1,3-thiazole moiety. Eur. J. Med. Chem..

[23] Riyadh S.M., Farghaly T.A., Abdallah M.A., Abdalla M.M., Abd El-Aziz M.R. (2010). New pyrazoles incorporating pyrazolylpyrazole moiety: Synthesis, anti-HCV and antitumor activity. Eur. J. Med. Chem..

[24] Al-Fahemi J.H., Saad F.A., El-Metwaly N.M., Farghaly T.A., Elghalban M.G., Saleh K.A., Al-Hazmi G.A. (2017). Synthesis of Co(II), Cu(II), Hg(II), UO2(II) and Pb(II) binuclear nanometric complexes from multi-donor ligand: Spectral, modeling, quantitative structure – activity relationship, docking and antitumor studies. Appl Organometal Chem..

[25] Mohamed S.F., Abbas E.M., Khalaf H.H.S., Farghaly T.A., Abd El-Shafy D.N. (2018). Triazolopyrimidines and Thiazolopyrimidines: Synthesis, Anti-HSV-1, Cytotoxicity and Mechanism of action. Med. Chem..

[26] Hiebel M.-A., Fall Y., Scherrmann M.-C., Berteina-Raboin S. (2014). Straightforward Synthesis of Various 2,3-Diarylimidazo[1,2-a]pyridines in PEG400 Medium through One-Pot Condensation and C–H Arylation. Eur. J. Org. Chem..

[27] Ramprasad J., Nayak N., Dalimba U., Yogeeswari P., Sriram D., Peethambar S.K., Achur R., Kumar H.S.S. (2015). Synthesis and biological evaluation of new imidazo[2,1-b][1,3,4] thiadiazole-benzimidazole derivatives. Eur. J. Med. Chem..

[28] Shawali A.S., Abdalla M.A., Mosselhi M.A.N., Farghaly T.A. (2002). A Facile one-pot regioselective synthesis of [1,2,4]triazolo[4,3-a]-5(1H)-pyrimidinones via tandem Japp-Klingemann, Smiles rearrangement, and cyclization reactions. Heteroatom Chem..

[29] Chikate R.C., Padhye S.B. (2005). Transition metal quinone–thiosemicarbazone complexes 2: Magnetism, ESR and redox behavior of iron (II), iron (III), cobalt (II) and copper (II) complexes of 2-thiosemicarbazido-1,4-naphthoquinone. Polyhedron.

[30] Sagdinc S., Köksoy B., Andemirli K.F., Bayari S.H. (2009). Theoretical and spectroscopic studies of 5-fluoro-isatin-3-(N-benzylthiosemicarbazone) and its zinc(II) complex. J. Mol. Struct..

[31] Fleming I. (1976). Frontier Orbital’s and Organic Chemical Reactions.

[32] Tripathi S.K., Muttineni R., Singh S.K. (2013). Extra precision docking, free energy calculation and molecular dynamics simulation studies of CDK2 inhibitors. J. Theor. Biol..

[33] Gross S., Rahal R., Stransky N., Lengauer C., Hoeflich K.P. (2015). Targeting cancer with kinase inhibitors. J. Clin. Investig..

[34] Fadda A.A., Abdel-Latif E., Fekri A., Mostafa A.R. (2019). Synthesis and Docking Studies of Some 1,2,3-Benzotriazine-4-one Derivatives as Potential Anticancer Agents. J. Heterocyclic Chem..

[35] Abd El-Karima S.S., Anwara M.M., Syama Y.M., Naelb M.A., Alia H.F., Motaleb M.A. (2018). Rational design and synthesis of new tetralin-sulfonamide derivatives as potent anti-diabetics and DPP-4 inhibitors: 2D & 3D QSAR, in vivo radiolabeling and bio distribution studies. Bioorg. Chem..

[36] Cruickshank R., Duguid J.P., Marion B.P., Swain R.H.A. (1975). Medicinal Microbiology.

[37] Frisch M.J., Trucks G.W., Schlegel H.B., Scuseria G.E., Robb M.A., Cheeseman J.R., Scalmani G., Barone V., Petersson G.A., Nakatsuji H. (2010). Gaussian 09.

[38] Dennington R., Keith T., Millam J. (2007). Gauss View, Version 4.1.2.

[39] El-Ayaan U., El-Metwally N.M., Youssef M.M., El Bialy S.A.A. (2007). Perchlorate mixed–ligand copper(II) complexes of β-diketone and ethylene diamine derivatives: Thermal, spectroscopic and biochemical studies. Spectrochim. Acta Part A.

[40] Ray R.K., Kauffman G.R. (1990). EPR Spectra and covalency of bis(amidinourea/O-alkyl-1-amidinourea)copper(II) complexes Part II.Properties of the CuN42− chromophore. Inorg. Chem. Acta.

[41] Mashat K.H., Babgi B.A., Hussien M.A. (2019). Synthesis, structures, DNA-binding and anticancer activities of some copper(I)-phosphine complexes. Polyhedron.

