# Supplementary material for: New Series of Thiazole Derivatives: Synthesis, Structural Elucidation, Antimicrobial Activity, Molecular Modeling and MOE Docking
Source: Molecules. 2019 May 4;24(9):1741. doi: 10.3390/molecules24091741 (PMC6539608; doi:10.3390/molecules24091741)

(3a)

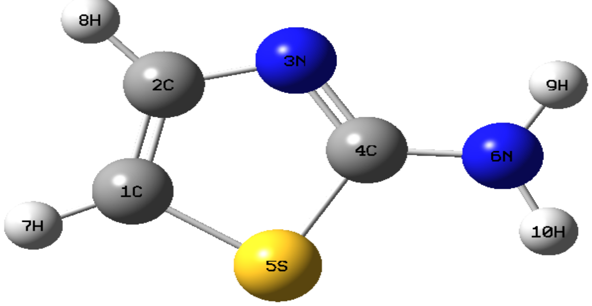

(3b)

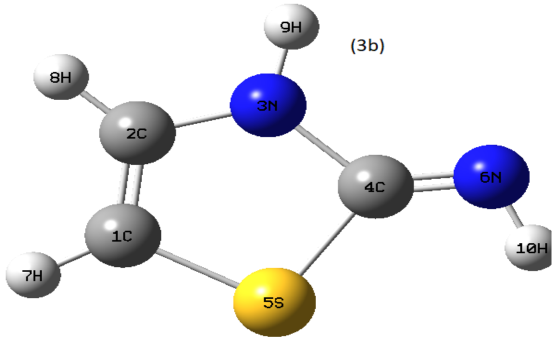

(6a)

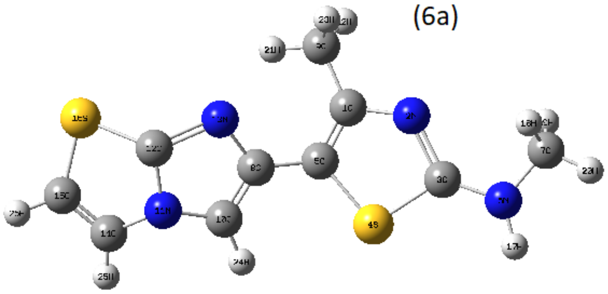

(6b)

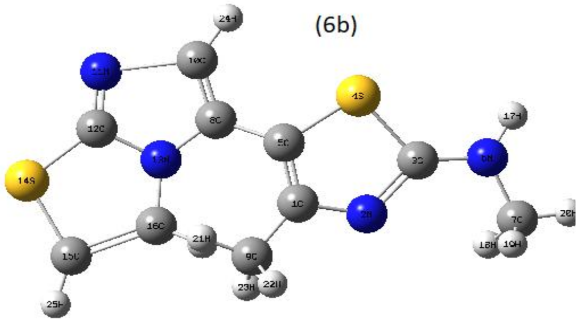

(7a)

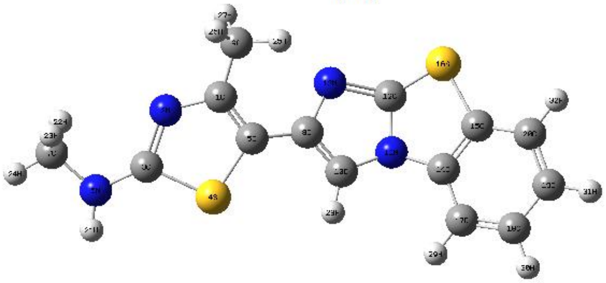

(7b)

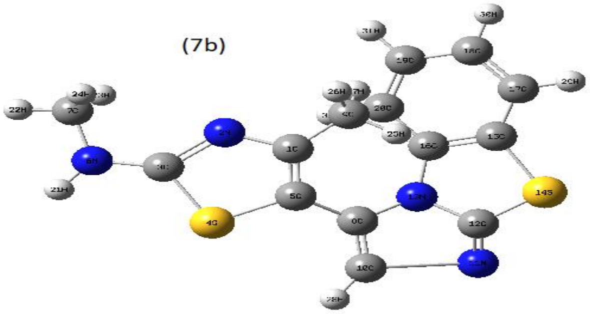

(8a)

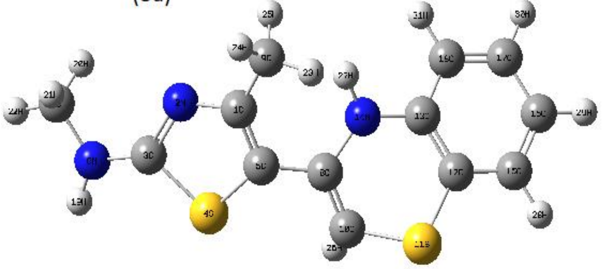

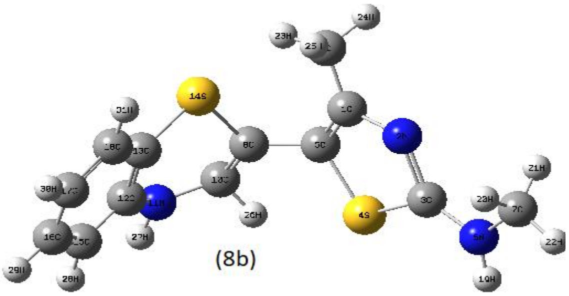

(8b)

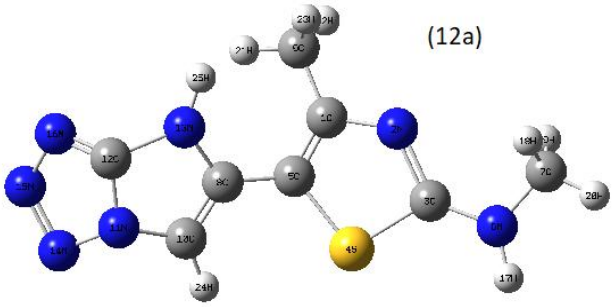

(12a)

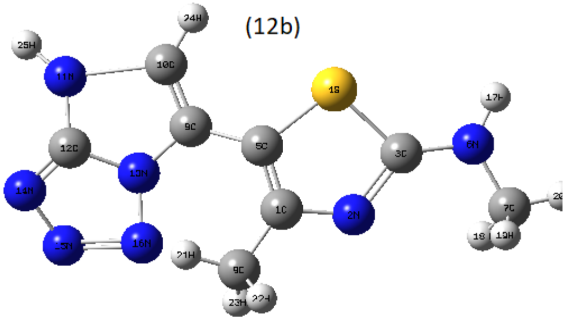

(12b)

(13)

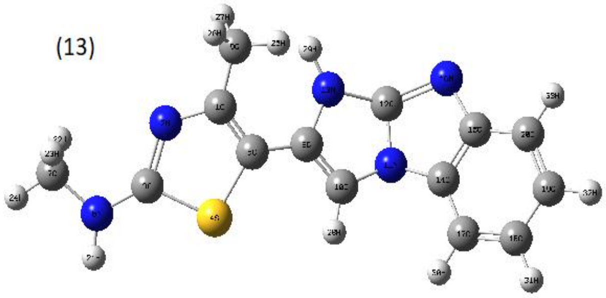

(14)

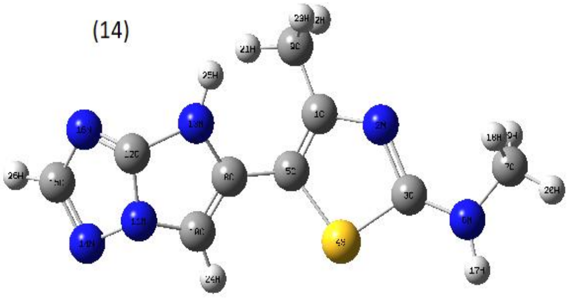

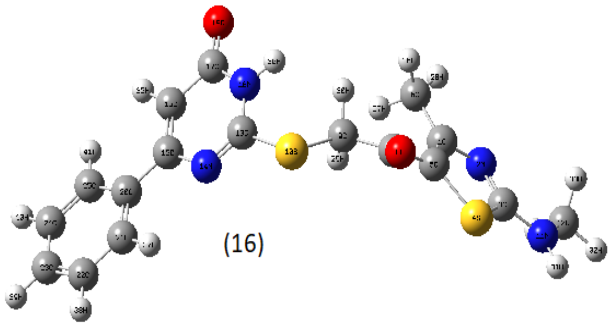

(16)

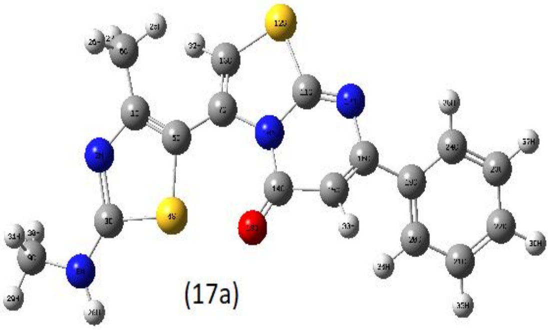

(17a)

(17b)

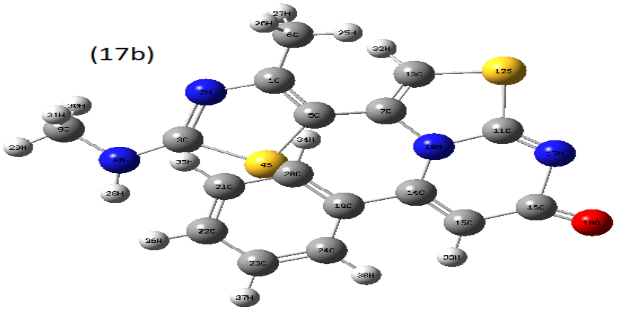

(19)

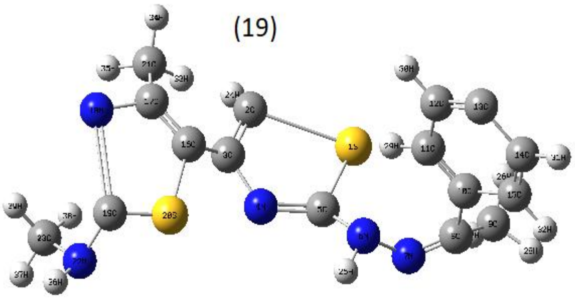

(21)

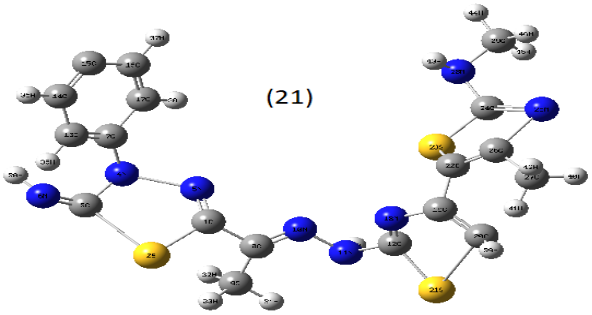

(24)

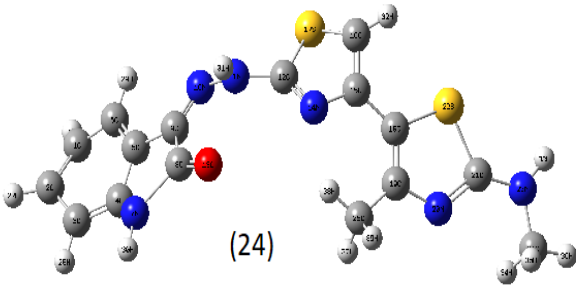

(25)

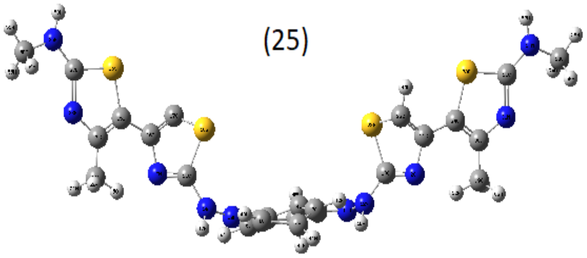

Fig. 1S. Optimized structures for synthesized compounds

(3a)

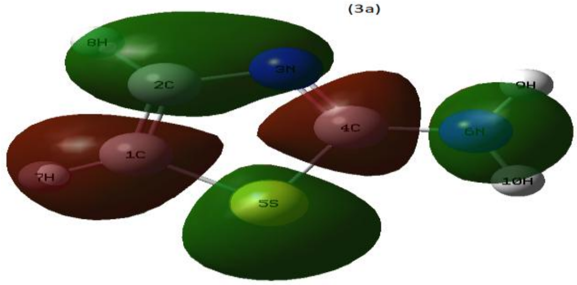

(3b)

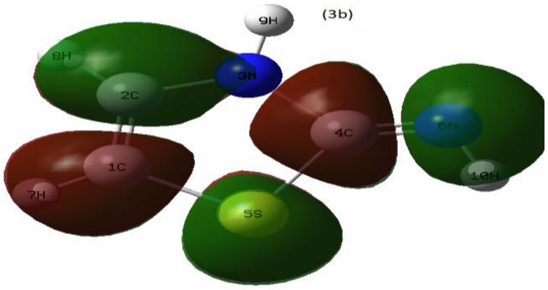

(6a)

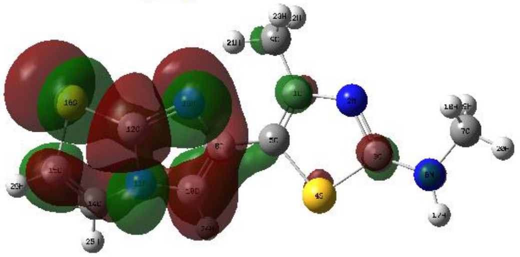

(6b)

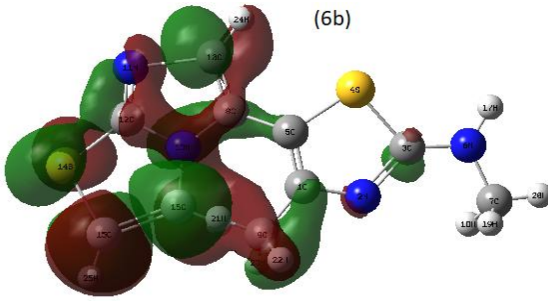

(7a)

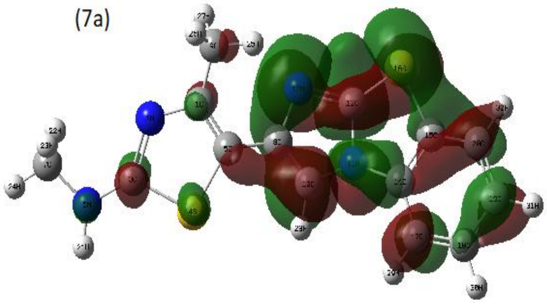

(7b)

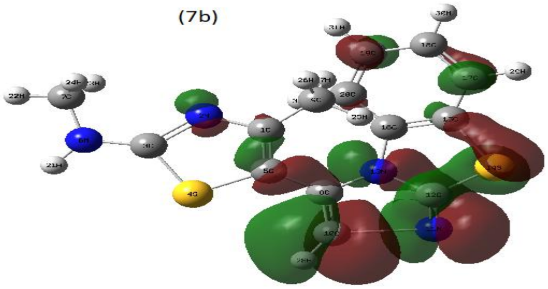

(8a)

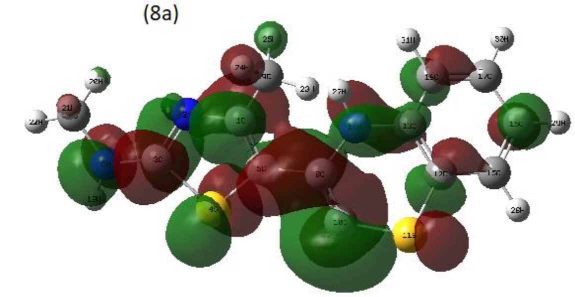

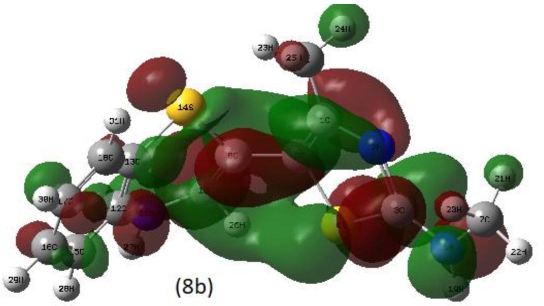

(8b)

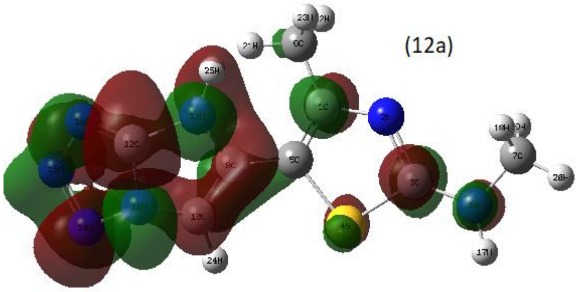

(12a)

(12b)

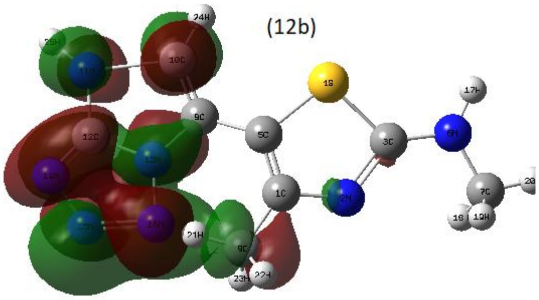

(13)

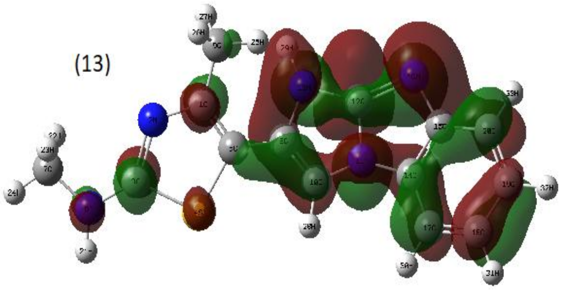

(14)

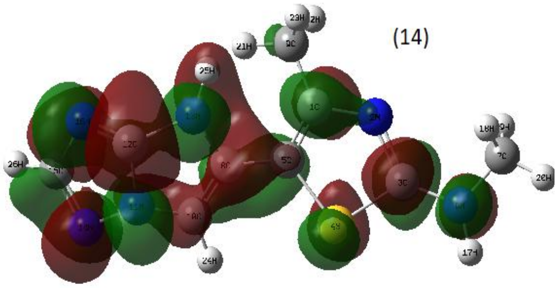

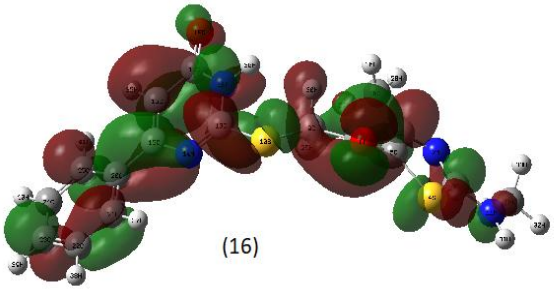

(16)

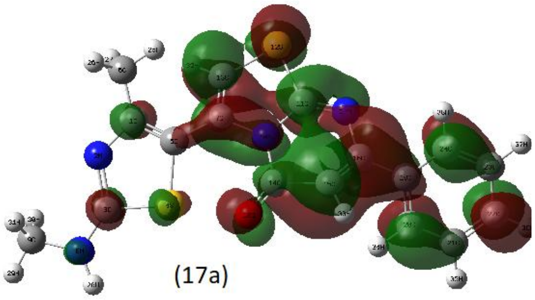

(17a)

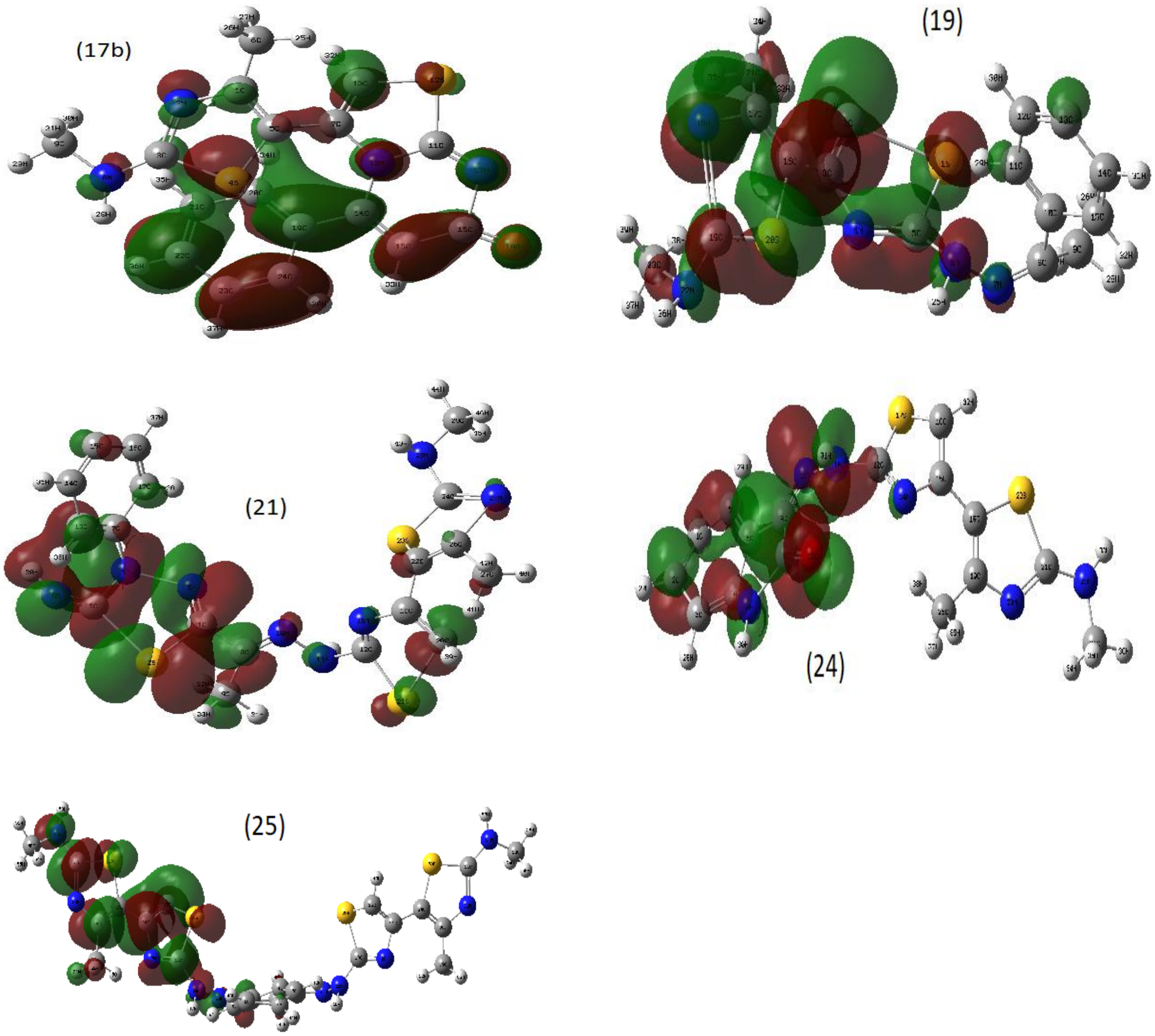

Fig. 2S. LUMO images of optimized structures

(3a)

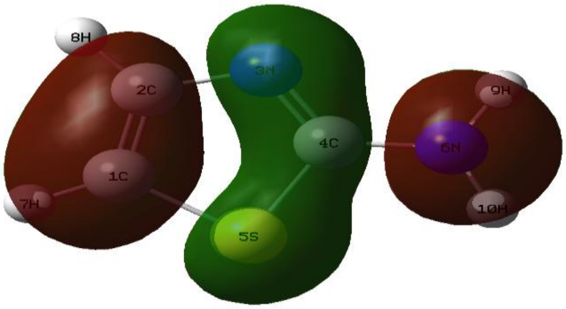

(3b)

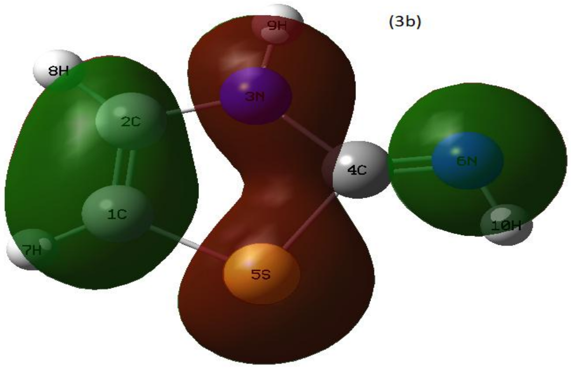

(6a)

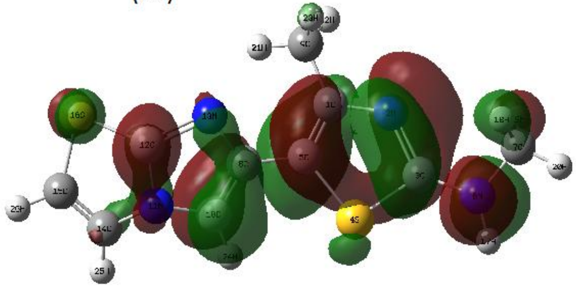

(6b)

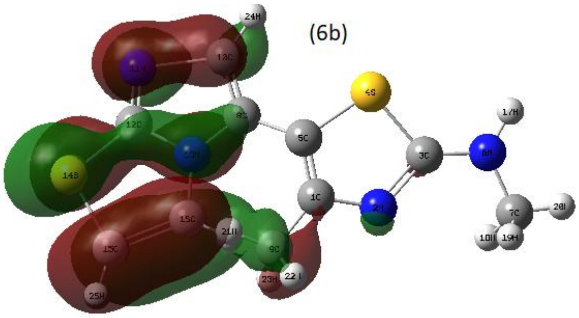

(7a)

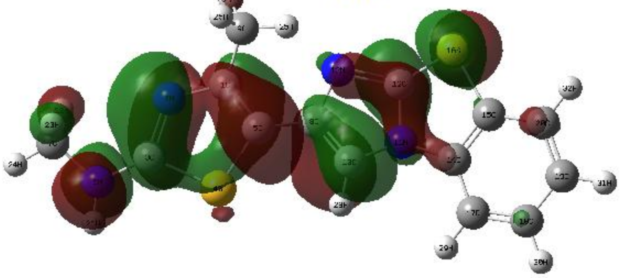

(7b)

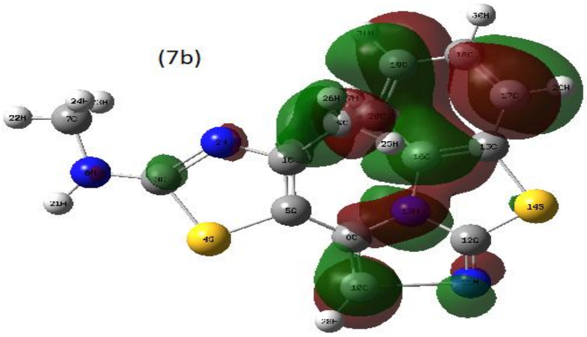

(8a)

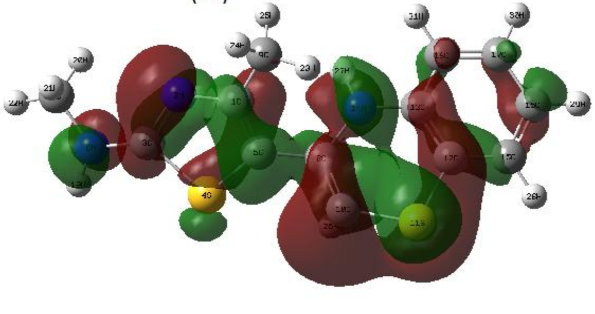

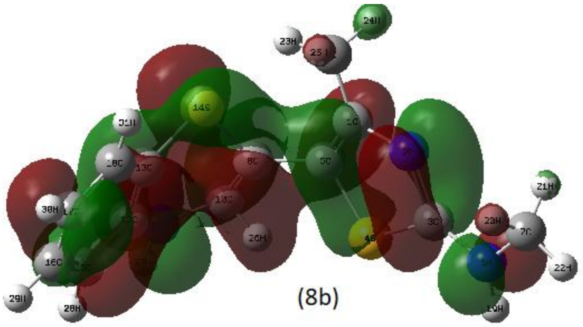

(8b)

(12a)

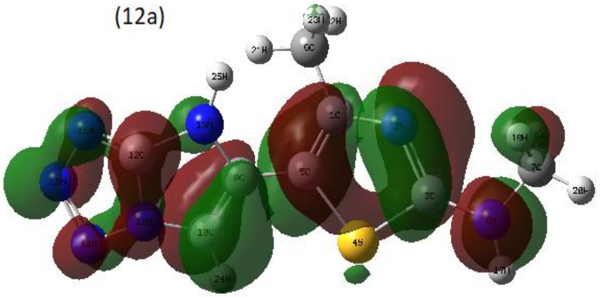

(12b)

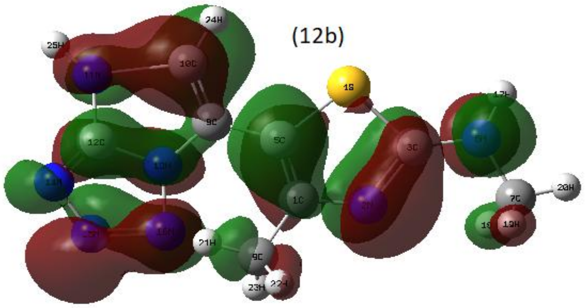

(13)

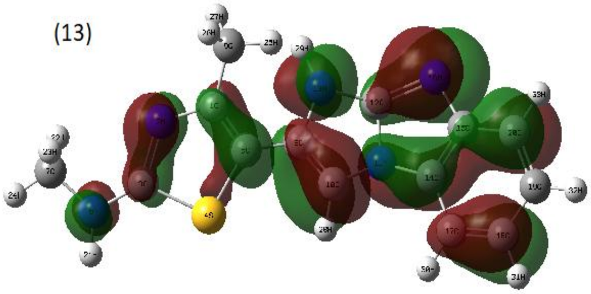

(14)

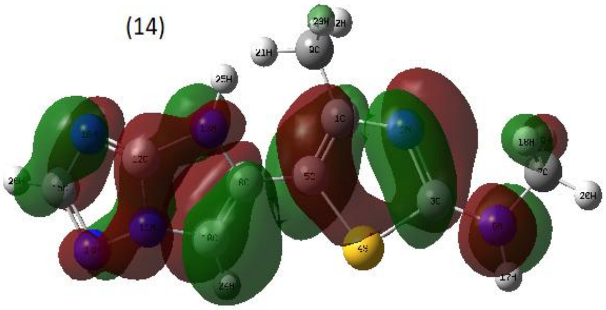

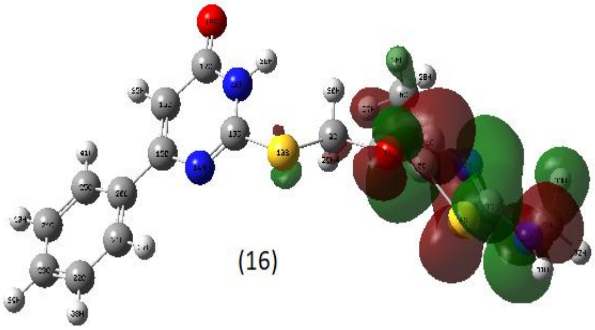

(16)

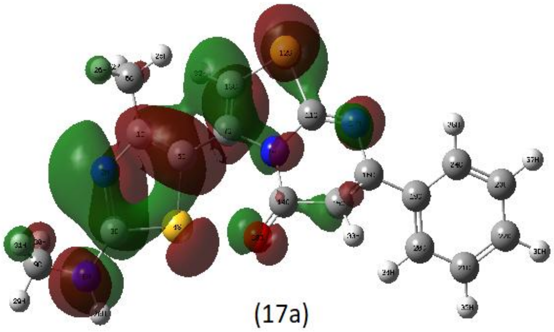

(17a)

(17b)

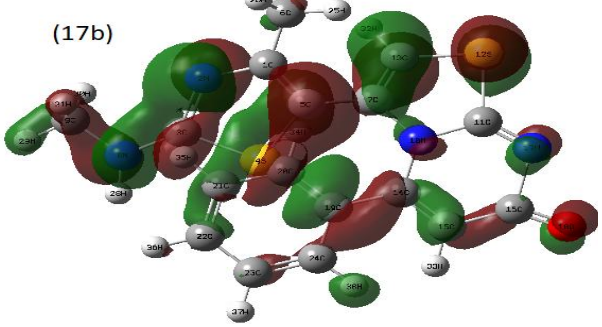

(19)

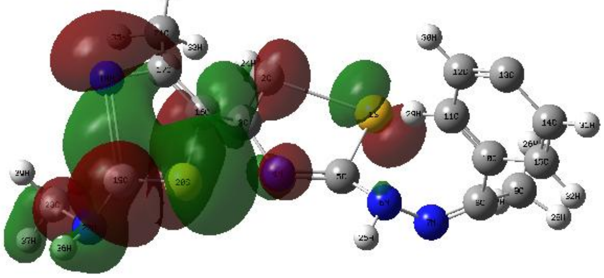

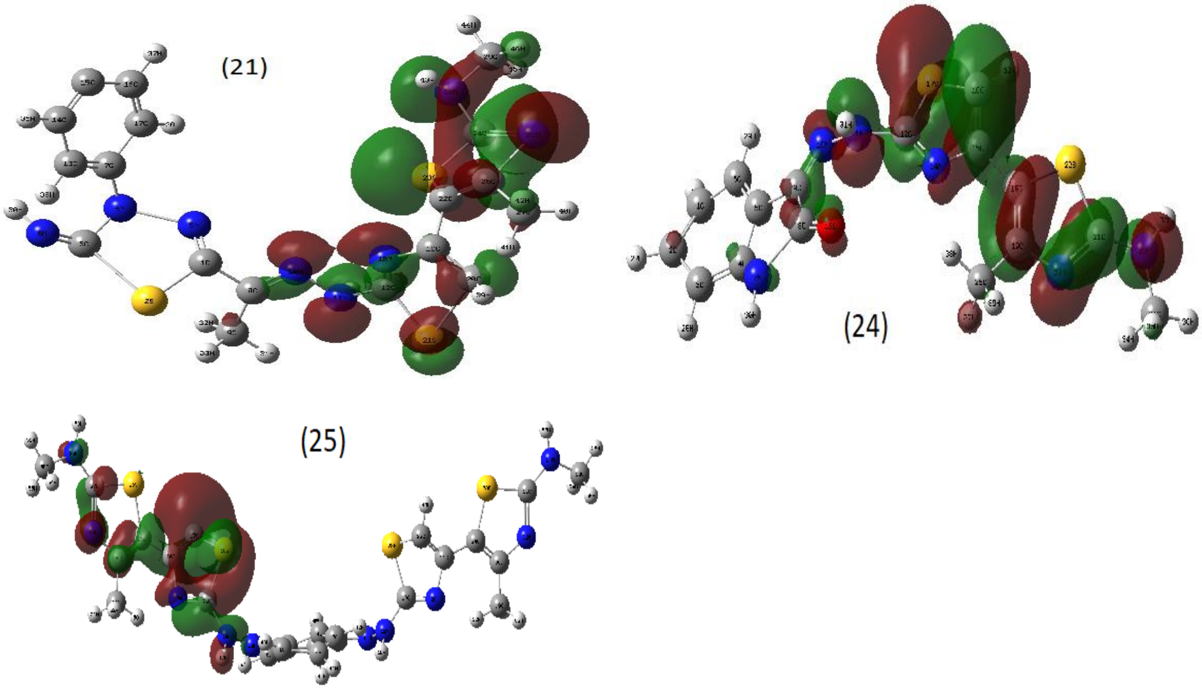

Figure 3S HOMO images of optimized structures

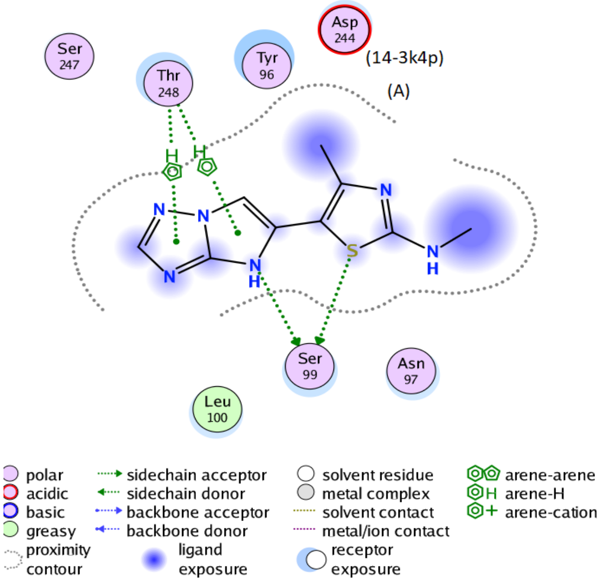
(14-3k4p)
(A)
Ser 247
Thr 248
Tyr 96
Asp 244
Ser 99
Asn 97
Leu 100
polar
acidic
basic
greasy
proximity contour
sidechain acceptor
sidechain donor
backbone acceptor
backbone donor
ligand exposure
solvent residue
metal complex
solvent contact
metal/ion contact
receptor exposure
arene-arene
arene-H
arene-cation

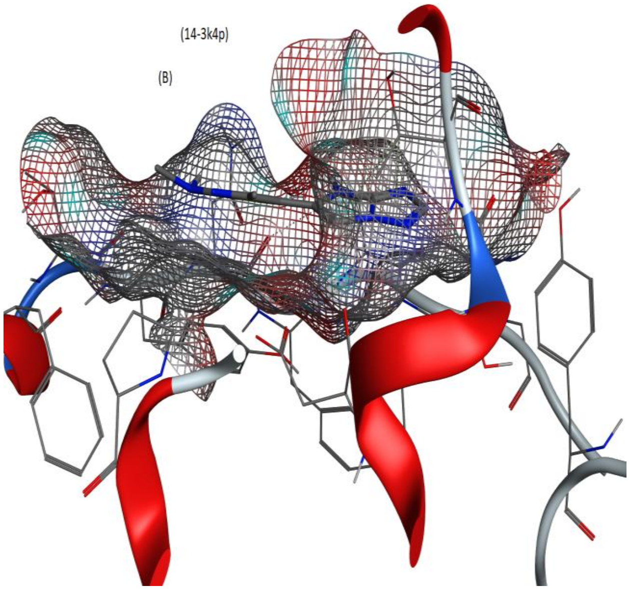
(14-3k4p)
(B)

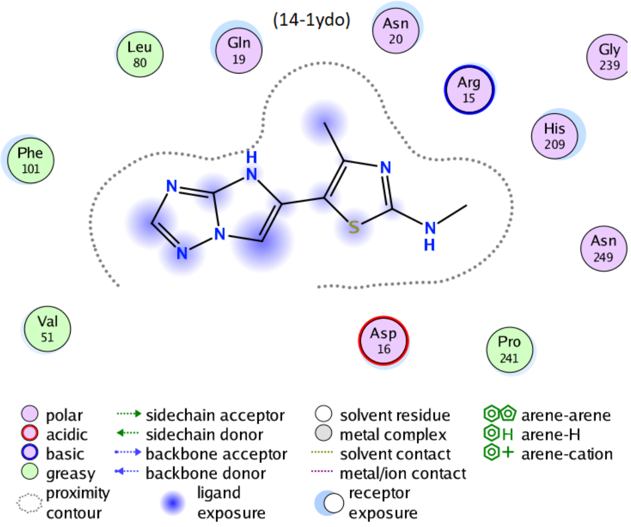
(14-1ydo)
Leu 80
Gln 19
Asn 20
Arg 15
Gly 239
His 209
Phe 101
Asn 249
Val 51
Asp 16
Pro 241
polar
acidic
basic
greasy
proximity contour
sidechain acceptor
sidechain donor
backbone acceptor
backbone donor
ligand exposure
solvent residue
metal complex
solvent contact
metal/ion contact
receptor exposure
arene-arene
arene-H
arene-cation

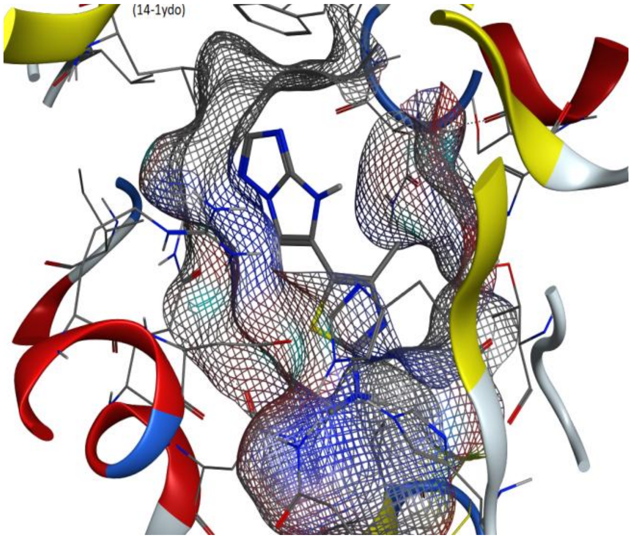
(14-1ydo)

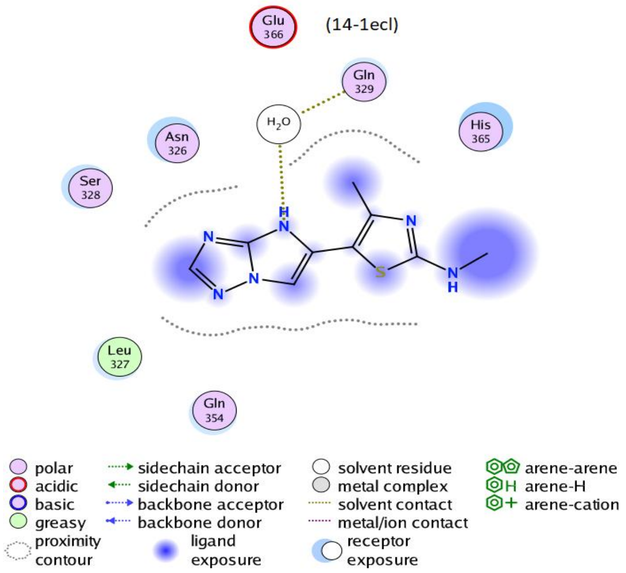
(14-1ecl)
Glu 366
Gln 329
H2O
Asn 326
His 365
Ser 328
Leu 327
Gln 354
polar
acidic
basic
greasy
proximity contour
sidechain acceptor
sidechain donor
backbone acceptor
backbone donor
ligand exposure
solvent residue
metal complex
solvent contact
metal/ion contact
receptor exposure
arene-arene
arene-H
arene-cation

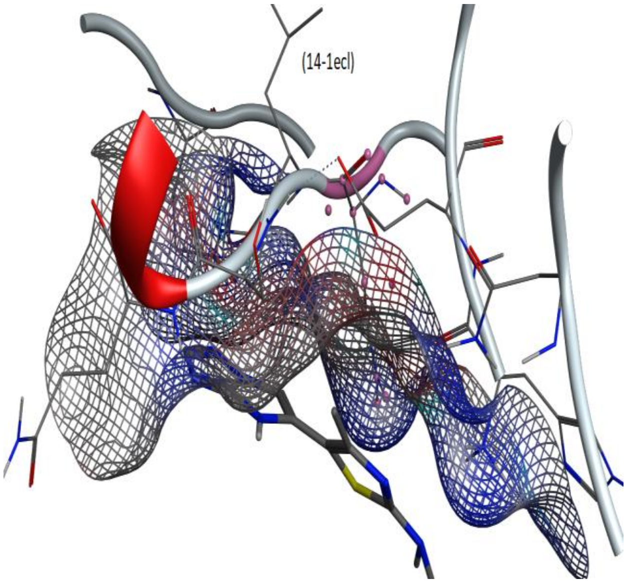
(14-1ecl)

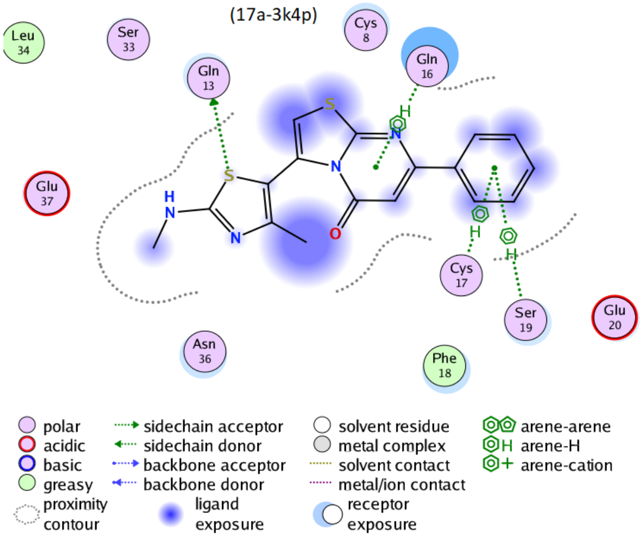
(17a-3k4p)
Leu 34
Ser 33
Cys 8
Gln 13
Gln 16
Glu 37
Cys 17
Ser 19
Glu 20
Asn 36
Phe 18
polar
acidic
basic
greasy
proximity contour
sidechain acceptor
sidechain donor
backbone acceptor
backbone donor
ligand exposure
solvent residue
metal complex
solvent contact
metal/ion contact
receptor exposure
arene-arene
arene-H
arene-cation

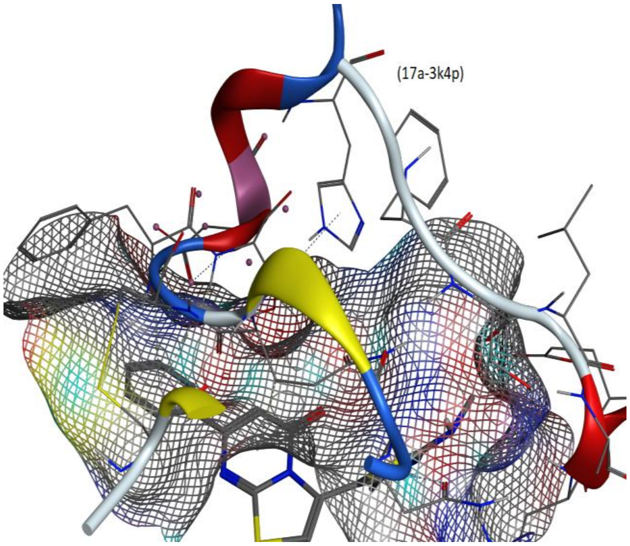
(17a-3k4p)

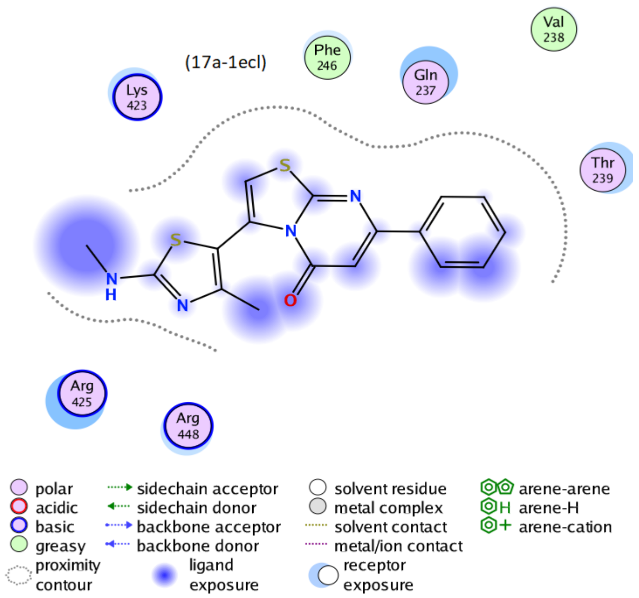
(17a-1ecl)
Lys 423
Phe 246
Gln 237
Val 238
Thr 239
Arg 425
Arg 448
polar
acidic
basic
greasy
proximity contour
sidechain acceptor
sidechain donor
backbone acceptor
backbone donor
ligand exposure
solvent residue
metal complex
solvent contact
metal/ion contact
receptor exposure
arene-arene
arene-H
arene-cation

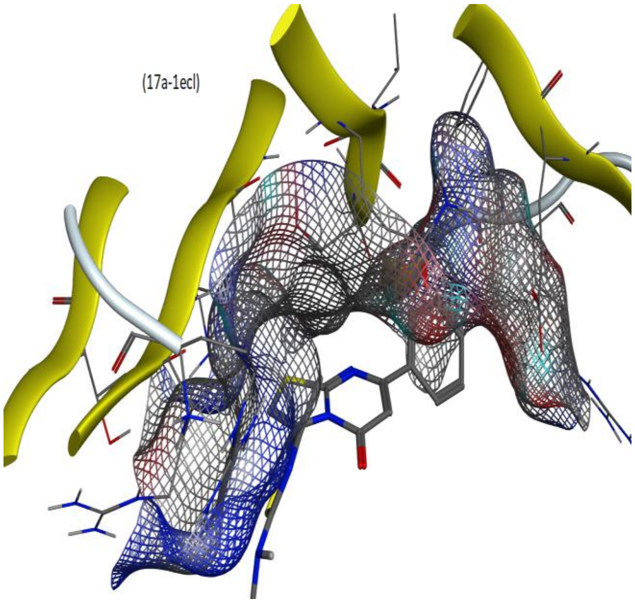
(17a-1ecl)

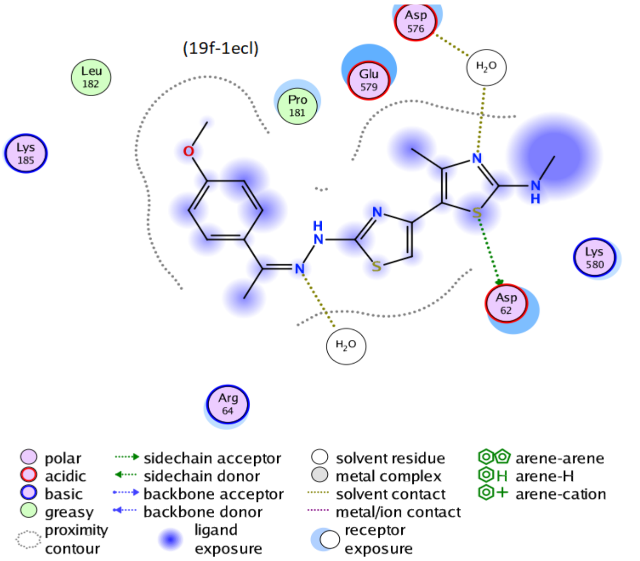
(19f-1ecl)
Asp 576
Glu 579
Pro 181
Leu 182
Lys 185
Lys 580
Asp 62
Arg 64
H2O
H2O
polar
acidic
basic
greasy
proximity contour
sidechain acceptor
sidechain donor
backbone acceptor
backbone donor
ligand exposure
solvent residue
metal complex
solvent contact
metal/ion contact
receptor exposure
arene-arene
arene-H
arene-cation

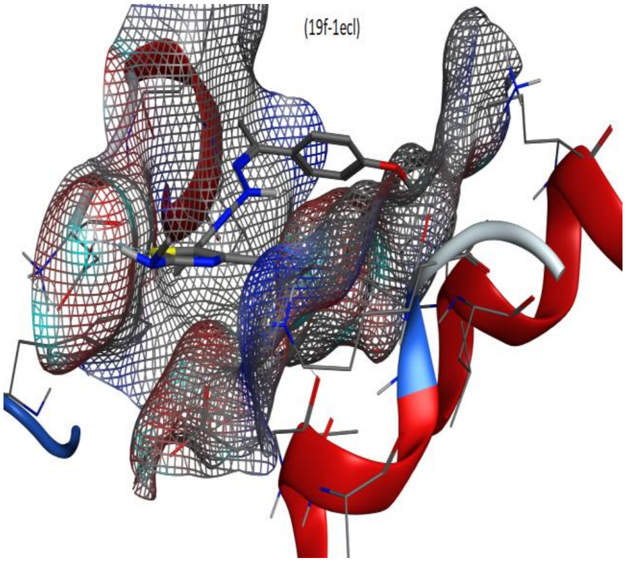
(19f-1ecl)

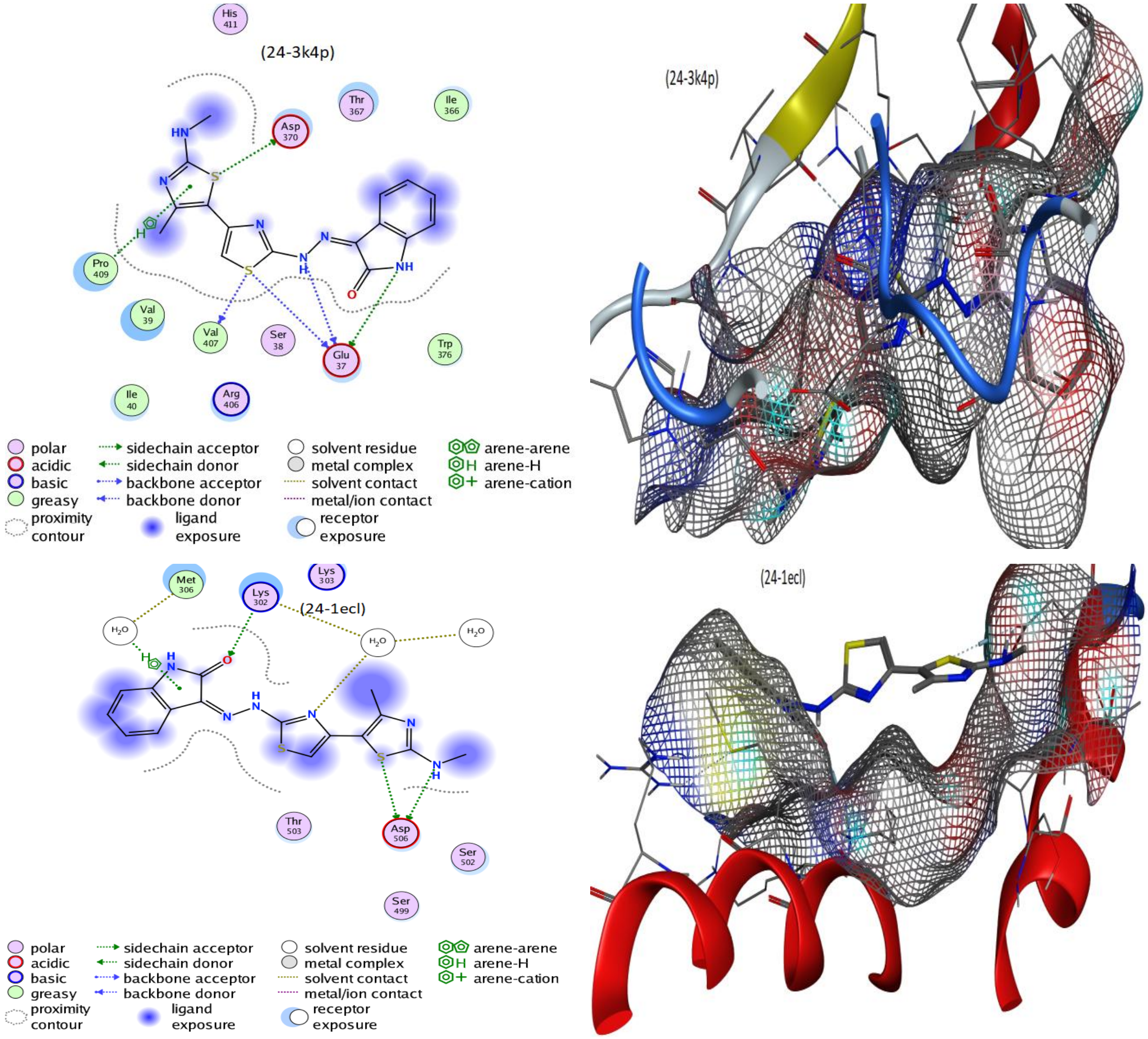

Fig. 4S. Remaining docking interactions (A) and surfaces maps (B) against, 3k4p, 1ydo & 1ecl

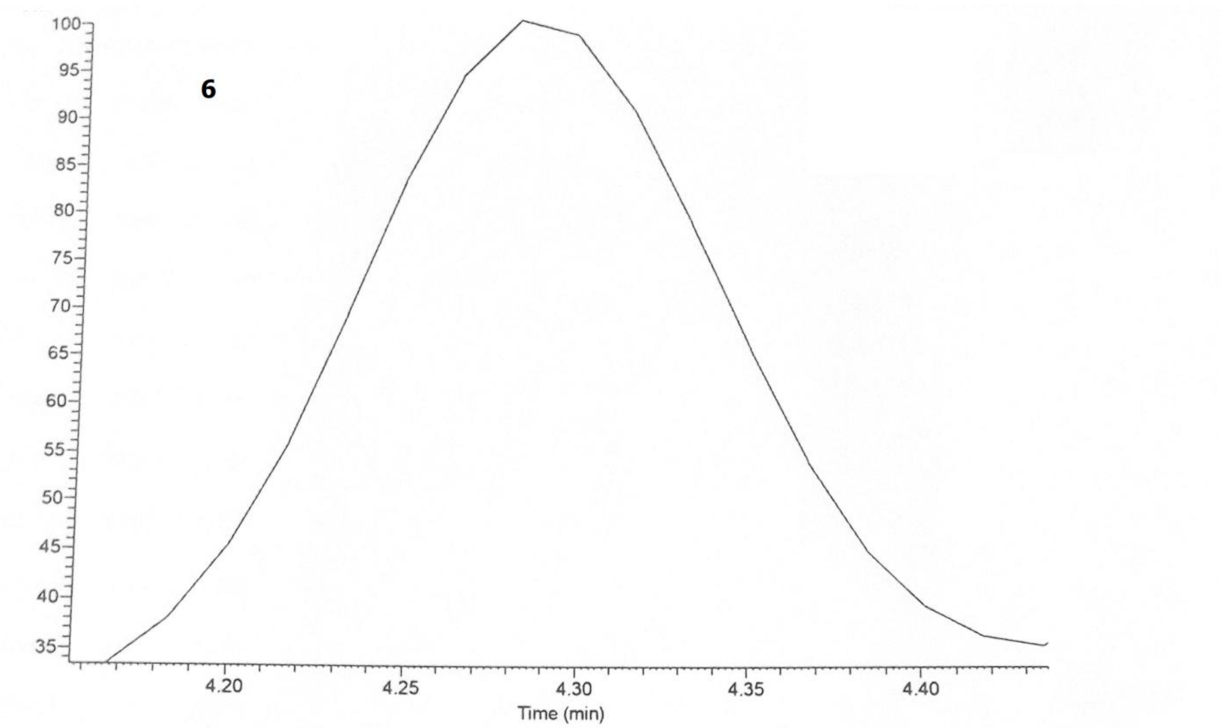
6
Time (min)

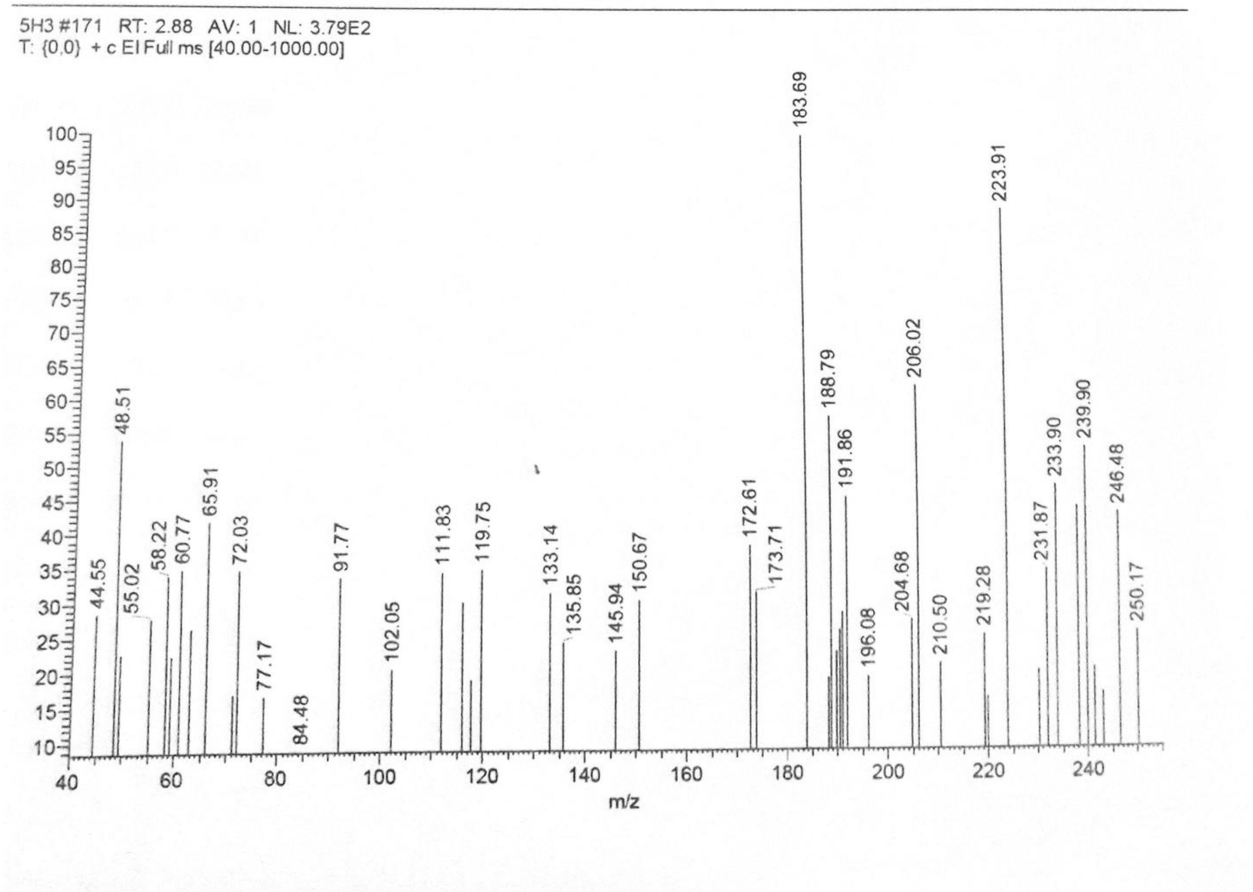
5H3 #171 RT: 2.88 AV: 1 NL: 3.79E2
T: {0,0} + c EI Full ms [40.00-1000.00]
183.69
223.91
206.02
188.79
239.90
233.90
191.86
246.48
48.51
65.91
60.77
58.22
72.03
91.77
111.83
119.75
133.14
172.61
173.71
150.67
145.94
135.85
231.87
204.68
219.28
250.17
210.50
196.08
44.55
55.02
102.05
77.17
84.48
m/z

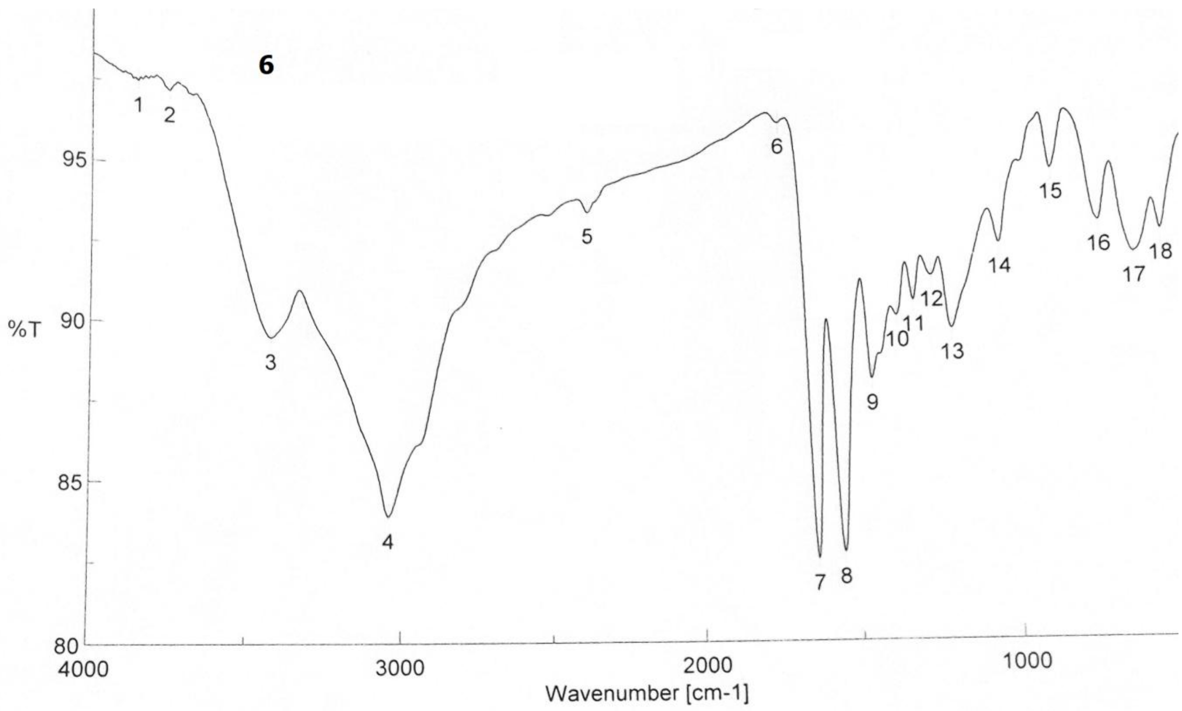

[Comments]

Sample name	SH 3
Comment	6/2018
User	IR
Division	IR
Company	MAC

[Result of Peak Picking]

No.	Position	Intensity	No.	Position	Intensity	No.	Position	Intensity
1	3855.01	97.5896	2	3752.8	97.3519	3	3417.24	89.564
4	3038.3	83.9486	5	2372.98	93.5806	6	1738.51	96.2254
7	1634.38	82.5251	8	1551.45	82.7093	9	1454.06	88.0556
10	1371.14	89.9974	11	1316.18	90.4638	12	1257.36	91.2128
13	1196.61	89.552	14	1036.55	92.1737	15	864.917	94.4247
16	717.39	92.7528	17	608.431	91.7391	18	523.579	92.4123
19	423.298	95.1315						

6

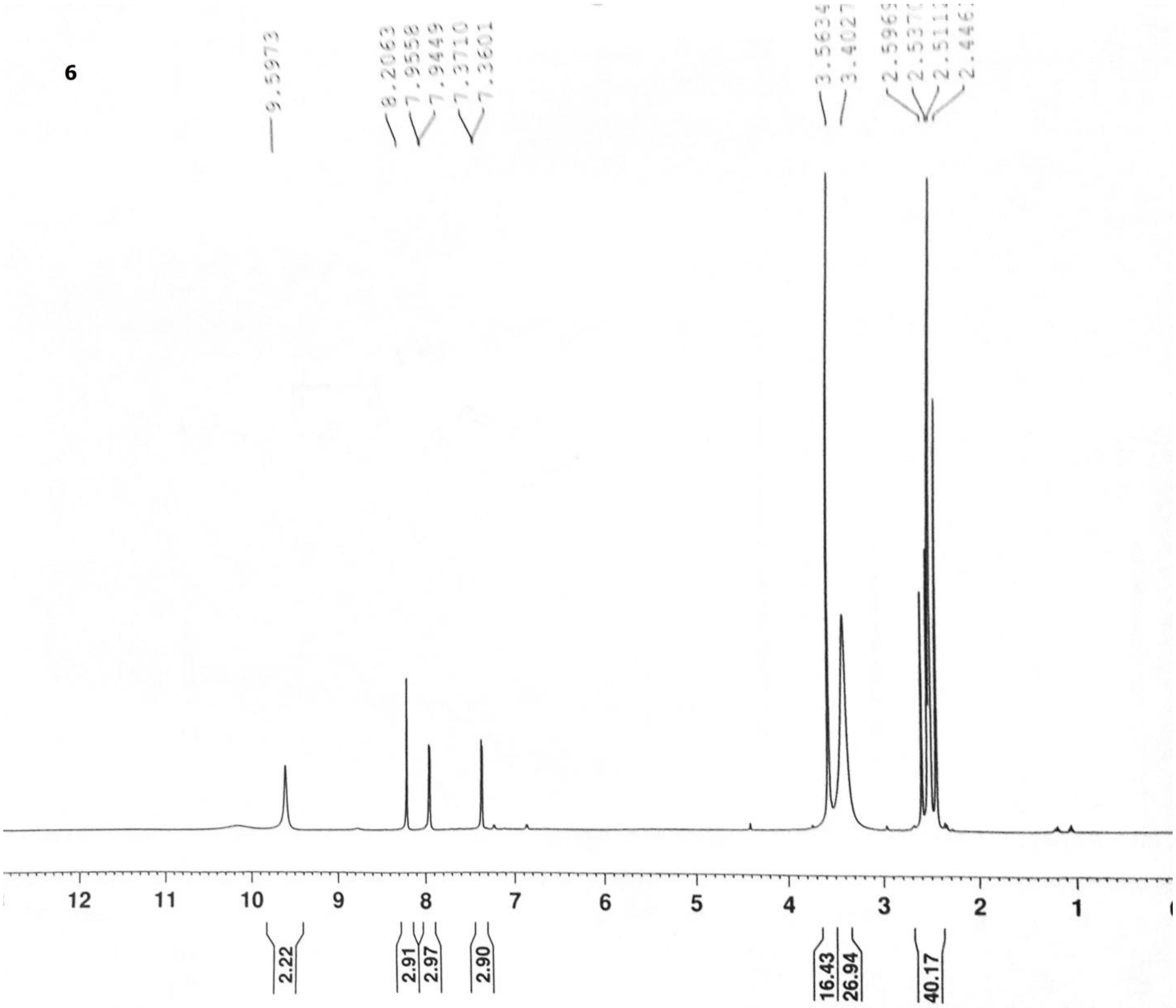
9.5973
8.2063
7.9558
7.9449
7.3710
7.3601
3.5634
3.4027
12
11
10
9
8
7
6
5
4
3
2
1
2.22
2.91
2.97
2.90
16.43
26.94
40.17

6

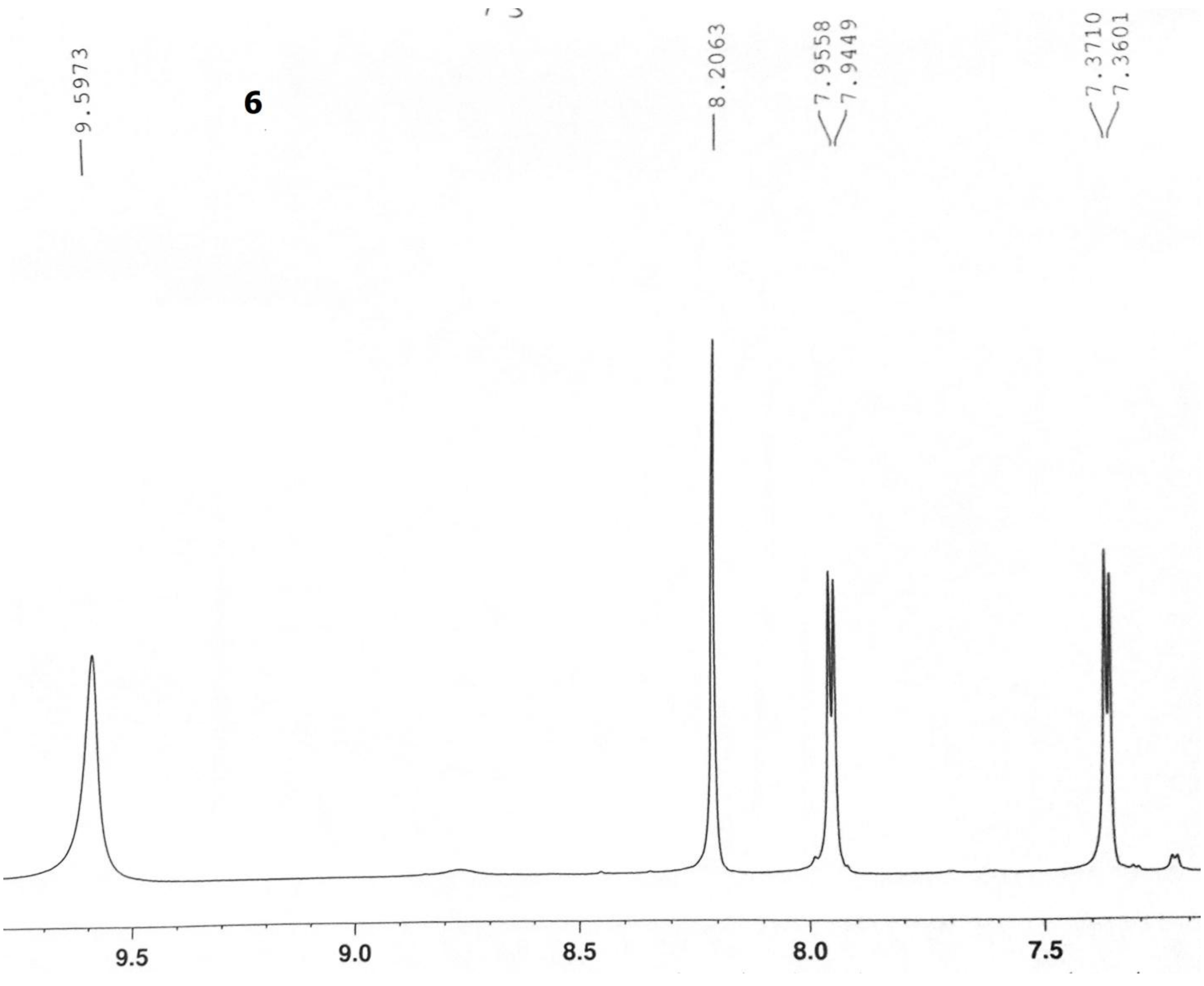

7

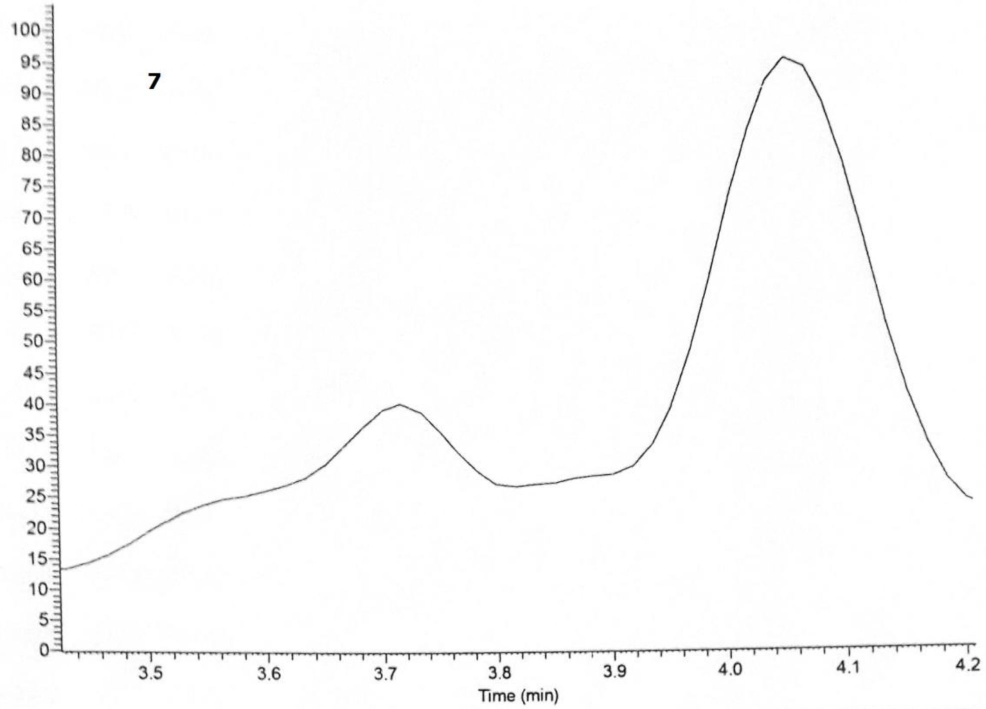

5h4 #157 RT: 2.64 AV: 1 NL: 4.04E2
T: {0,0} + c EI Full ms [40.00-1000.00]

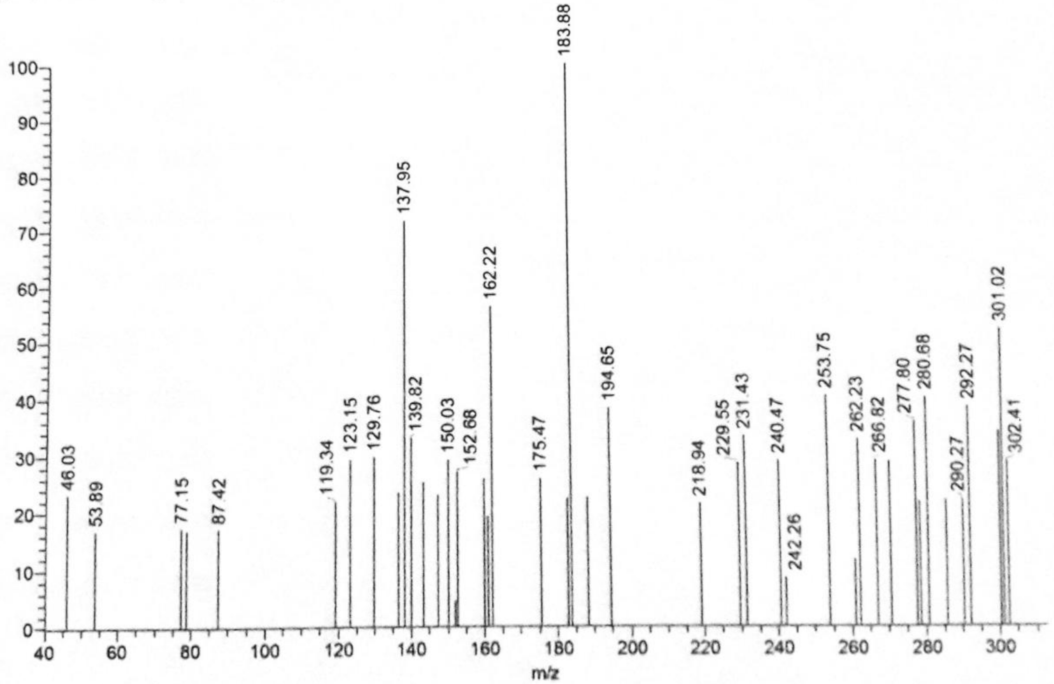

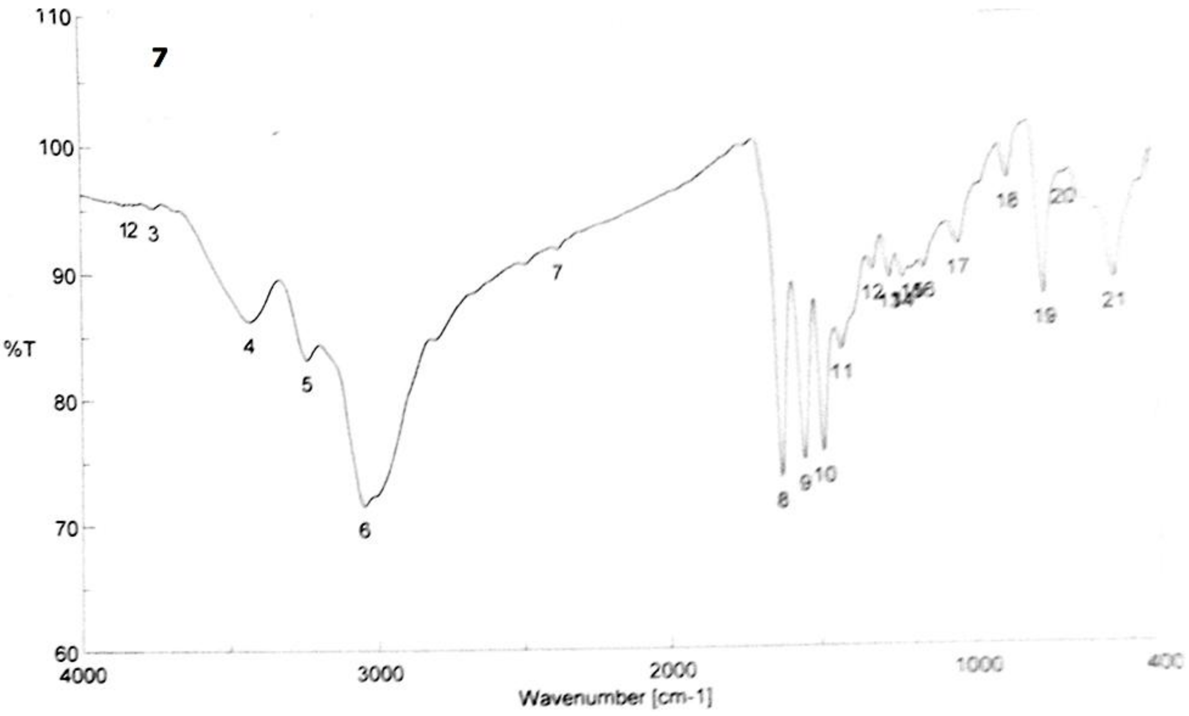

[Comments]

Sample name	SH 4
Comment	6/2018
User	IR
Division	IR
Company	MAC

[Result of Peak Picking]

No.	Position	Intensity	No.	Position	Intensity	No.	Position	Intensity
1	3855.01	95.5399	2	3823.19	95.5808	3	3753.76	95.2994
4	3432.67	86.4814	5	3238.86	83.4322	6	3047.94	71.651
7	2377.8	92.2107	8	1623.77	73.4401	9	1549.52	74.7523
10	1486.85	75.3384	11	1424.17	83.4751	12	1318.11	89.7044
13	1262.18	89.0024	14	1219.76	88.9877	15	1188.9	89.6694
16	1149.37	89.7399	17	1034.62	91.5184	18	864.917	96.6301
19	752.102	87.3453	20	679.785	96.8004	21	522.615	88.4527

7

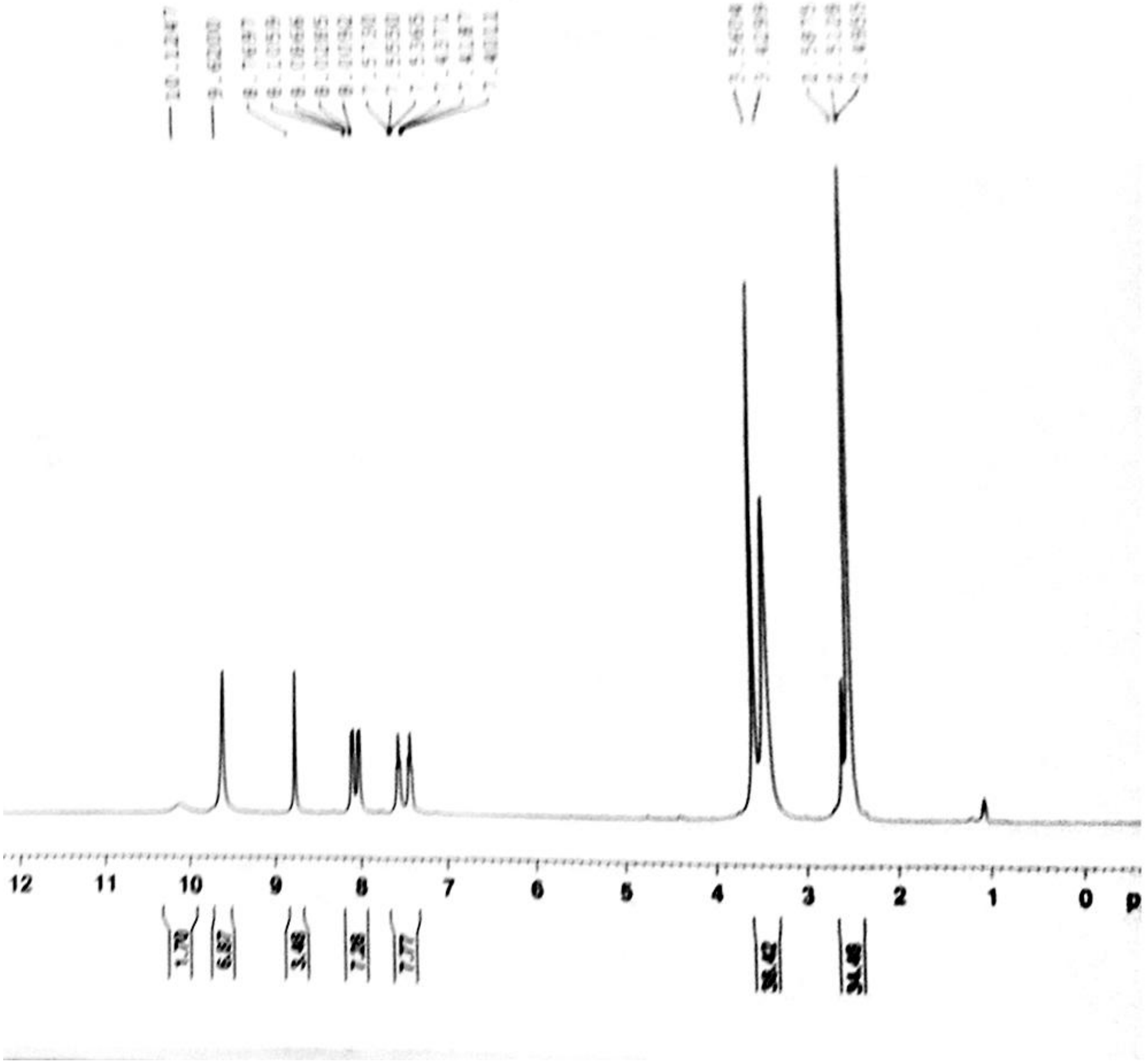

7

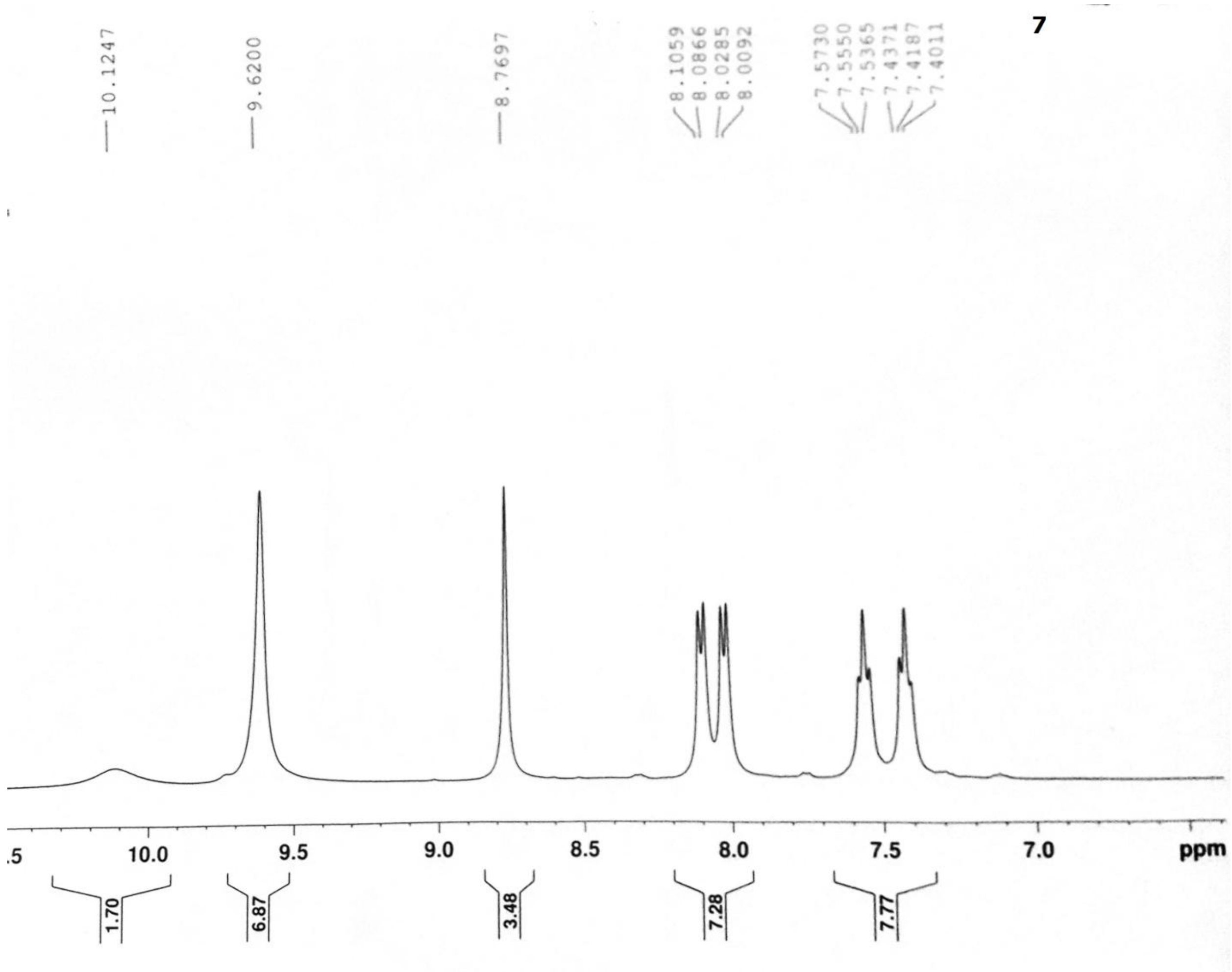
10.1247
9.6200
8.7697
8.1059
8.0866
8.0285
8.0092
7.5730
7.5550
7.5365
7.4371
7.4187
7.4011
10.0
9.5
9.0
8.5
8.0
7.5
7.0
ppm
1.70
6.87
3.48
7.28
7.77

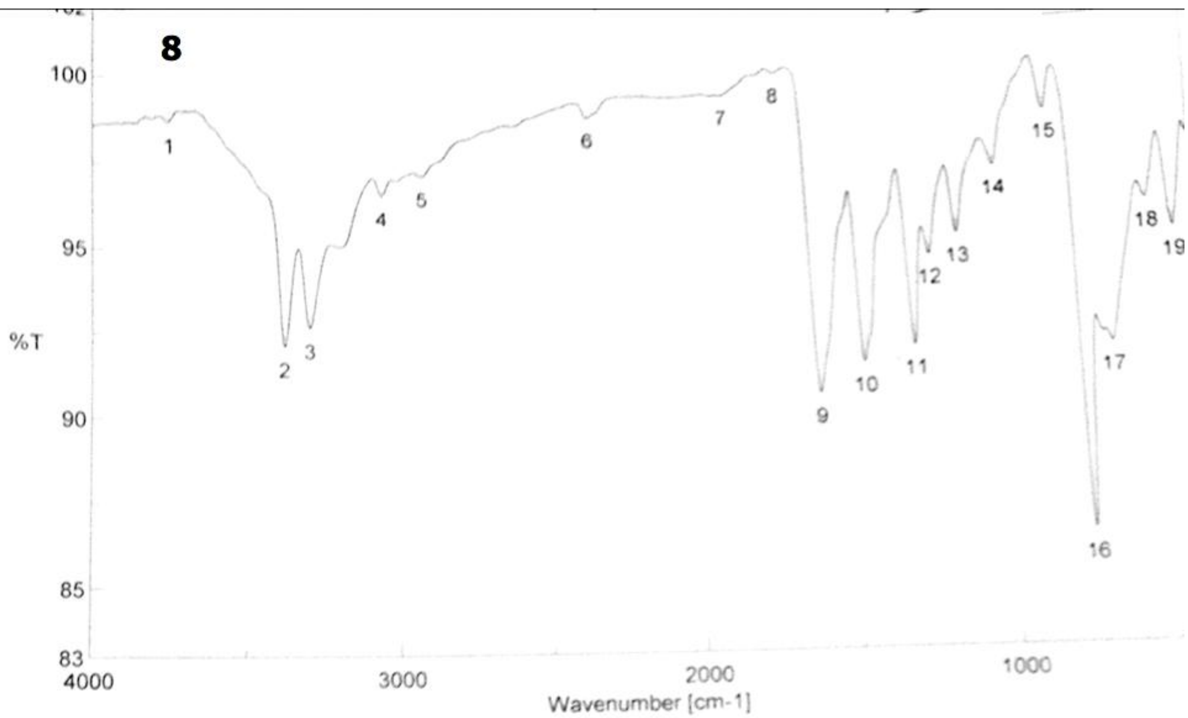

[Comments]

Sample name	SH20
Comment	9/2018
User	IR
Division	IR
Company	MAC

[Result of Peak Picking]

No.	Position	Intensity	No.	Position	Intensity	No.	Position	Intensity
1	3755.69	98.9028	2	3374.82	92.2544	3	3293.82	92.832
4	3059.51	96.9929	5	2926.45	97.6254	6	2365.26	99.5368
7	1912.07	100.128	8	1735.62	100.759	9	1612.2	90.618
10	1466.6	91.5195	11	1300.75	92.0103	12	1243.86	94.7822
13	1151.29	95.4065	14	1024.98	97.4375	15	853.347	99.0744
16	749.209	86.3346	17	665.321	91.8759	18	540.935	96.1316
19	458.011	95.2293						

12

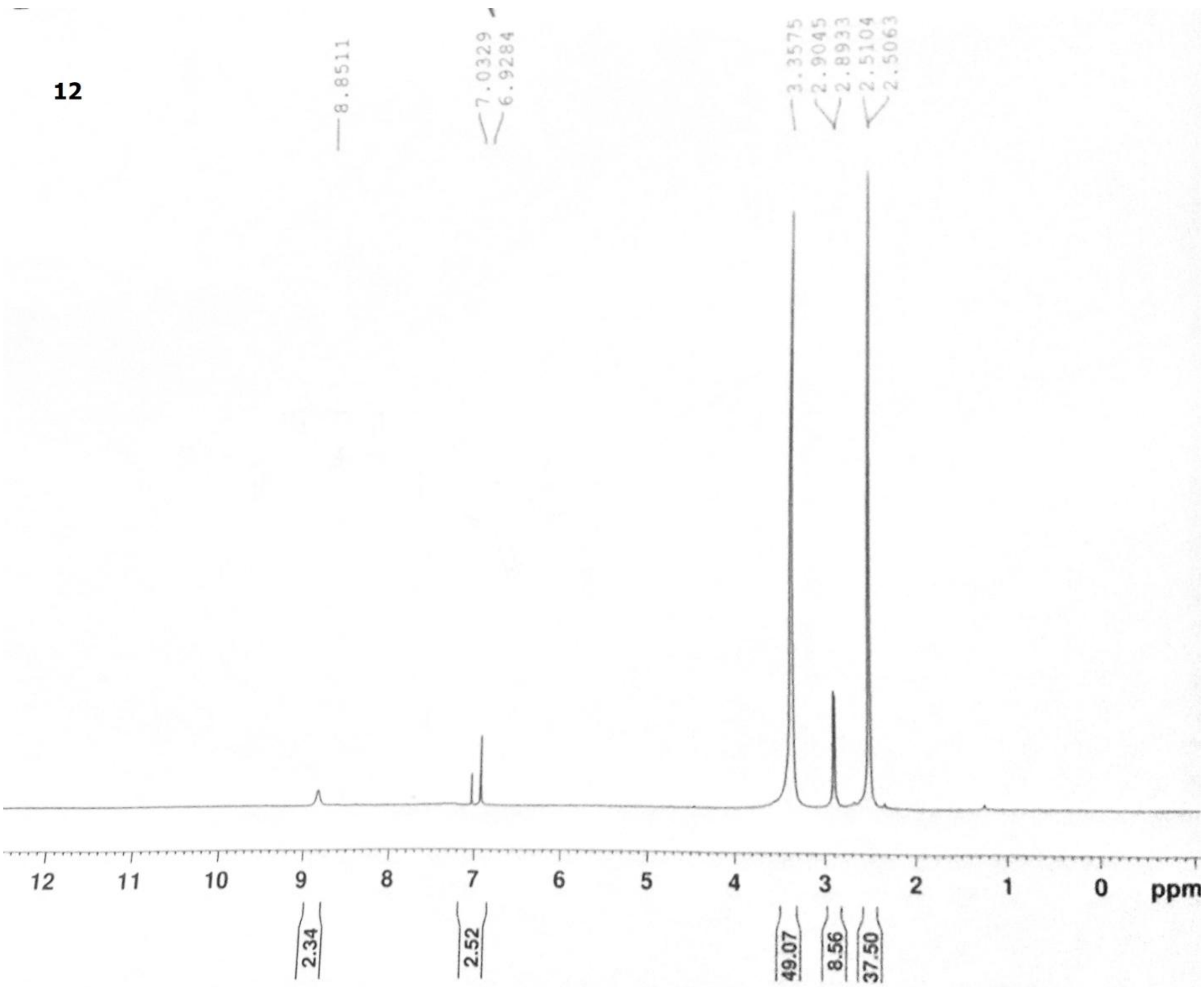
8.8511
7.0329
6.9284
3.3575
2.9045
2.8933
2.5104
2.5063
12
11
10
9
8
7
6
5
4
3
2
1
0
ppm
2.34
2.52
49.07
8.56
37.50

12

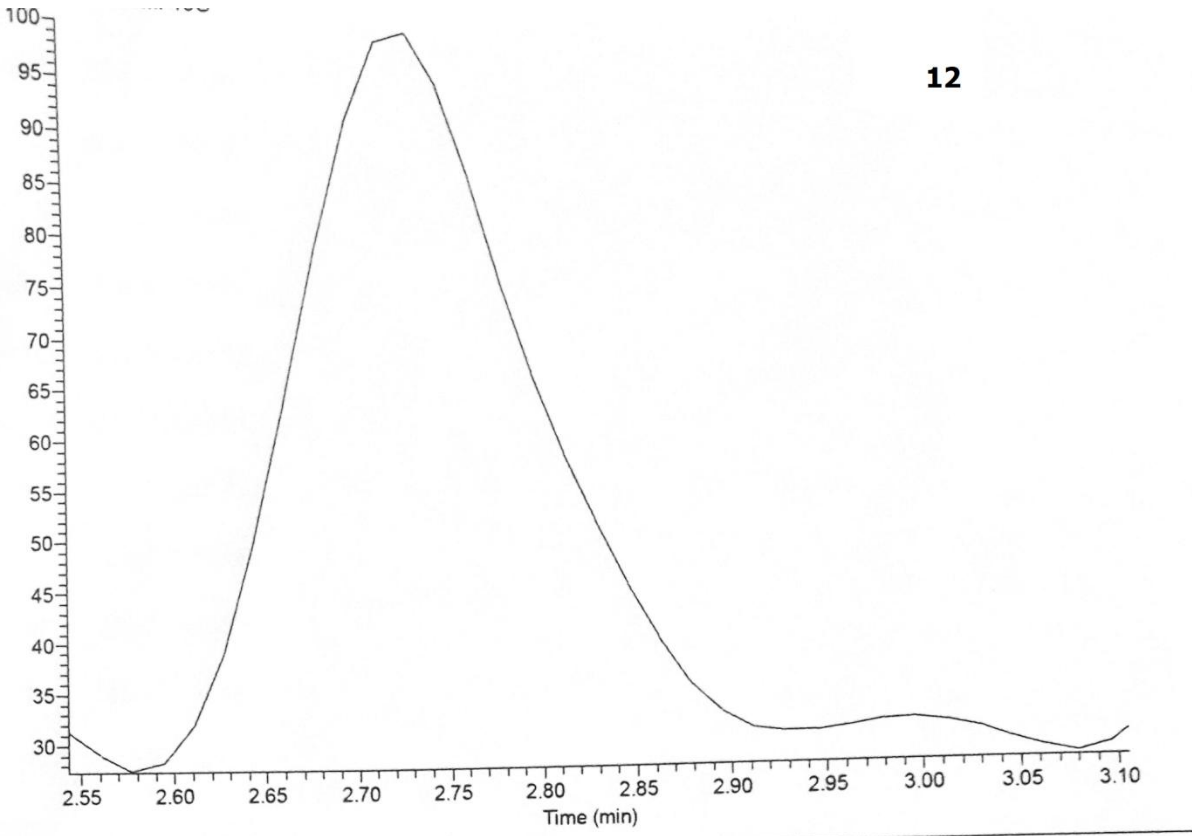

Time (min)

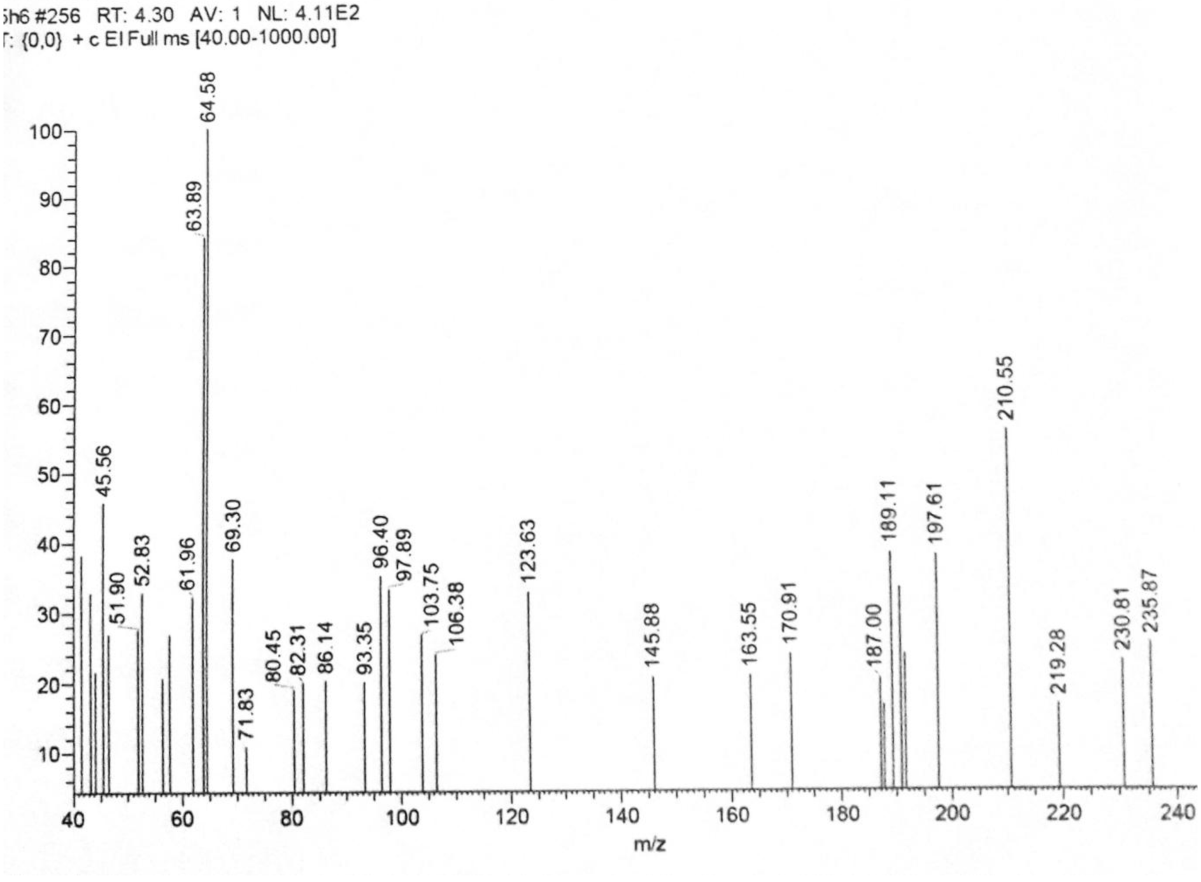

5h6 #256 RT: 4.30 AV: 1 NL: 4.11E2
T: {0,0} + c EI Full ms [40.00-1000.00]
64.58
63.89
45.56
51.90
52.83
61.96
69.30
71.83
80.45
82.31
86.14
93.35
96.40
97.89
103.75
106.38
123.63
145.88
163.55
170.91
187.00
189.11
197.61
210.55
219.28
230.81
235.87
m/z

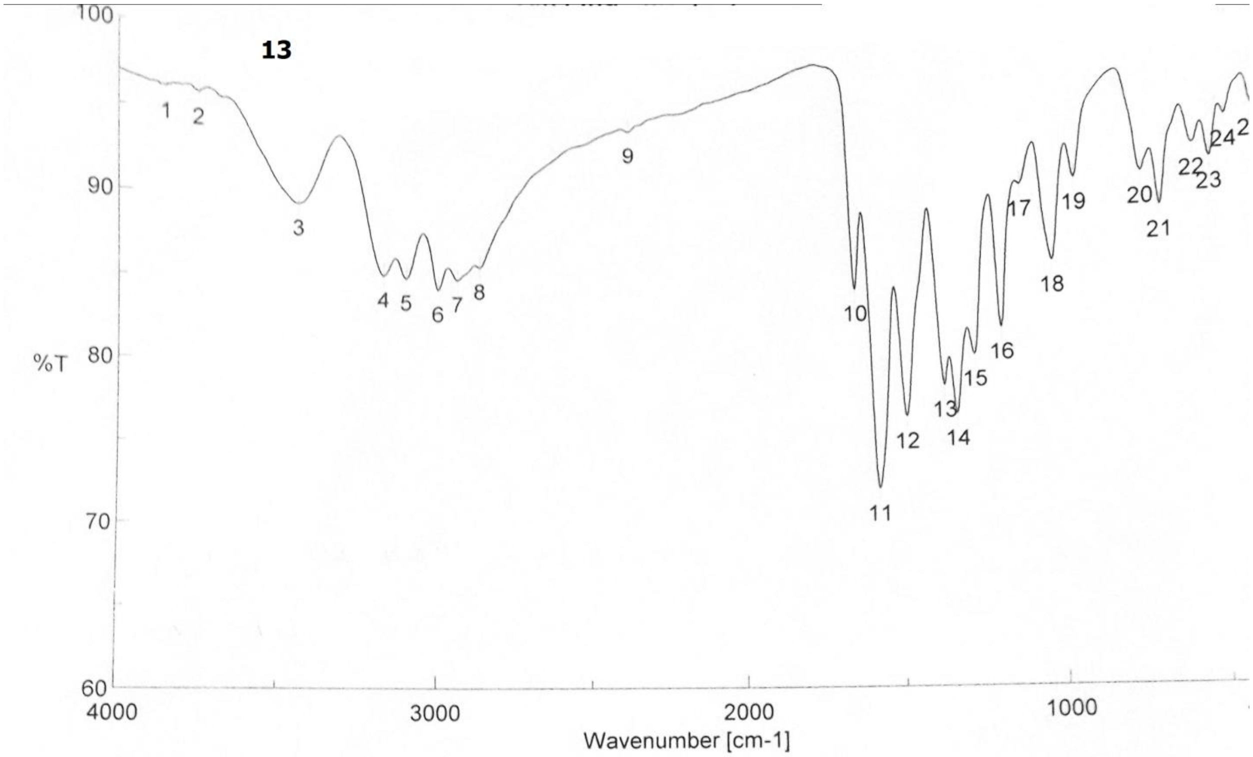

[Comments]
Sample name SH 6
Comment 6/2018
User IR
Division IR
Company MAC

[Result of Peak Picking]

No.	Position	Intensity	No.	Position	Intensity	No.	Position	Intensity
1	3855.01	96.2807	2	3752.8	96.1058	3	3438.46	89.6872
4	3165.58	85.267	5	3094.23	85.0812	6	2991.05	84.3754
[illegible]	2929.34	84.9898	8	2858.95	85.8038	9	2375.87	94.5437
0	1649.8	84.2043	11	1579.41	71.9408	12	1491.67	76.2948
3	1372.1	78.1473	14	1333.53	76.4006	15	1275.68	79.9921
6	1189.86	81.6008	17	1126.22	90.2535	18	1027.87	85.5647
9	957.484	90.4577	20	751.138	90.5705	21	693.284	88.4892
2	593.968	92.0406	23	541.899	91.1604	24	496.58	93.5907
5	414.62	94.1162						

13

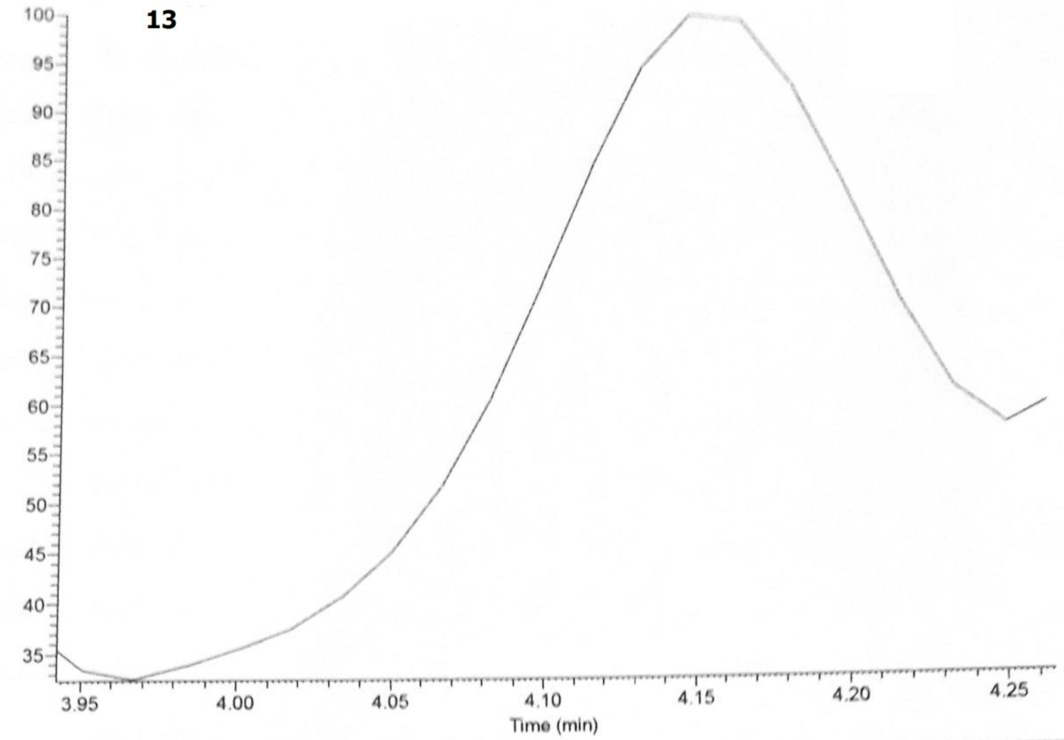
Time (min)

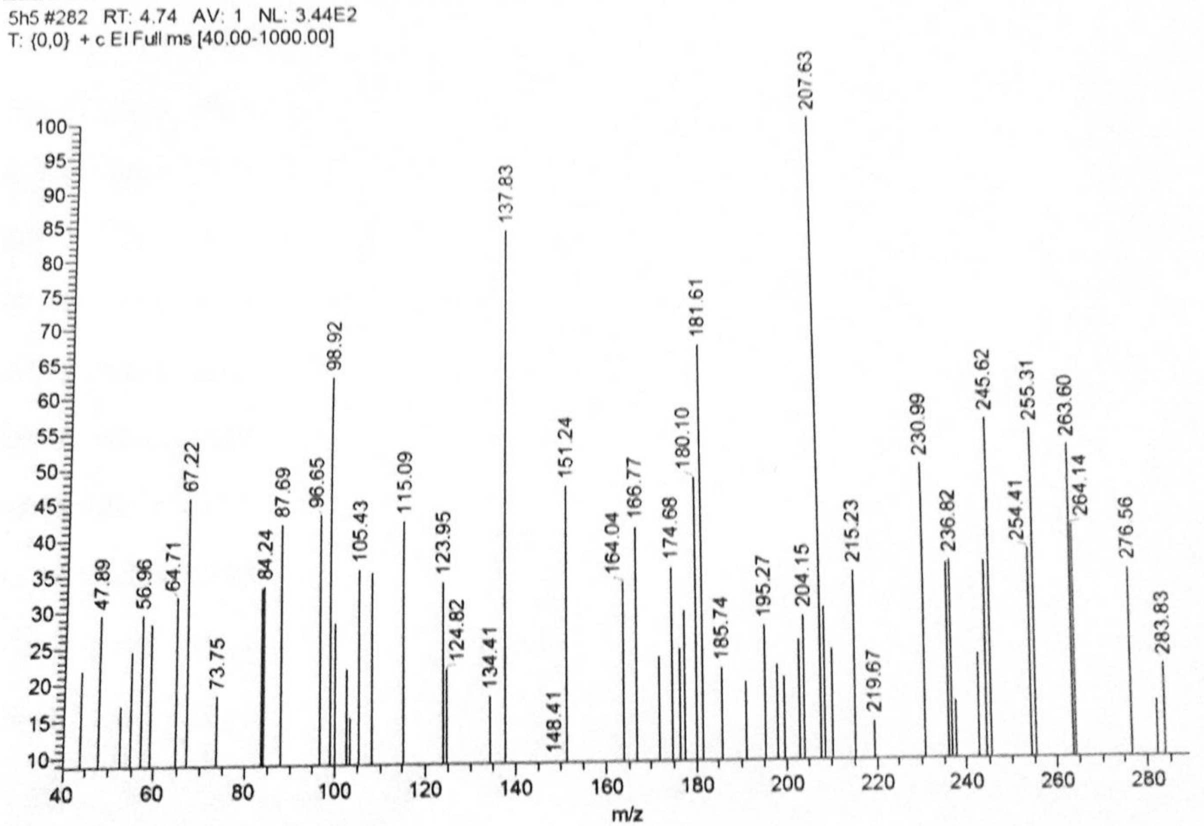
5h5 #282 RT: 4.74 AV: 1 NL: 3.44E2
T: {0,0} + c EI Full ms [40.00-1000.00]
207.63
137.83
181.61
98.92
245.62
255.31
263.60
230.99
151.24
180.10
166.77
67.22
87.69
96.65
115.09
105.43
264.14
174.68
164.04
123.95
84.24
47.89
56.96
64.71
215.23
236.82
254.41
276.56
195.27
204.15
124.82
73.75
185.74
134.41
283.83
219.67
148.41
m/z

14

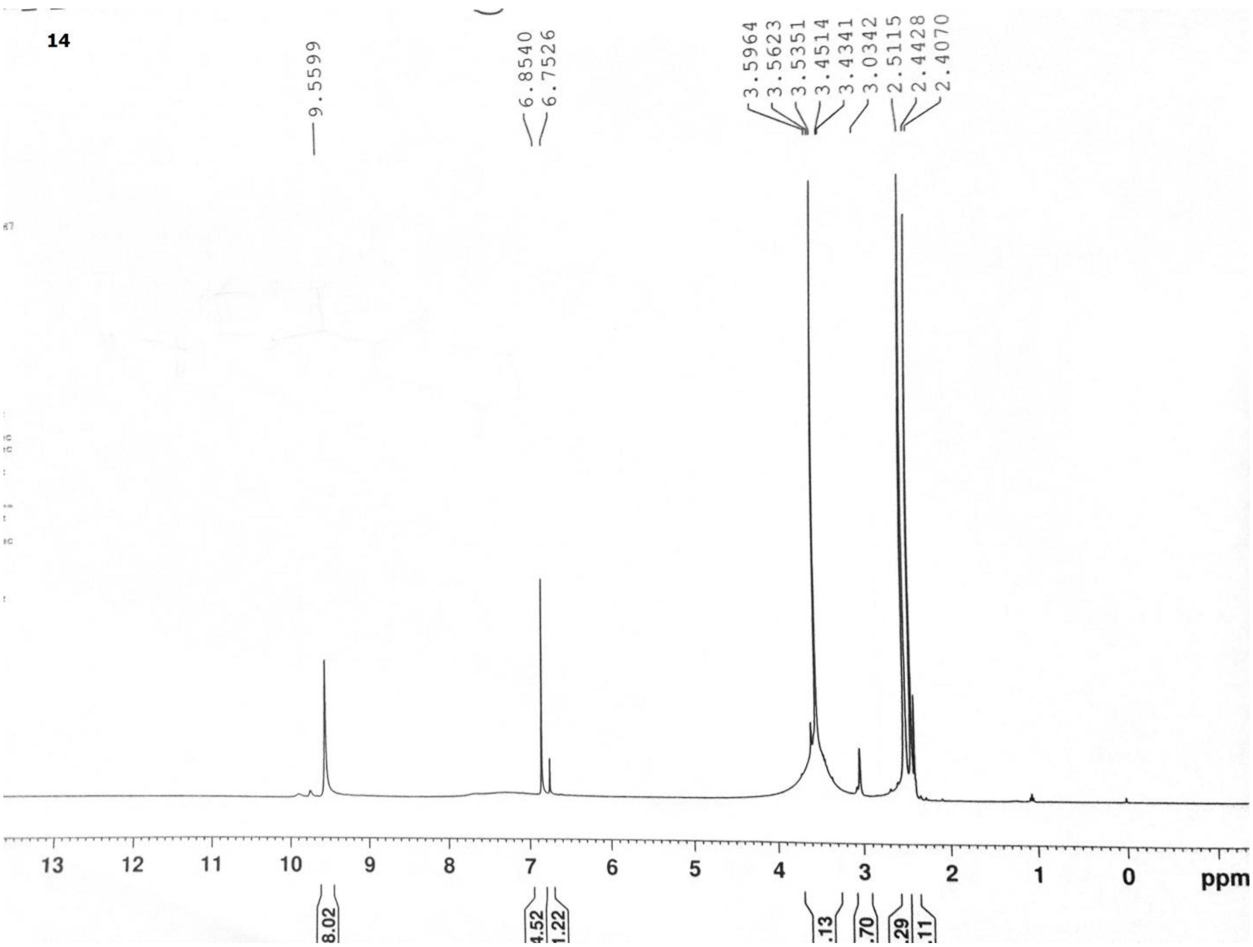
9.5599
6.8540
6.7526
3.5964
3.5623
3.5351
3.4514
3.4341
3.0342
2.5115
2.4428
2.4070
13 12 11 10 9 8 7 6 5 4 3 2 1 0 ppm
8.02
4.52
1.22
.13
.70
.29
.11

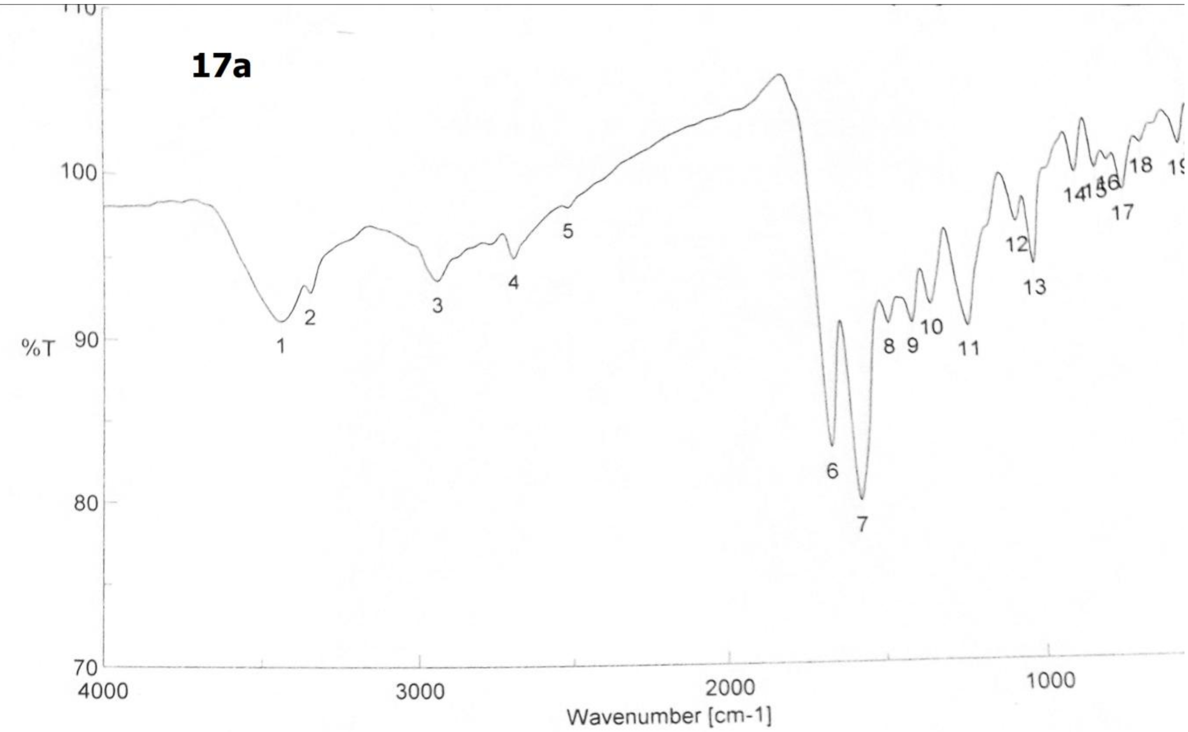

[Comments]

Sample name	SH 17
Comment	6/2018
User	IR
Division	IR
Company	MAC

[Result of Peak Picking]

No.	Position	Intensity	No.	Position	Intensity	No.	Position	Intensity
1	3433.64	91.427	2	3338.18	93.2701	3	2926.45	94.1149
4	2674.78	95.6015	5	2491.58	98.9628	6	1653.66	83.1979
7	1564.95	79.8526	8	1455.99	90.9884	9	1380.78	91.0034
10	1319.07	92.1345	11	1201.43	90.6785	12	1033.66	97.1217
13	983.518	94.3792	14	842.74	99.9796	15	778.136	100.131
16	737.639	100.543	17	695.212	98.623	18	632.537	101.463
19	514.901	101.163	20	458.011	102.308			

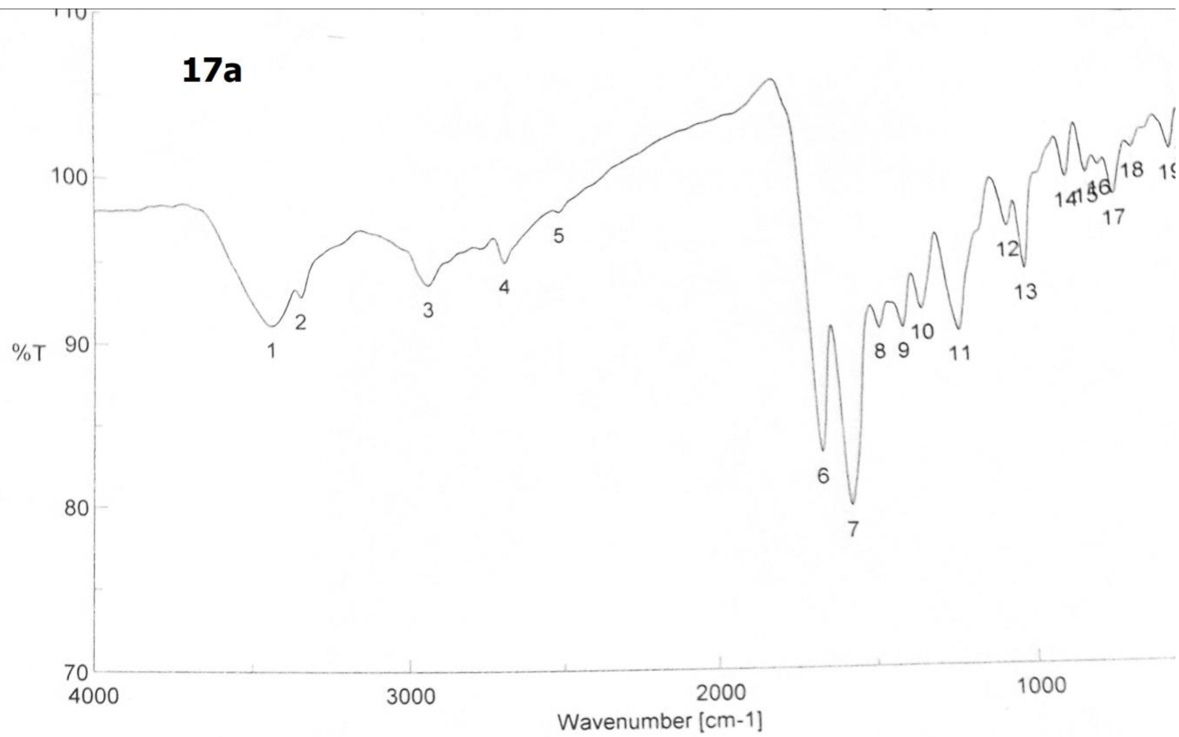

[Comments]

Sample name	SH 17
Comment	6/2018
User	IR
Division	IR
Company	MAC

[Result of Peak Picking]

No.	Position	Intensity	No.	Position	Intensity	No.	Position	Intensity
1	3433.64	91.427	2	3338.18	93.2701	3	2926.45	94.1149
4	2674.78	95.6015	5	2491.58	98.9628	6	1653.66	83.1979
7	1564.95	79.8526	8	1455.99	90.9884	9	1380.78	91.0034
10	1319.07	92.1345	11	1201.43	90.6785	12	1033.66	97.1217
13	983.518	94.3792	14	842.74	99.9796	15	778.136	100.131
16	737.639	100.543	17	695.212	98.623	18	632.537	101.463
19	514.901	101.163	20	458.011	102.308			

17a

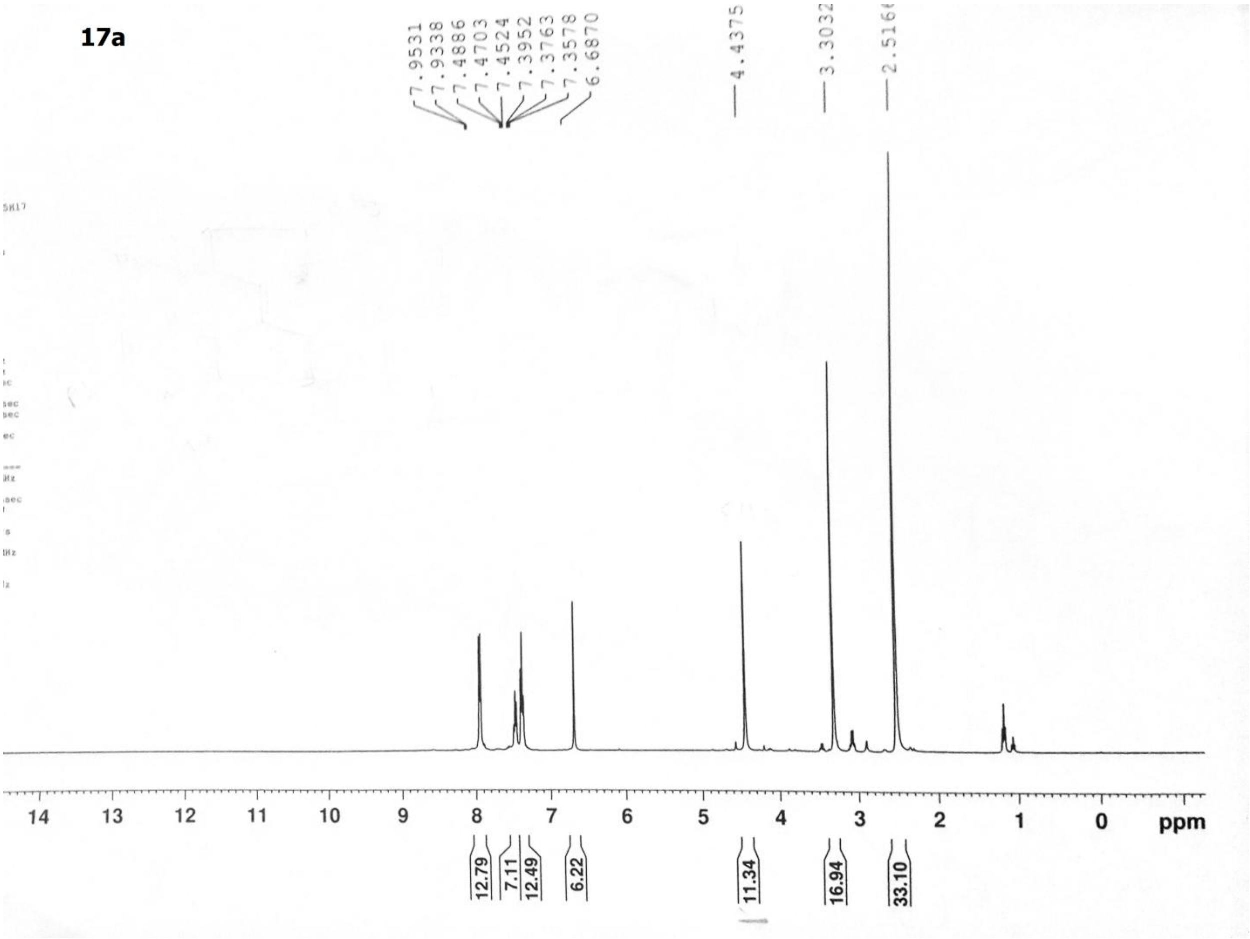
7.9531
7.9338
7.4886
7.4703
7.4524
7.3952
7.3763
7.3578
6.6870
4.4375
3.3032
14
13
12
11
10
9
8
7
6
5
4
3
2
1
0
ppm
12.79
7.11
12.49
6.22
11.34
16.94
33.10

17a

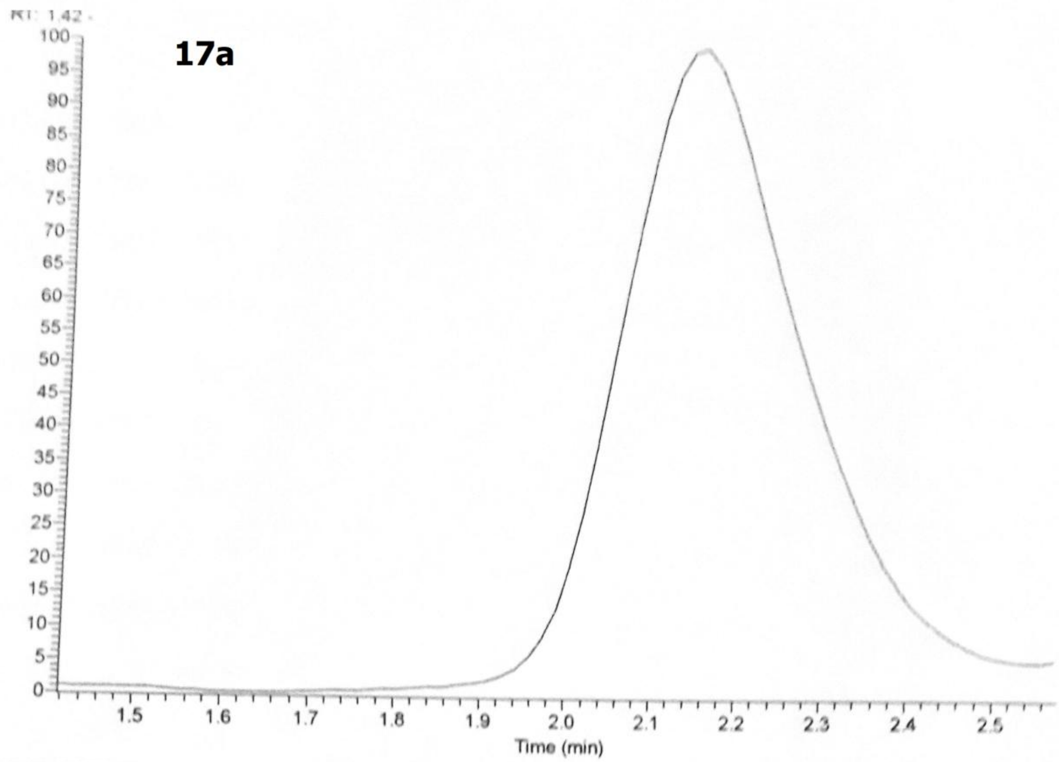

5h17 #192 RT: 3.23 AV: 1 NL: 2.77E2
T: {0,0} + c EI Full ms [40.00-1000.00]

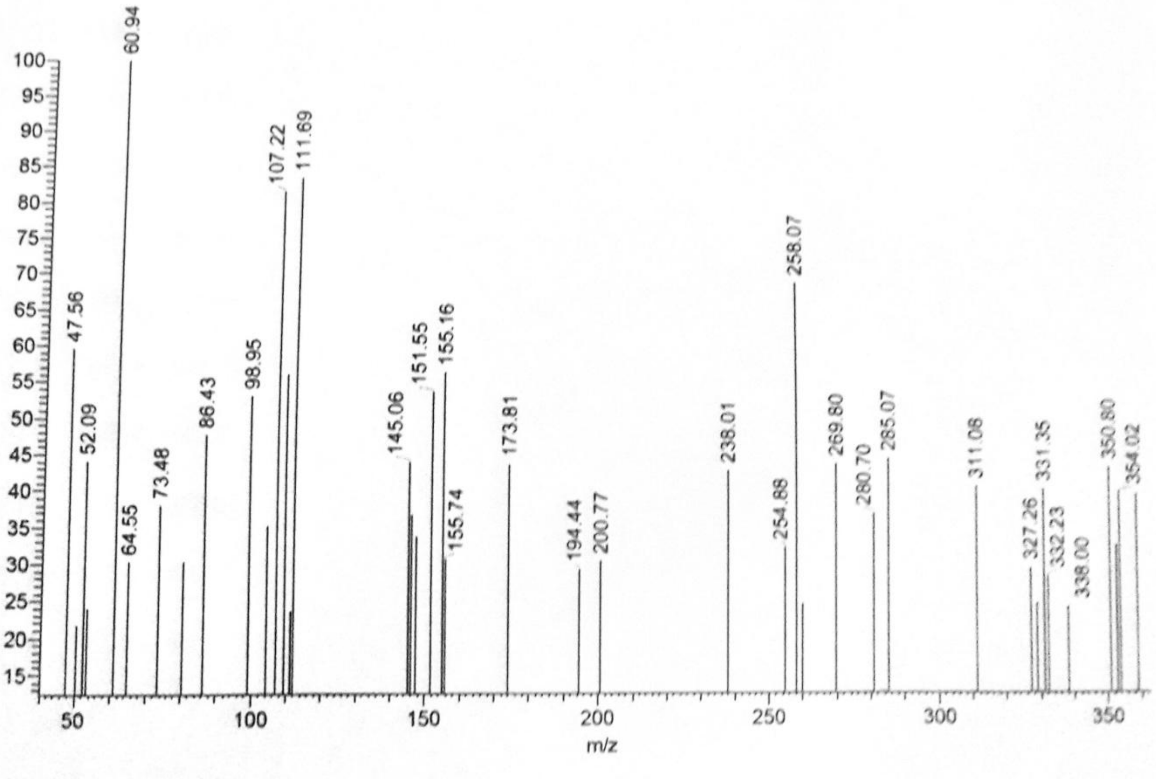

17a

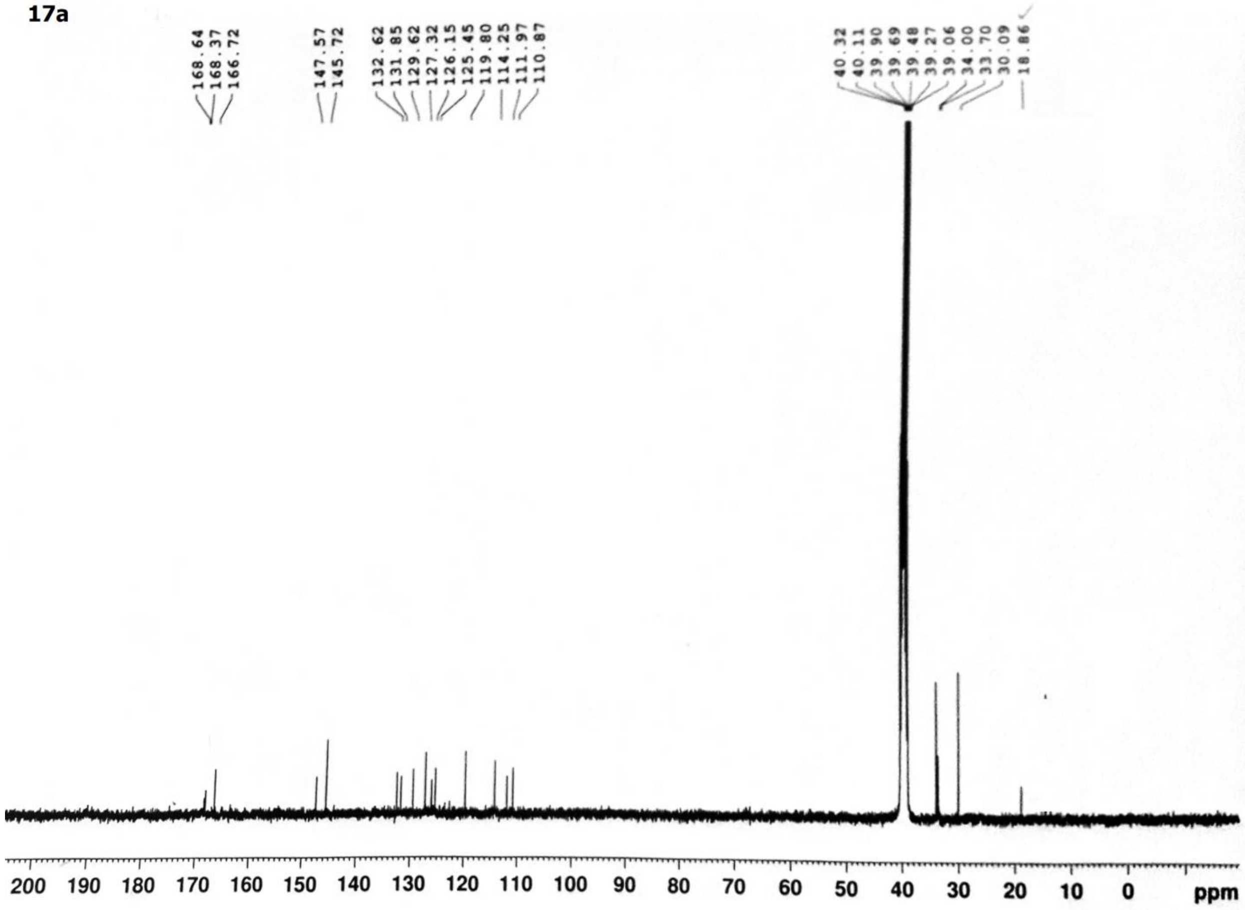
168.64
168.37
166.72
147.57
145.72
132.62
131.85
129.62
127.32
126.15
125.45
119.80
114.25
111.97
110.87
40.32
40.11
39.90
39.69
39.48
39.27
39.06
34.00
33.70
30.09
18.86
200 190 180 170 160 150 140 130 120 110 100 90 80 70 60 50 40 30 20 10 0 ppm

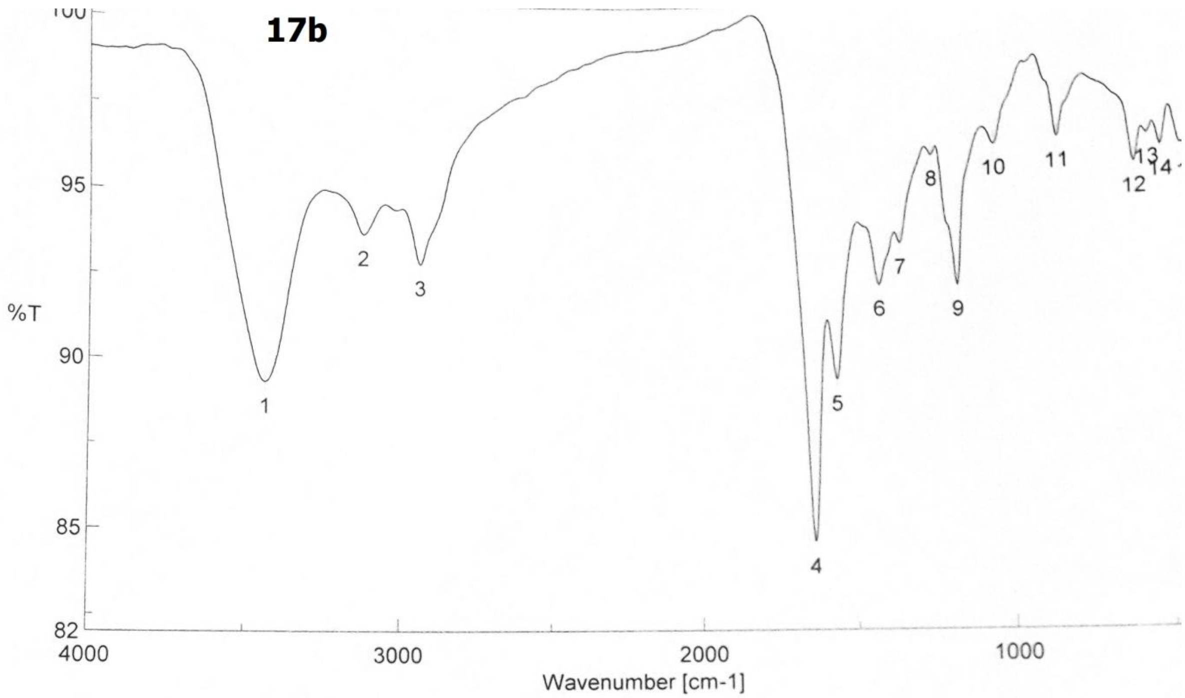

[Comments]

Sample name	SH 18
Comment	6/2018
User	IR
Division	IR
Company	MAC

[Result of Peak Picking]

No.	Position	Intensity	No.	Position	Intensity	No.	Position	Intensity
1	3430.74	89.348	2	3114.47	93.8009	3	2928.38	92.9064
4	1636.3	84.5798	5	1560.13	89.3871	6	1417.42	92.2107
7	1347.03	93.4573	8	1240	96.0959	9	1163.83	92.1475
10	1037.52	96.3368	11	835.026	96.4471	12	593.968	95.5555
13	552.506	96.3766	14	511.044	96.0017	15	422.334	95.8984

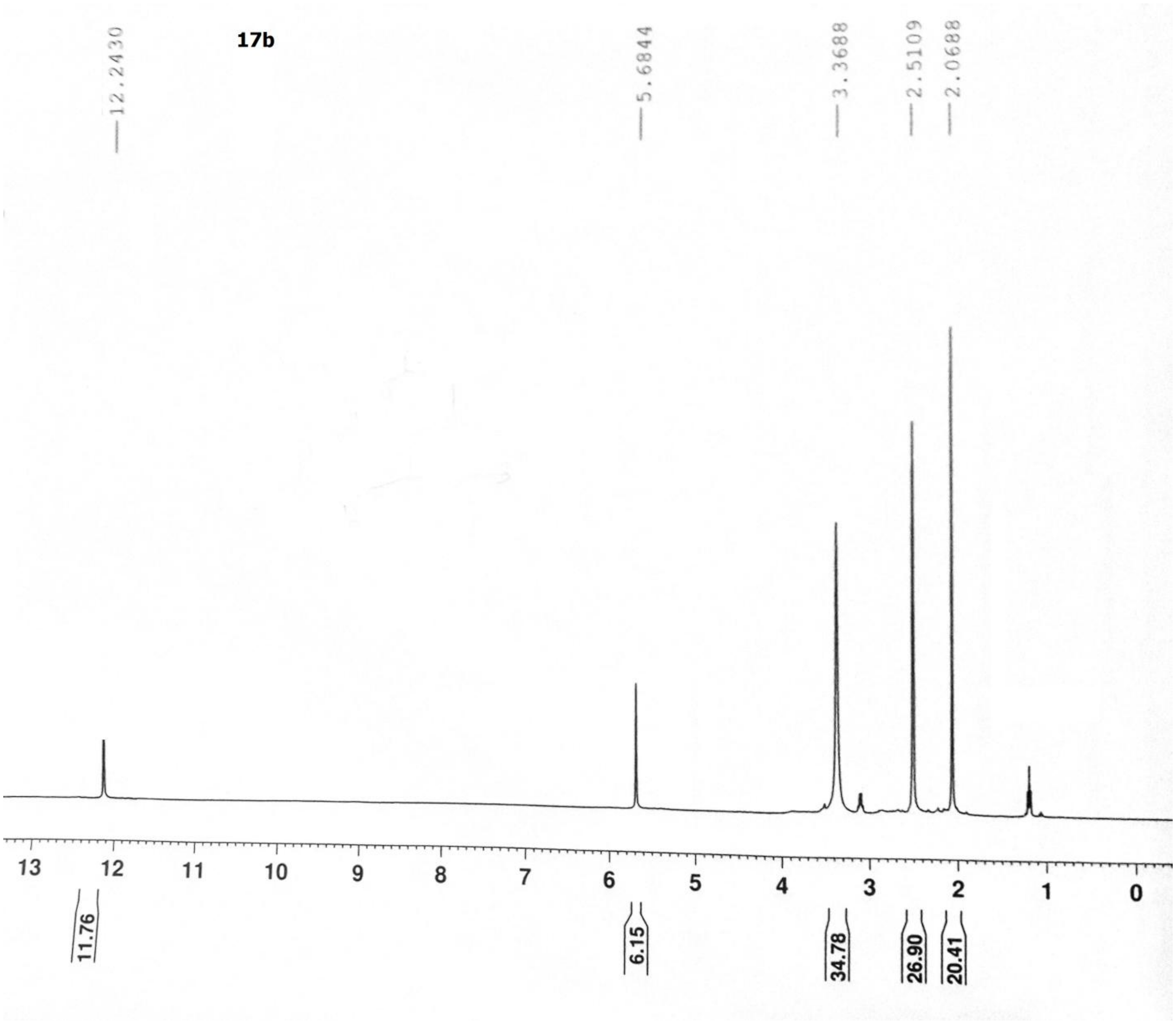
17b
12.2430
5.6844
3.3688
2.5109
2.0688
13
12
11
10
9
8
7
6
5
4
3
2
1
0
11.76
6.15
34.78
26.90
20.41

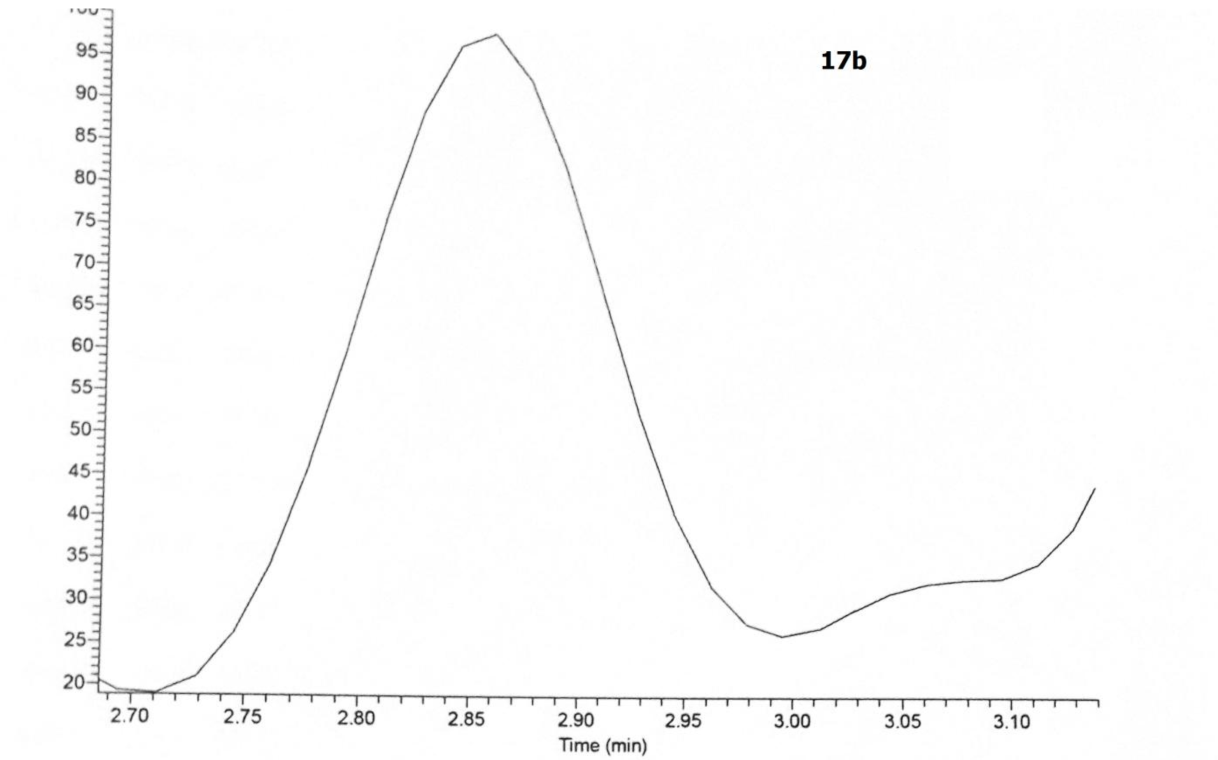

5h18 #193 RT: 3.25 AV: 1 NL: 4.85E2
T: {0,0} + c EI Full ms [40.00-1000.00]

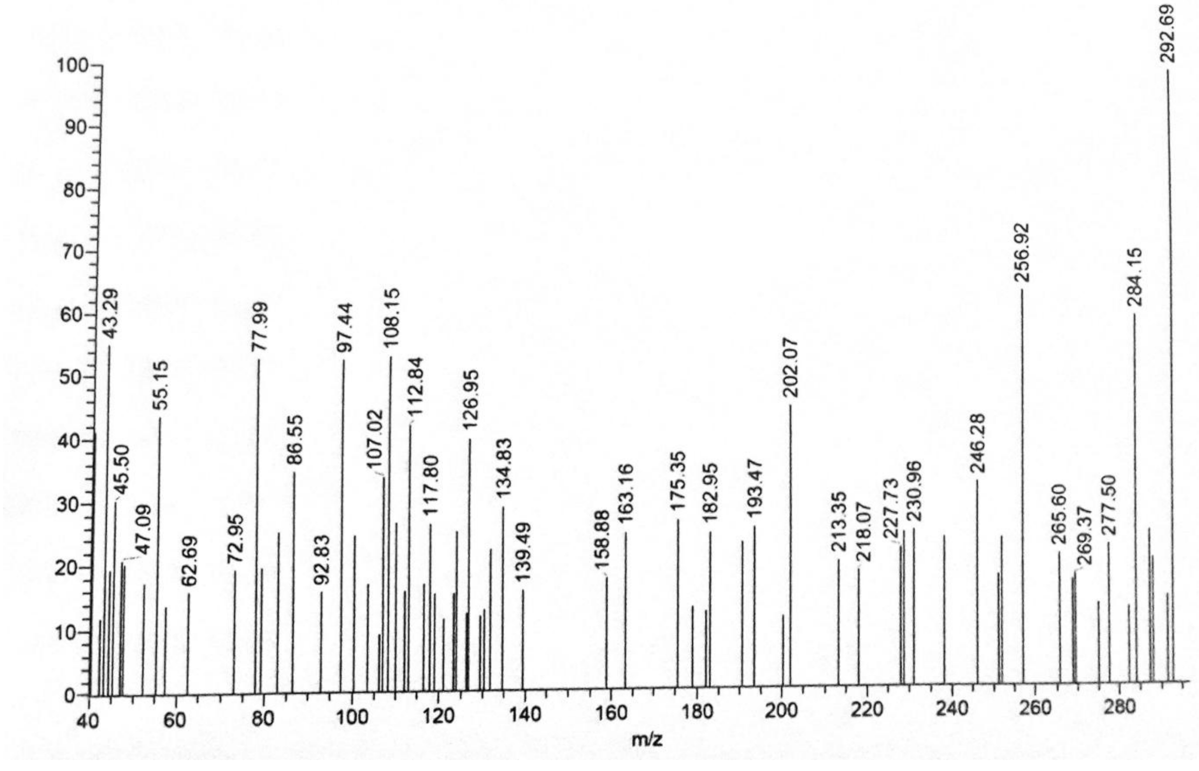

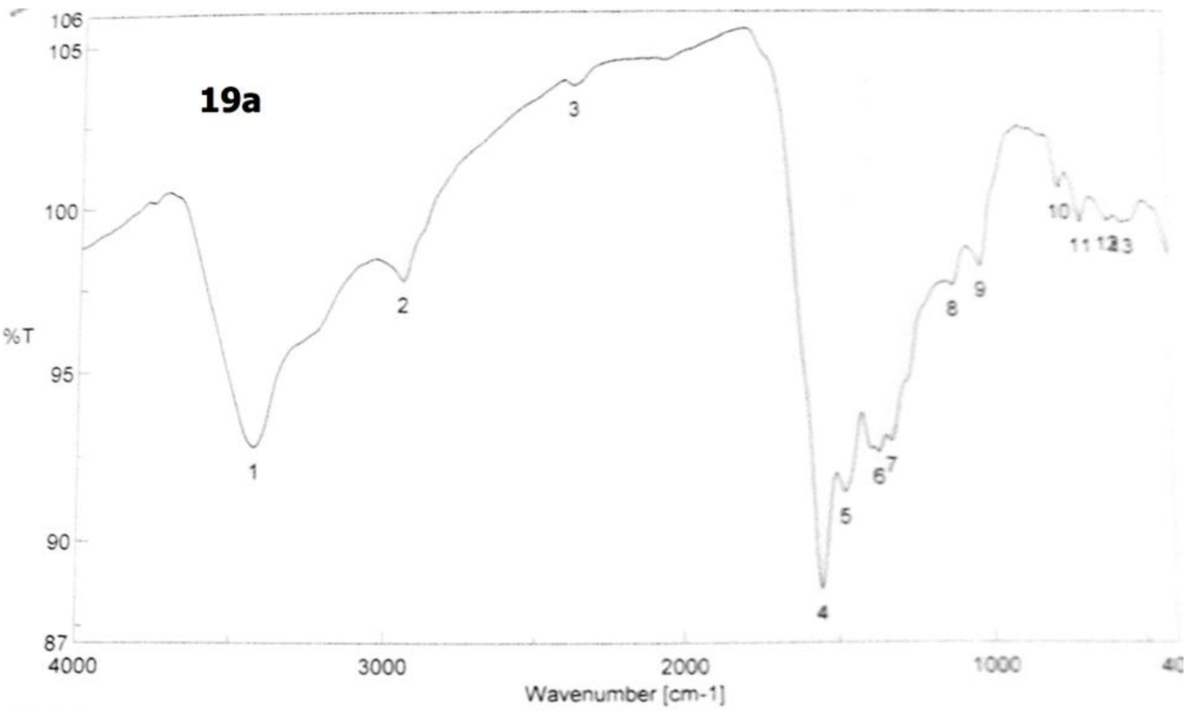

[Comments]

Sample name	SH 11
Comment	6/2018
User	IR
Division	IR
Company	MAC

[Result of Peak Picking]

No.	Position	Intensity	No.	Position	Intensity	No	Position	Intensity
1	3419.17	92.7854	2	2929.34	97.7475	3	2362.37	103.713
4	1554.34	88.5783	5	1480.1	91.4049	6	1370.18	92.566
7	1330.64	92.905	8	1124.3	97.5709	9	1029.8	98.1671
10	760.78	100.58	11	691.355	99.5347	12	601.682	99.6101
13	553.47	99.5343						

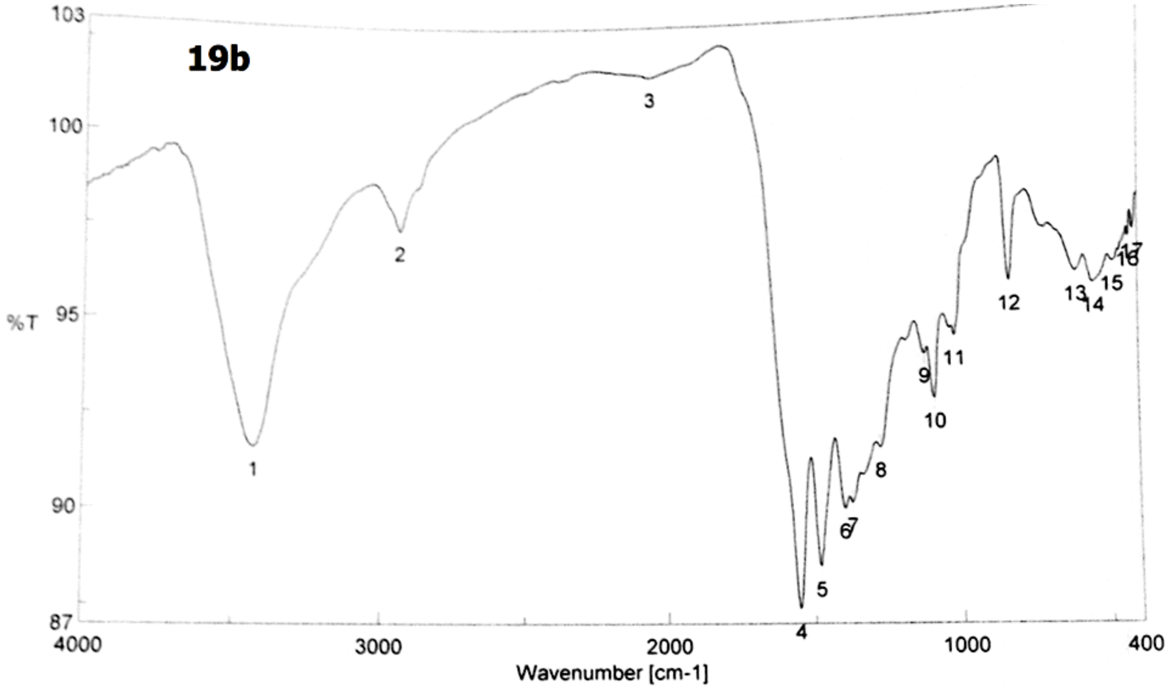

[Comments]

Sample name	SH 13
Comment	6/2018
User	IR
Division	IR
Company	MAC

[Result of Peak Picking]

No.	Position	Intensity	No.	Position	Intensity	No.	Position	Intensity
1	3423.99	91.6275	2	2925.48	97.3724	3	2049.96	101.615
4	1552.42	87.3904	5	1482.03	88.4942	6	1398.14	90.0004
7	1373.07	90.1508	8	1275.68	91.599	9	1121.4	94.0569
10	1089.58	92.8718	11	1018.23	94.5151	12	827.312	95.9205
13	604.574	96.105	14	545.756	95.7886	15	484.045	96.3221
16	431.012	96.9329	17	416.549	97.127			

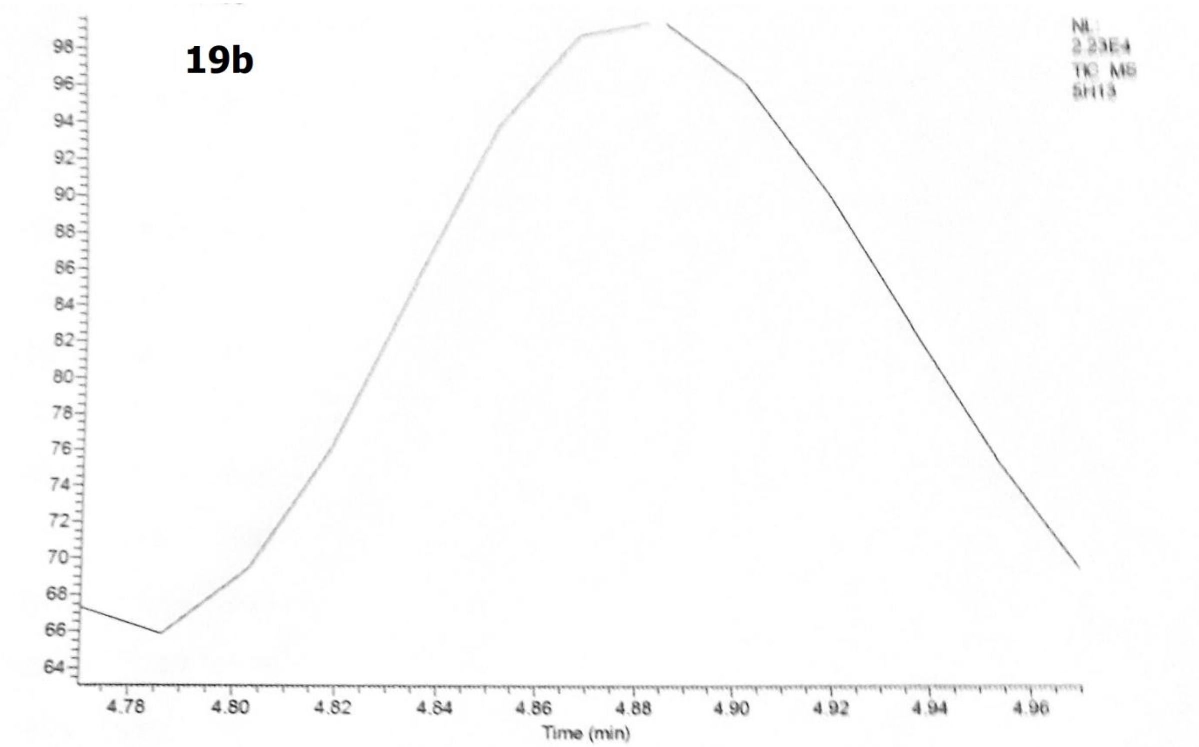
19b
NL:
2.23E4
TIC MS
5H13
Time (min)

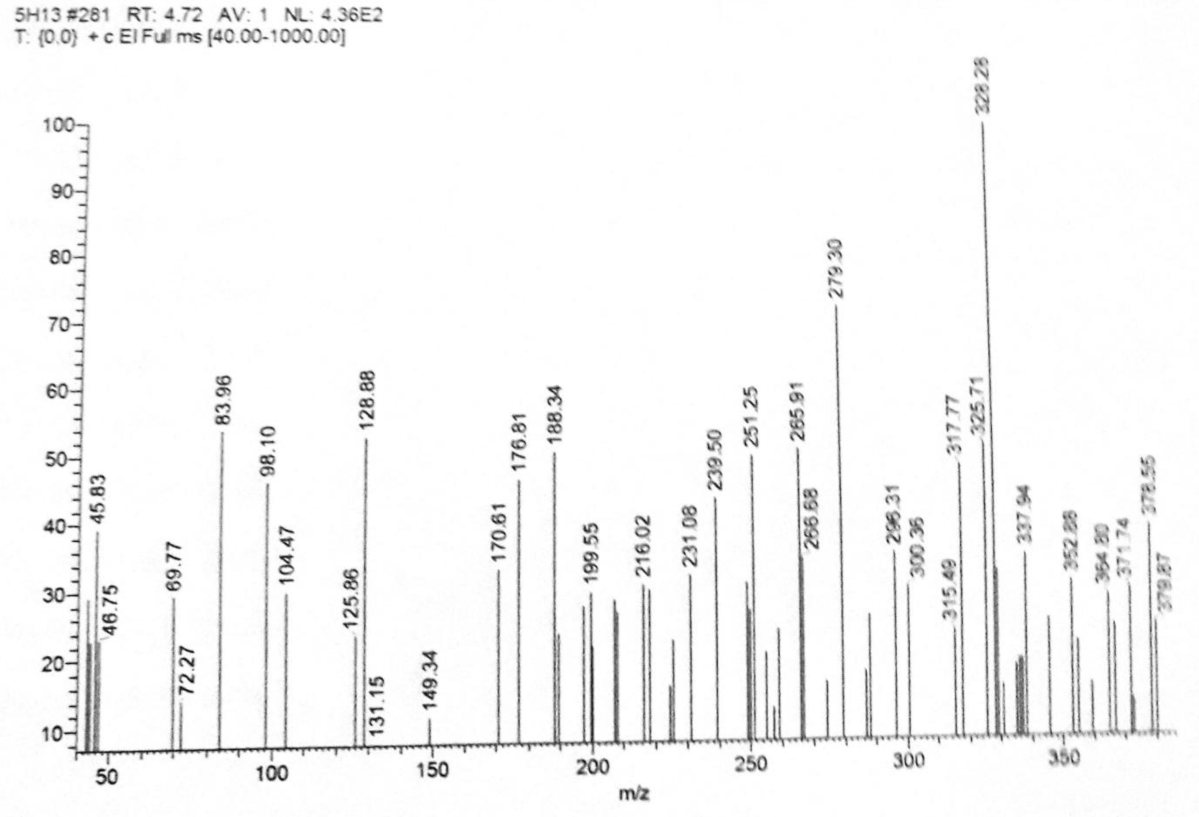
5H13 #281 RT: 4.72 AV: 1 NL: 4.36E2
T: {0,0} + c EI Full ms [40.00-1000.00]
328.28
279.30
83.96
128.88
176.81
188.34
239.50
251.25
265.91
317.77
325.71
98.10
45.83
104.47
170.61
199.55
216.02
231.08
266.68
296.31
300.36
337.94
352.88
378.55
m/z

19c

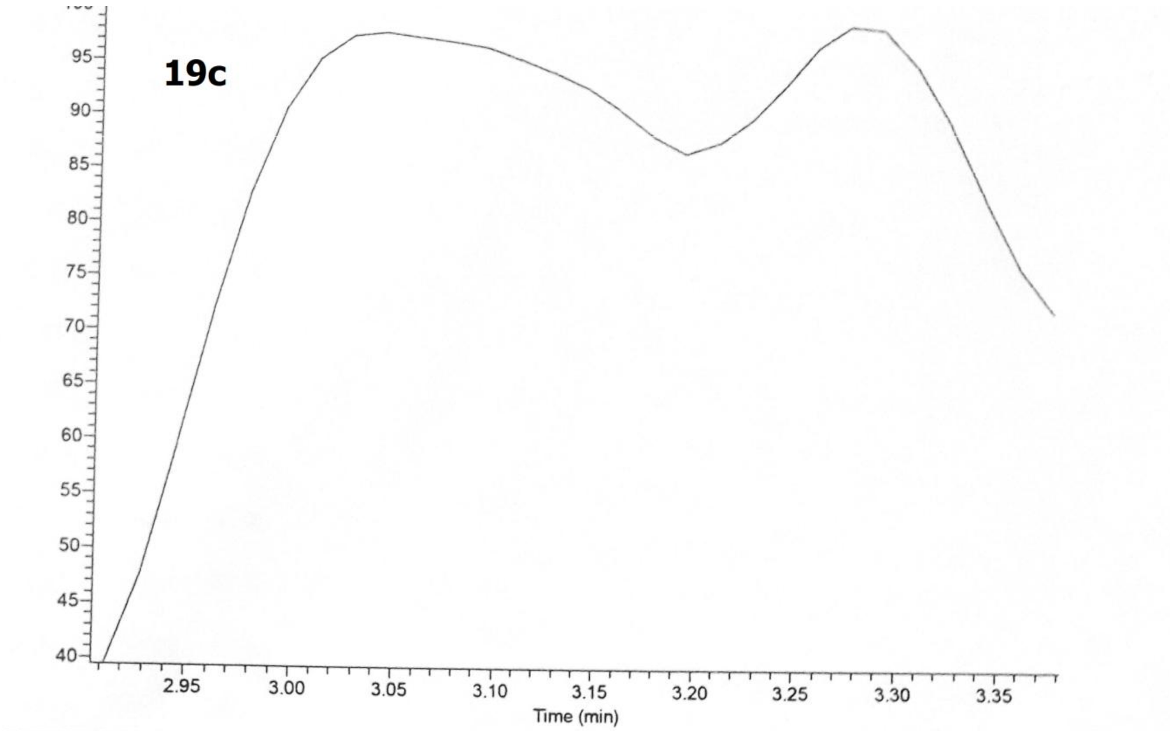
95
90
85
80
75
70
65
60
55
50
45
40
2.95
3.00
3.05
3.10
3.15
3.20
3.25
3.30
3.35
Time (min)

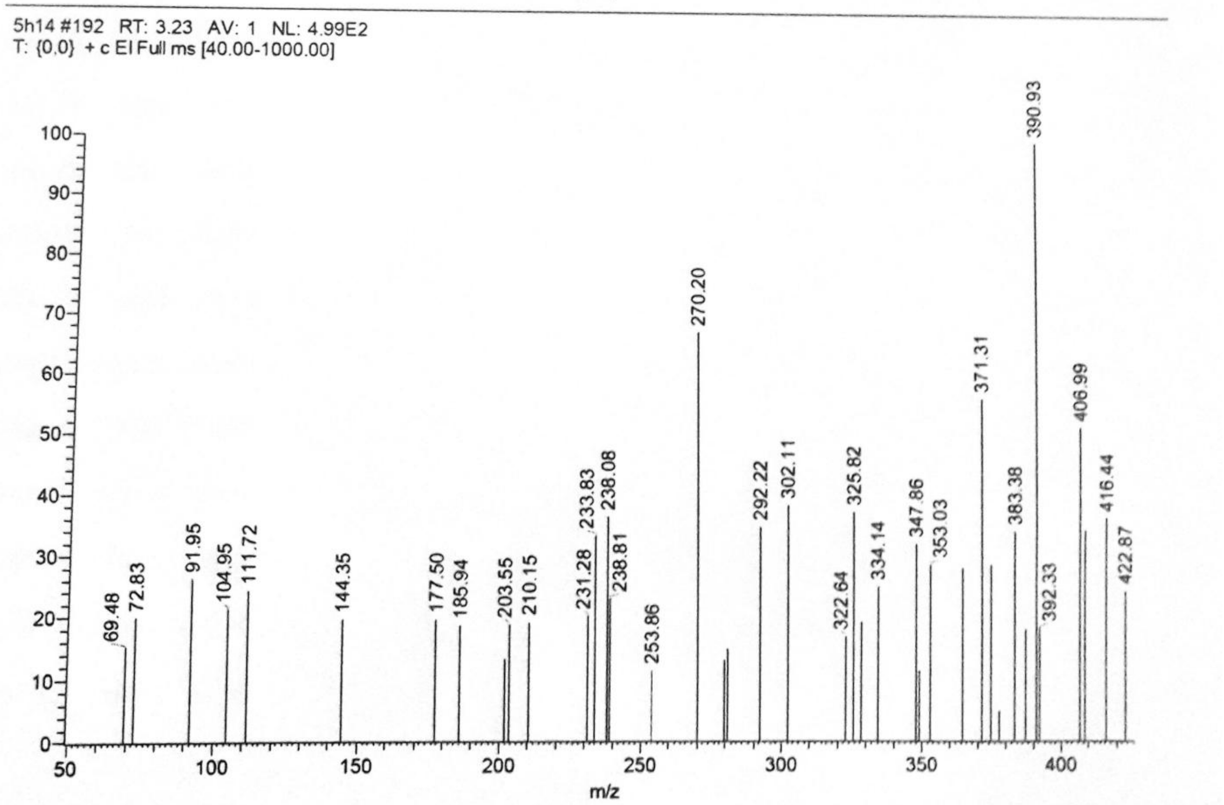
5h14 #192 RT: 3.23 AV: 1 NL: 4.99E2
T: {0,0} + c EI Full ms [40.00-1000.00]
390.93
270.20
371.31
406.99
292.22
302.11
325.82
238.08
233.83
383.38
416.44
347.86
353.03
91.95
111.72
334.14
144.35
177.50
104.95
185.94
203.55
210.15
231.28
238.81
422.87
392.33
72.83
322.64
69.48
253.86
m/z

19d

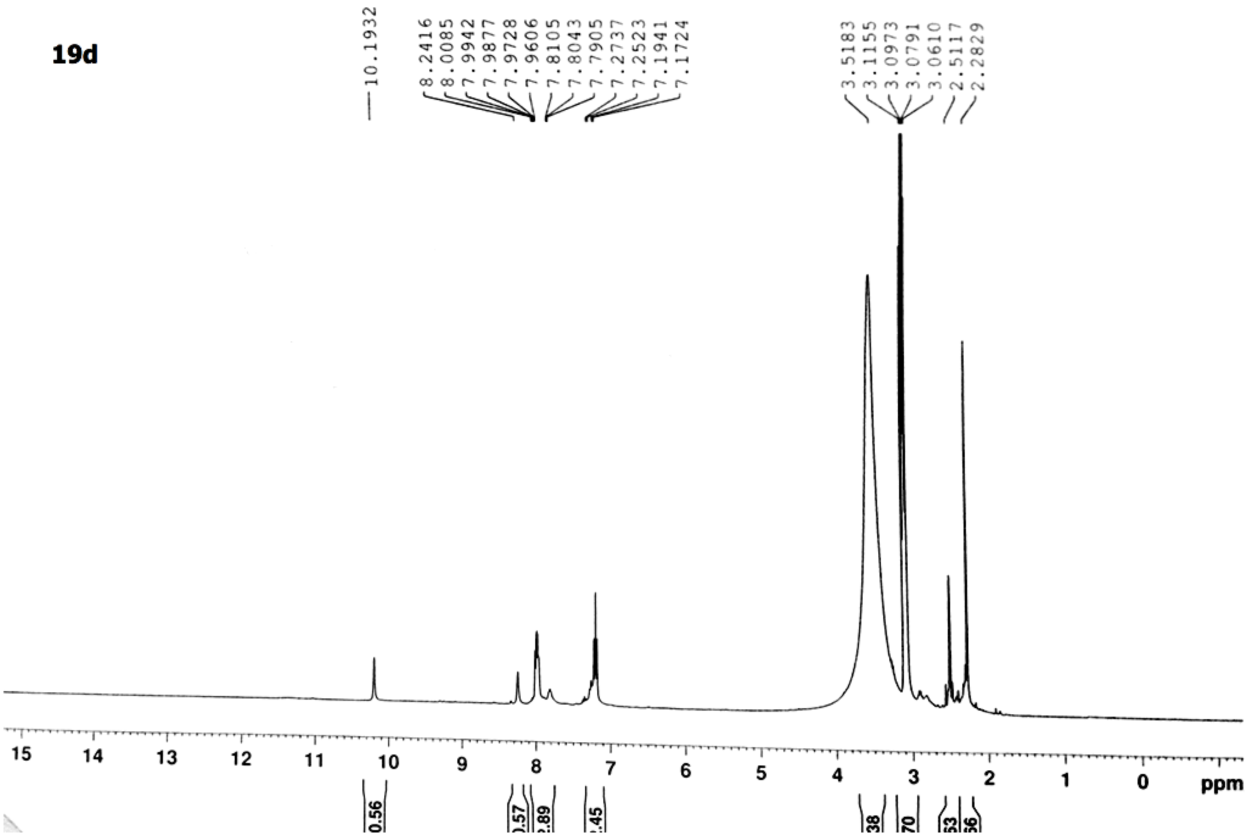

10.1932
8.2416
8.0085
7.9942
7.9877
7.9728
7.9606
7.8105
7.8043
7.7905
7.2737
7.2523
7.1941
7.1724
3.5183
3.1155
3.0973
3.0791
3.0610
2.5117
2.2829
15
14
13
12
11
10
9
8
7
6
5
4
3
2
1
0
ppm
0.56

19e

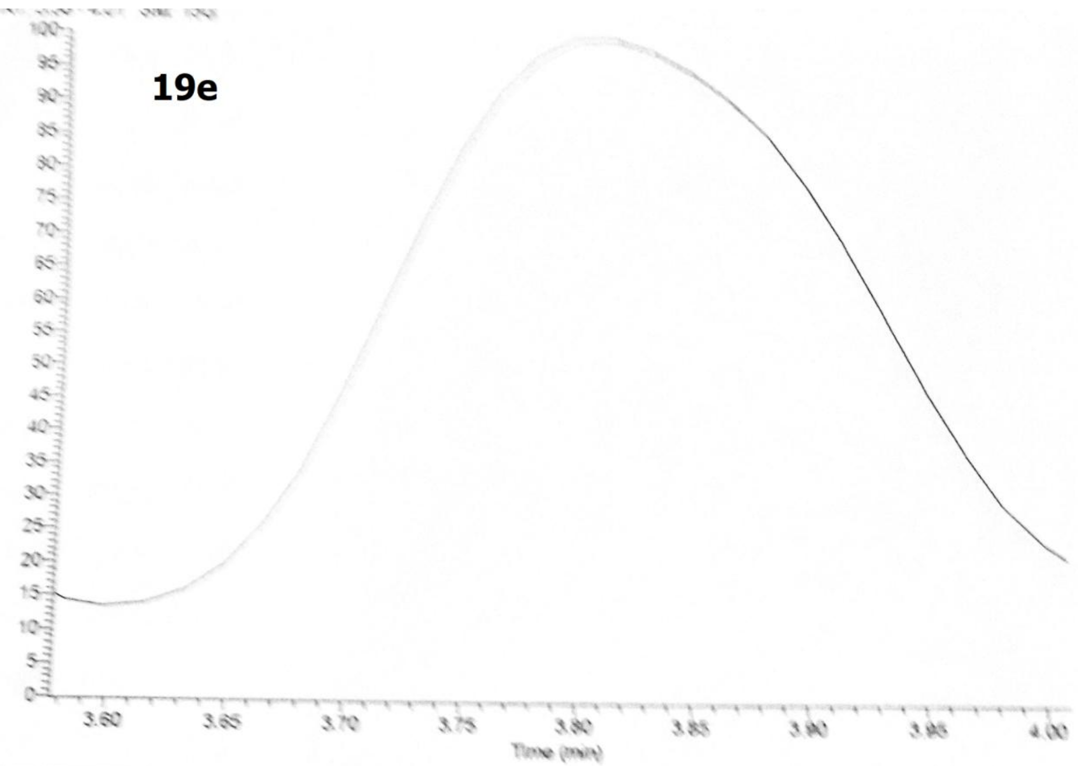
Time (min)
3.60
3.65
3.70
3.75
3.80
3.85
3.90
3.95
4.00

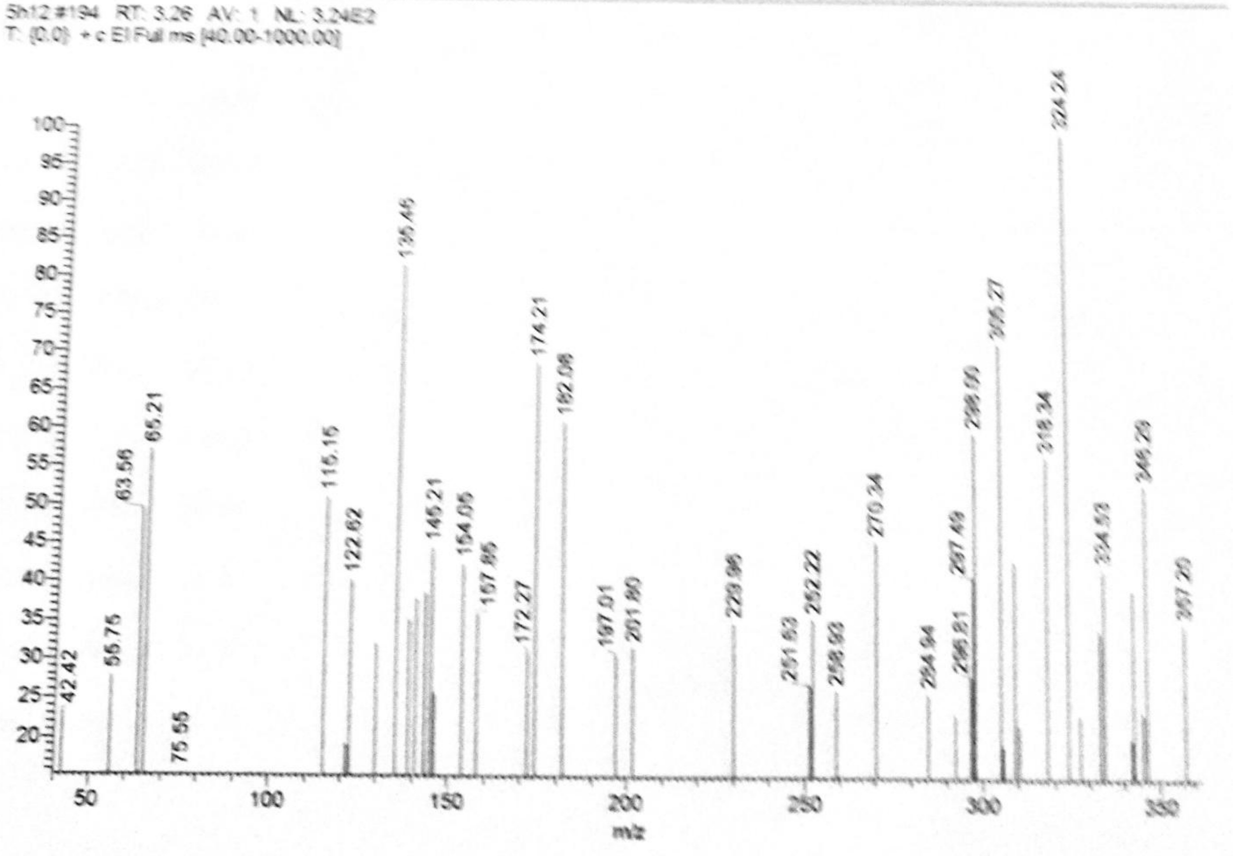
5h12 #194 RT: 3.26 AV: 1 NL: 3.24E2
T: {0,0} + c EI Full ms [40.00-1000.00]
m/z
42.42
55.75
63.56
65.21
75.55
115.15
122.62
135.46
145.21
154.05
157.85
172.27
174.21
182.08
197.01
201.80
229.96
251.63
252.22
258.93
270.34
284.94
296.81
297.49
298.50
305.27
318.34
324.24
334.53
346.25
357.20

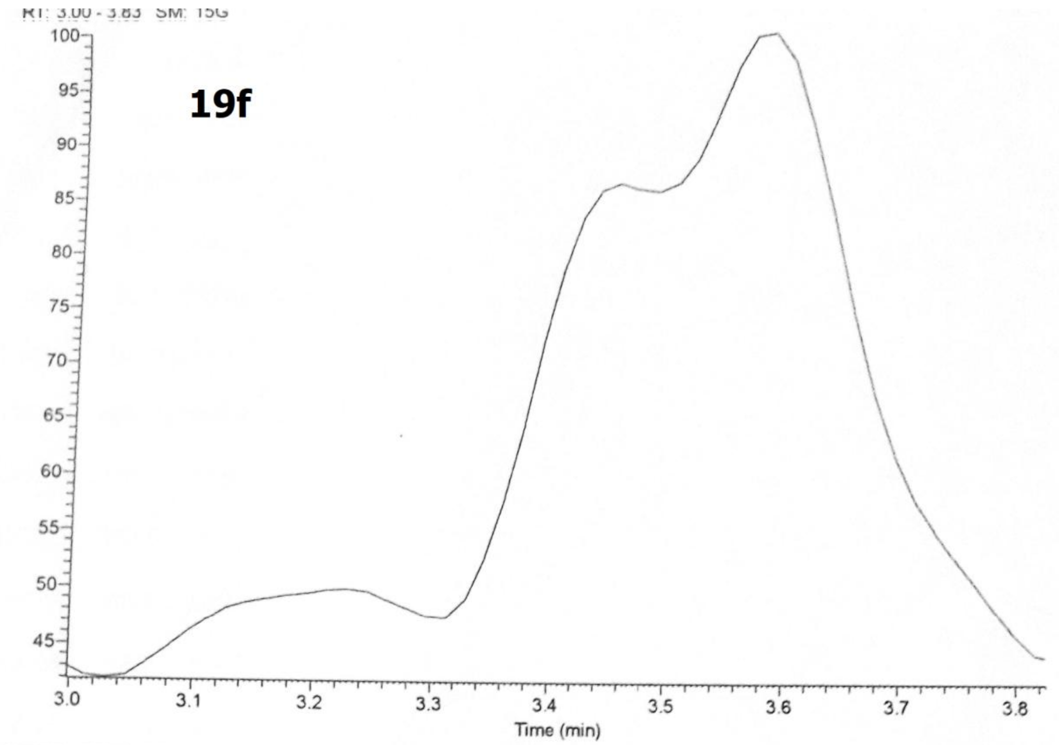
RT: 3.00 - 3.83 SM: 15G
19f
Time (min)

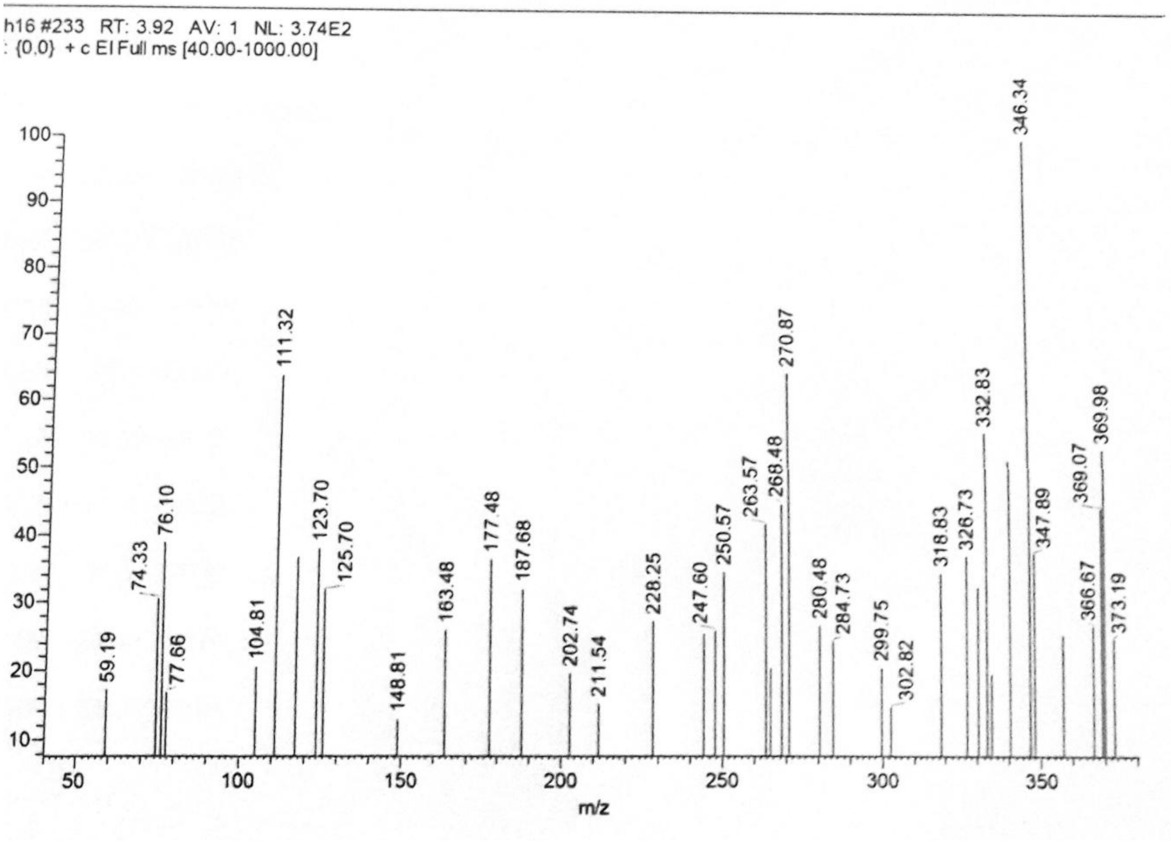
h16 #233 RT: 3.92 AV: 1 NL: 3.74E2
: {0,0} + c EI Full ms [40.00-1000.00]
346.34
111.32
270.87
332.83
369.98
369.07
268.48
263.57
250.57
347.89
m/z

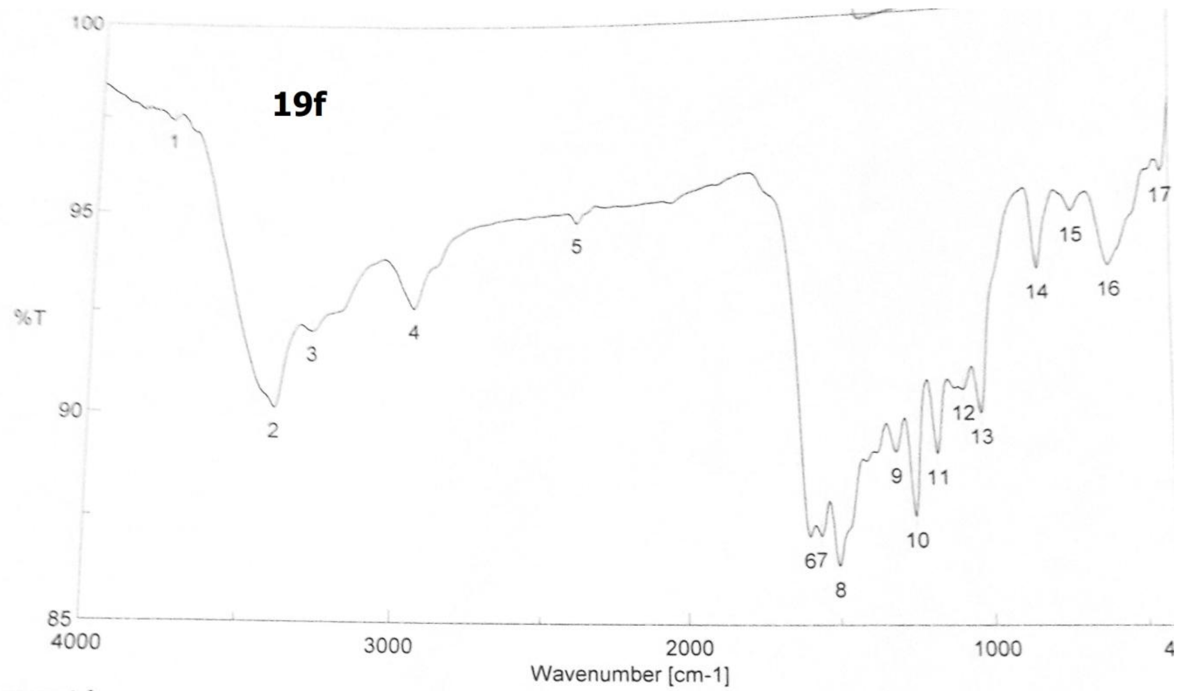

[Comments]
Sample name SH 16
Comment 6/2018
User IR
Division IR
Company MAC

[Result of Peak Picking]

No.	Position	Intensity	No.	Position	Intensity	No.	Position	Intensity
1	3752.8	97.4128	2	3386.39	90.152	3	3265.86	92.0557
4	2926.45	92.6479	5	2374.91	94.8463	6	1597.73	87.1141
7	1560.13	87.1277	8	1502.28	86.4138	9	1311.36	89.1524
10	1249.65	87.6011	11	1175.4	89.1103	12	1086.69	90.6569
13	1028.84	90.0818	14	832.133	93.5772	15	717.39	94.9346
16	597.825	93.6022	17	427.155	95.7834			

21b

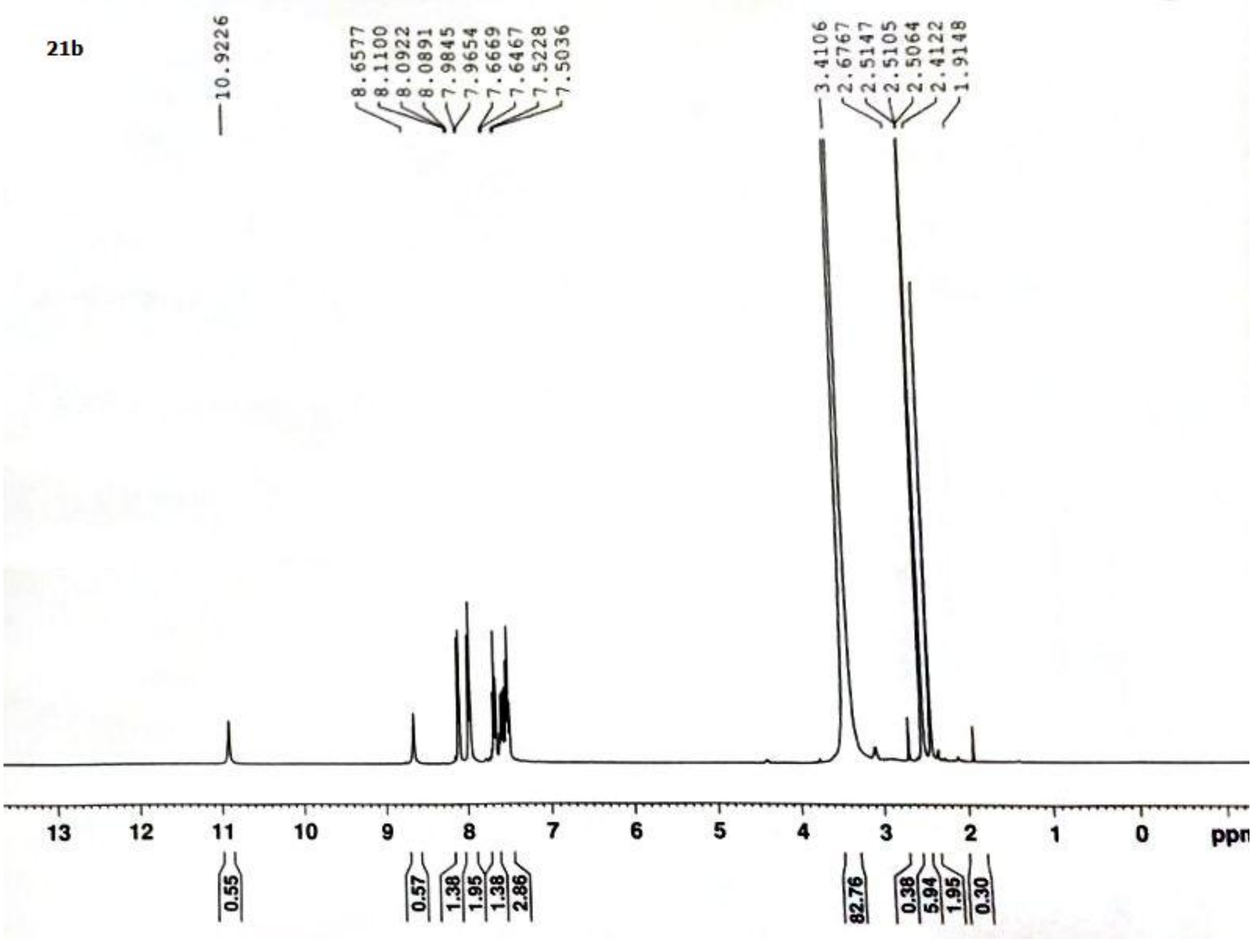
10.9226
8.6577
8.1100
8.0922
8.0891
7.9845
7.9654
7.6669
7.6467
7.5228
7.5036
3.4106
2.6767
2.5147
2.5105
2.5064
2.4122
1.9148
13 12 11 10 9 8 7 6 5 4 3 2 1 0 ppm
0.55
0.57
1.38
1.95
1.38
2.86
82.76
0.38
5.94
1.95
0.30

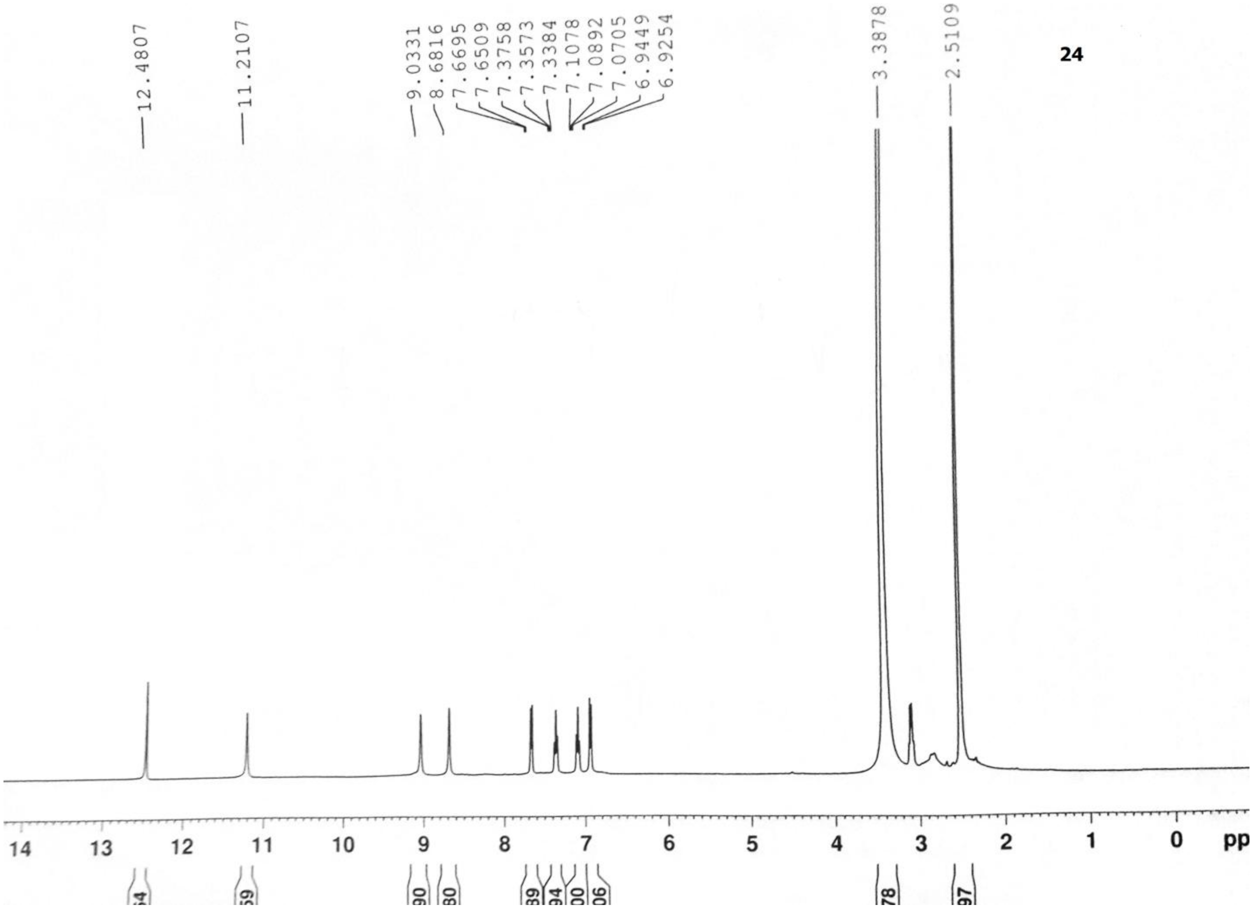
12.4807
11.2107
9.0331
8.6816
7.6695
7.6509
7.3758
7.3573
7.3384
7.1078
7.0892
7.0705
6.9449
6.9254
3.3878
2.5109
14 13 12 11 10 9 8 7 6 5 4 3 2 1 0 pp

24

24

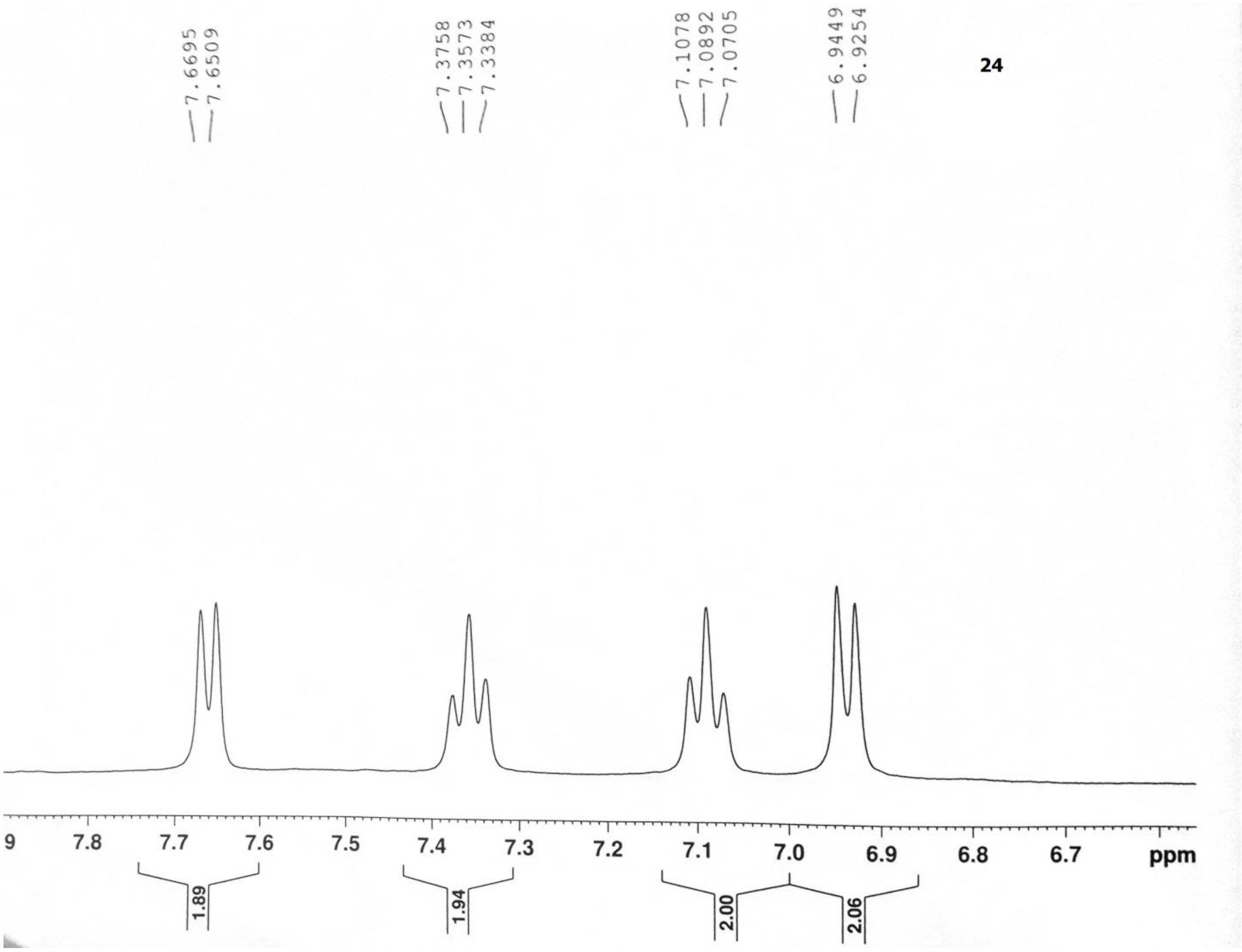
7.6695
7.6509
7.3758
7.3573
7.3384
7.1078
7.0892
7.0705
6.9449
6.9254
9
7.8
7.7
7.6
7.5
7.4
7.3
7.2
7.1
7.0
6.9
6.8
6.7
ppm
1.89
1.94
2.00
2.06

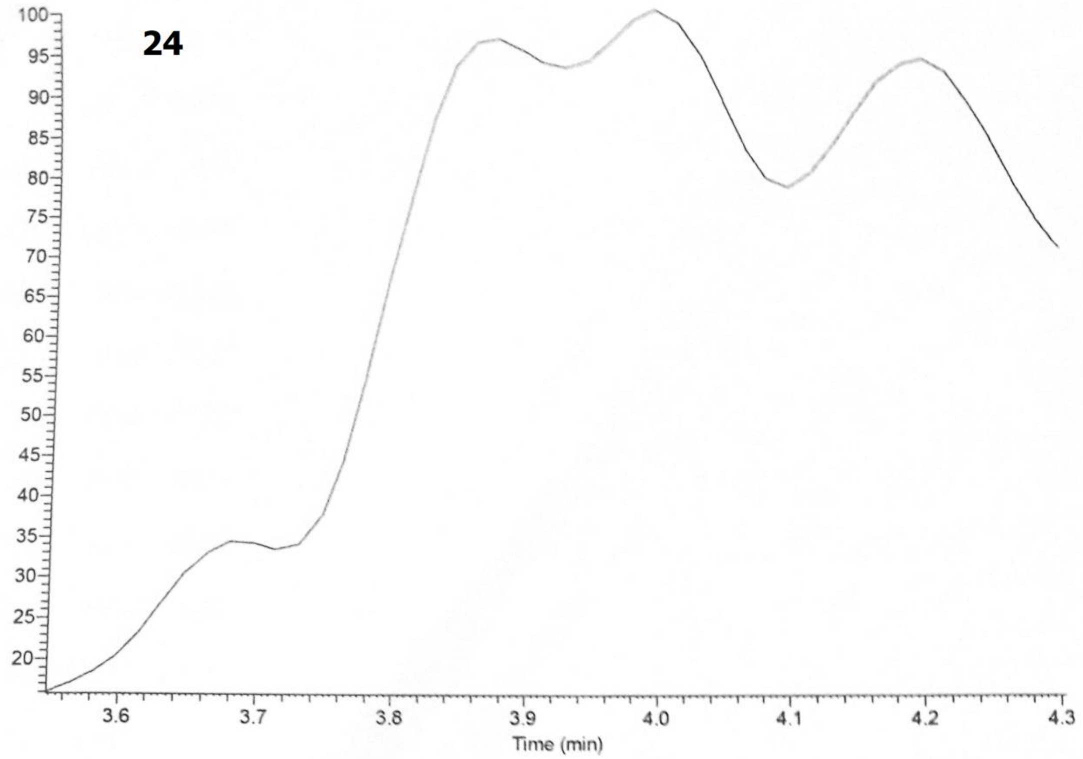

5h22 #250 RT: 4.20 AV: 1 NL: 5.19E2
T: {0,0} + c EI Full ms [40.00-1000.00]

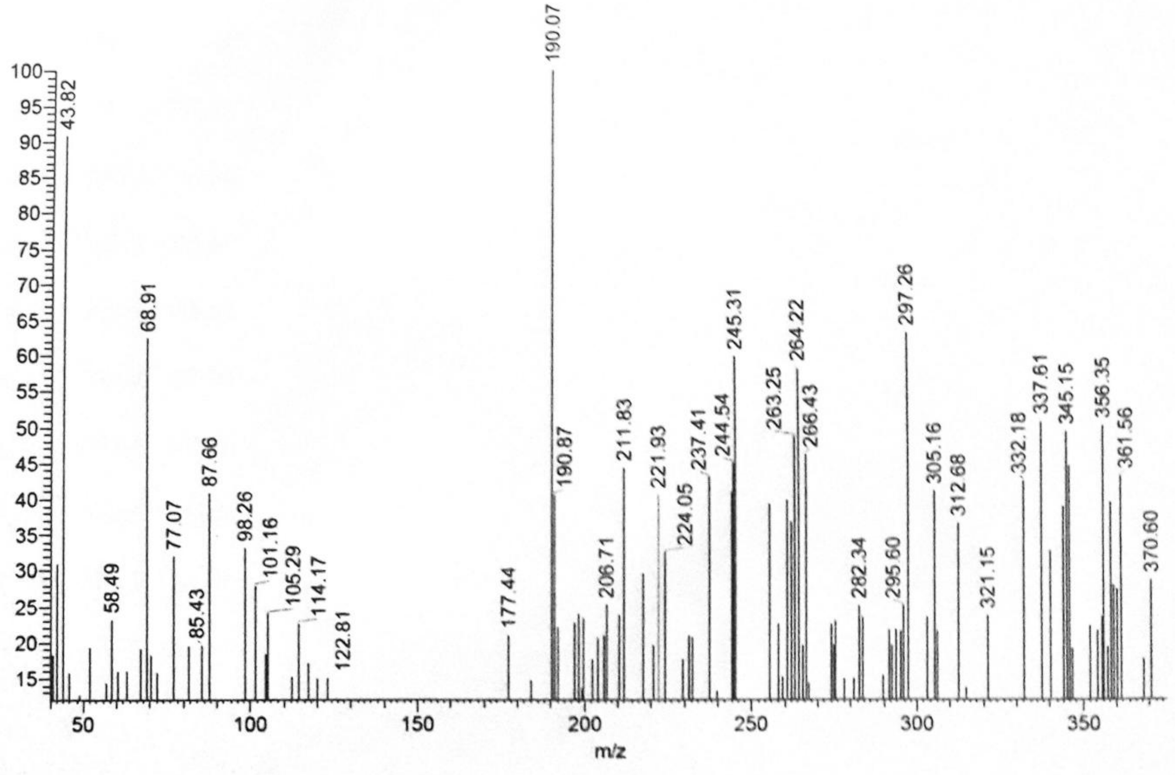

Supplement: Supplementary file 1 [file molecules-24-01741-s001.pdf]
